# Tumor Neoepitope-Based Vaccines: A Scoping Review on Current Predictive Computational Strategies

**DOI:** 10.3390/vaccines12080836

**Published:** 2024-07-24

**Authors:** Luiz Gustavo do Nascimento Rocha, Paul Anderson Souza Guimarães, Maria Gabriela Reis Carvalho, Jeronimo Conceição Ruiz

**Affiliations:** 1 Biologia Computacional e Sistemas (BCS), Instituto Oswaldo Cruz (IOC), Fundação Oswaldo Cruz, Rio de Janeiro 21040-900, Brazil; luizbio2408@gmail.com (L.G.d.N.R.); contato@paulanderson.com.br (P.A.S.G.); 2 Grupo Informática de Biossistemas e Genômica, Instituto René Rachou, Fundação Oswaldo Cruz, Belo Horizonte 30190-002, Brazil

**Keywords:** neoepitope, algorithm, personalized therapy, cancer vaccine, tumor, variant calling

## Abstract

Therapeutic cancer vaccines have been considered in recent decades as important immunotherapeutic strategies capable of leading to tumor regression. In the development of these vaccines, the identification of neoepitopes plays a critical role, and different computational methods have been proposed and employed to direct and accelerate this process. In this context, this review identified and systematically analyzed the most recent studies published in the literature on the computational prediction of epitopes for the development of therapeutic vaccines, outlining critical steps, along with the associated program’s strengths and limitations. A scoping review was conducted following the PRISMA extension (PRISMA-ScR). Searches were performed in databases (Scopus, PubMed, Web of Science, Science Direct) using the keywords: neoepitope, epitope, vaccine, prediction, algorithm, cancer, and tumor. Forty-nine articles published from 2012 to 2024 were synthesized and analyzed. Most of the identified studies focus on the prediction of epitopes with an affinity for MHC I molecules in solid tumors, such as lung carcinoma. Predicting epitopes with class II MHC affinity has been relatively underexplored. Besides neoepitope prediction from high-throughput sequencing data, additional steps were identified, such as the prioritization of neoepitopes and validation. Mutect2 is the most used tool for variant calling, while NetMHCpan is favored for neoepitope prediction. Artificial/convolutional neural networks are the preferred methods for neoepitope prediction. For prioritizing immunogenic epitopes, the random forest algorithm is the most used for classification. The performance values related to the computational models for the prediction and prioritization of neoepitopes are high; however, a large part of the studies still use microbiome databases for training. The in vitro/in vivo validations of the predicted neoepitopes were verified in 55% of the analyzed studies. Clinical trials that led to successful tumor remission were identified, highlighting that this immunotherapeutic approach can benefit these patients. Integrating high-throughput sequencing, sophisticated bioinformatics tools, and rigorous validation methods through in vitro/in vivo assays as well as clinical trials, the tumor neoepitope-based vaccine approach holds promise for developing personalized therapeutic vaccines that target specific tumor cancers.

## 1. Introduction

Personalized cancer therapy has been recommended primarily when the patient’s treatment prognosis is unfavorable, whether due to the type of tumor or the disease stage. Personalized therapy consists of identifying patient-specific tumor antigens and developing a treatment that stimulates a specific immune response against the tumor. This response can be achieved through the development of cancer vaccines, usually applied in combination with other chemotherapies [1].

The formulation of a cancer vaccine comprises a series of approaches that aim to generate or amplify an antitumor immune response. To achieve this objective, the identification of neoepitopes plays a critical role and can be accomplished through prediction algorithms [2].

Neoepitopes arise from non-synonymous mutations in the tumor genome and result in small, mutated peptides that are presented on major histocompatibility complex (MHC) molecules exclusively on the surface of tumor cells [3].

The performance of these predictive algorithms can be enhanced when integrated with other computational methods addressing complementary, yet critical, biological aspects associated with the immunogenic neoepitopes identification. Among them are the mutation burden, gene expression, peptide transport and cleavage, homolog search, B and T cell activation, and antigen-presenting cells (APCs) prediction algorithms [4].

Among the prominent approaches for analyzing and integrating these data, immunoinformatics stands out as a sub-area of bioinformatics that includes the study and development of algorithms and programs for predicting potential T and B lymphocyte epitopes.

Reverse vaccinology, which is closely related and synergistic to the development of new vaccines, is an in silico method that uses genomic data to identify potential epitopes through computational algorithms.

This innovative approach enables researchers to analyze the entire genome of a pathogen or cancer cells to pinpoint antigenic peptides that could provoke an immune response. By systematically identifying these epitopes, reverse vaccinology facilitates the design of new vaccines that are more precise and effective. This method has revolutionized vaccine development by significantly accelerating the identification process and enhancing the potential for developing targeted immunotherapies [5]. It is important to highlight that epitopes are allele-specific, and the identification of different human leukocyte antigen (HLA) alleles represents a critical step in the process [6]. HLA molecules (MHC in humans) are encoded by highly polymorphic genes located on human chromosome 6 and are surface proteins involved in the induction and regulation of the immune response, serving as biochemical markers for each individual. While class I HLA molecules have three loci (HLA-A, HLA-B, and HLA-C) that have affinities with 8–12 amino acid epitopes, class II HLA molecules also have three loci (HLA-DR, HLA-DP, and HLA-DQ) that have affinities with 15–24 amino acid epitopes [7,8].

Particularly concerning cancer, much progress has been made in understanding Mendelian disorders, identifying prognostic biomarkers, determining optimal therapeutic drug combinations, and establishing correlations between identified variants and clinical phenotypes [9,10] through the integration of data from different initiatives such as “The Cancer Genome Atlas Program” [11] and UK10K [12], among others.

From an experimental point of view, these neoepitopes are generally administered in association with antigen-presenting cells (APCs) or other immune modulators. This involves immunization with shared tumor antigens expressed by many different patients’ tumors and the identification of proteins that were overexpressed in tumor cells but minimally expressed in normal tissues [13]. Traditionally, empirical approaches have been employed; however, the utilization of computational methodologies has the potential to decrease the time required for vaccine development and minimize animal experimentation [14].

Considering the perspective of using NGS-derived data in cancer vaccine development and personalized medicine, the molecular characterization of cancer (whole genome, exome, and transcriptome sequencing, computational approaches determining mutational profile and gene expression) has revealed a complexity and tumor diversity that impact patient prognosis and recommended treatment [15]. It is important to highlight that different specific treatments vary according to disease stage, cancer type, and the mutational burden of the tumor tissue. As each tumor possesses a unique mutation profile [16], the latter has been more recently explored and the identification of tumor-specific mutations can lead to the identification of vaccine targets in personalized therapies [5].

Lastly, one additional challenging aspect that must be considered for the efficient development of a tumor neoepitope-based vaccine is the quality of the training data used by the algorithms. Neoepitope predictors mainly use machine learning methods (e.g., artificial neural network, convolutional neural network, random forest), which still exhibit a high false positive rate (estimated around 50% to 95%) [3].

A contributing factor to this performance variation is the utilization of pathogen training data by most algorithms, which clearly does not accurately reflect the human cancer data necessary for effective vaccine prospecting [17]. Taking into account the aforementioned scenario and aiming to enhance the effective and rational application of computational methods for vaccine prospecting, a scoping review was conducted to offer a comprehensive analysis of scientific evidence, address emerging topics, and pinpoint knowledge gaps in this field.

In this scoping review, our objective is to identify the prevalent computational methods utilized by the scientific community for predicting neoepitopes in human high-throughput data sequencing. Additionally, we aim to explore how these methods incorporate the biological intricacies inherent in the immune response against tumor antigens and the development of therapeutic vaccines. To achieve this, we followed the guiding questions outlined as follows: What are the critical steps in identifying immunogenic neoepitopes? What are the main types of tumors studied? What algorithms and programs have been employed for predicting tumor neoepitopes? What dataset is used as a training model? How are the predictions validated? What are the strengths and limitations of the employed approaches? Can this prediction accurately guide the identification of potential targets for the development of therapeutic vaccines?

## 2. Methods

To achieve the proposed objective and address the previously described questions, a scoping review was conducted following the PRISMA extension guidelines for scoping reviews (PRISMA-ScR) [18].

We conducted a search and included studies that aimed to identify neoepitopes in human tumor samples sequencing data using computational approaches. Specifically, we sought articles that identified neoepitopes in human next-generation sequencing (NGS) data through predictive algorithms, as well as whether there was any prioritization step for immunogenic neoepitopes for vaccine composition. Additionally, we looked for articles that presented the development of new programs or algorithms that could be utilized for the identification of neoepitopes in human tumor samples sequencing data.

Articles that analyzed cancer data linked to viral infection were excluded since they involve epitopes of viral origin, distinct from neoepitopes originating from somatic mutations in human wild-type sequences. Additionally, articles were excluded if they did not utilize next-generation sequencing (NGS) data or focused solely on previously studied genes. Moreover, articles lacking detailed descriptions of the in silico analysis stage or employing methods irrelevant to this review were excluded. Studies involving murine models and those falling outside the scope of interest were also excluded.

The retrieved articles were categorized into two synthesis matrices: one focusing on studies conducted with existing algorithms and programs, and the other concentrating on studies that explore the development of novel algorithms and programs.

The articles were retrieved from four databases: Scopus, PubMed, Web of Science, and Science Direct. These databases were selected for their comprehensive coverage of the literature in medical, biological, and computational sciences. The search across all databases was conducted on 24 January 2024. For each database, articles published in English between January 2012 and January 2024 were included. The following filters were used:

- Scopus: article type, abstract, keywords; all open access; publication stage: final; article journal.
- Pubmed: free full text, full text.
- Web of science: open access; articles; English language.
- Science Direct: open access and open archive; research article (search in “title, abstract, keywords”).

To determine which studies should be included in the review, Boolean operators were utilized for the search, employing the keywords “neoepitope”, “epitope”, “vaccine”, “prediction”, “algorithm”, “cancer”, and “tumor”: (neoepitope OR epitope) AND vaccine AND (prediction OR algorithm) AND (cancer OR tumor). Only complete, original, and open access publications were considered for inclusion. Any duplicate publications were promptly excluded. During this process, two reviewers independently screened each retrieved record. Any disagreements regarding study selection and data extraction were resolved through discussion and review of the search parameters. The selection of studies and determination of variables to extract were also conducted and discussed by these two independent reviewers. Data collection from the articles and the screening process were managed using the software Zotero (version 6, for Windows). The screening process followed a two-phase protocol. In the first phase, the titles and abstracts of the articles were read to select those that would be subjected to a full reading. 

In the subsequent phase, a comprehensive examination of the introduction, methods, results, and conclusion sections was conducted. Publications that did not present a computational methodology for neoepitope identification in next-generation sequencing (NGS) data or solely focused on a specific gene were excluded.

Finally, from the selected studies, a synthesis matrix was compiled, covering qualitative aspects such as study design (data type employed, type of tumor), key program and algorithm applications (variant calling and neoepitope prediction), evaluated immune attributes, validations, benefits, and drawbacks.

To determine the data to collect, we focused on aspects directly related to the process of identifying immunogenic neoepitopes and the success rate of the programs used. During the review process, two main types of studies were identified. One type presents the identification of neoepitopes using existing programs and algorithms from the literature, while the other type involves the development of new tools for neoepitope identification, both utilizing next-generation sequencing (NGS) data.

In studies that utilized programs already existing in the literature, the following data were collected:

1. Dataset (next-generation sequencing data sequencing)
2. Type of tumor
3. Variant calling
4. Neoepitope prediction program
5. Alleles of MHC-I and II
6. Statistics (the score used to classify epitopes)
7. Evaluated immunological characteristics (biological and biochemical aspects affecting the immune response)
8. Whether a structural model was evaluated
9. Whether in vitro and in vivo validations were performed
10. Positive/negative aspects

In studies where new programs or algorithms were designed, the following data were collected:

1. Whether mutational data were evaluated
2. Length of MHC-I and II epitopes
3. Algorithm/matrix used
4. Training data (tumor or microbiome)
5. Steps of prioritization of neoepitopes
6. Performance

Here, we differentiate between algorithms and programs as follows: An algorithm refers to the mathematical and statistical method used. A program is the code written to allow the algorithm’s function. Software is an architecture built to utilize one or more programs and manipulate data. All data and methods used in each study were synthesized into a matrix for visualization and analysis. This matrix allowed for a comprehensive overview of the key variables and approaches employed across the selected studies, facilitating comparison and interpretation of the findings.

## 3. Results and Discussion

**Figure 1:**
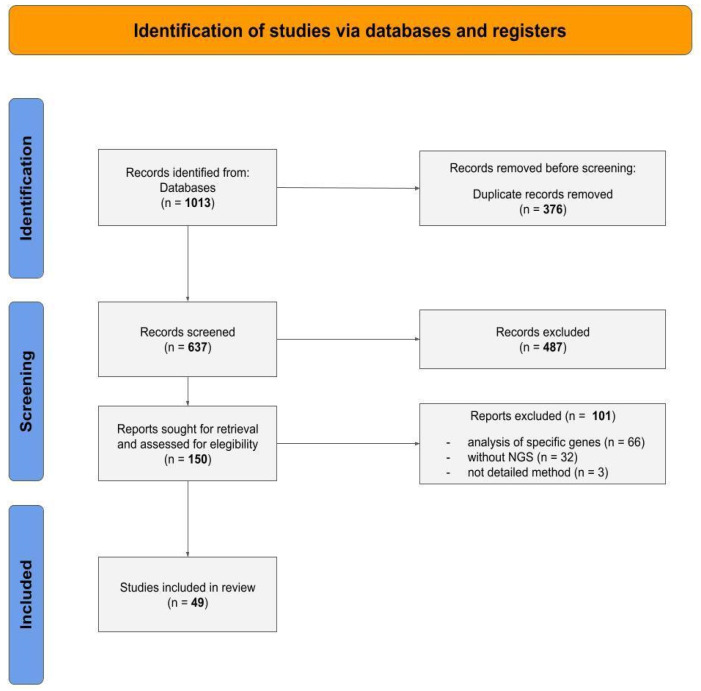
PRISMA flow diagram.

The number of articles retrieved from all databases totaled 1013, comprising 16 from Science Direct, 494 from PubMed, 257 from Web of Science, and 246 from Scopus. After eliminating duplicates across databases, 637 articles remained. An evaluation of the titles and abstracts of these articles was conducted, resulting in the exclusion of 487 articles that did not meet the established criteria. Consequently, 150 articles were selected for full-text reading, including an examination of the introduction, methods, results, discussion, and conclusion sections. Among these, 101 articles were excluded for not presenting a study model based on human tumor NGS data. All articles removed during the screening process are listed in Table S1. Ultimately, 49 articles were included in this review. Figure 1 illustrates the PRISMA flow diagram, summarizing the article selection and exclusion process.

Out of the remaining 49 articles, a synthesis matrix was formulated and divided into two segments corresponding to the applications discussed in the studies.

The initial section encompasses articles that utilize tumor next-generation sequencing (NGS) data and implement computational methodologies. This includes the utilization of neoepitope prediction algorithms in testing these sequences to generate an immune response and/or in designing a therapeutic vaccine.

The subsequent section compiles articles that focus on pioneering a new computational methodology for neoepitope identification.

Table 1 provides an overview of the first section of the matrix, while Table 2 provides an overview of the second section of the matrix.

### 3.1. Critical Steps in Identifying Immunogenic Neoepitopes

**Figure 2:**
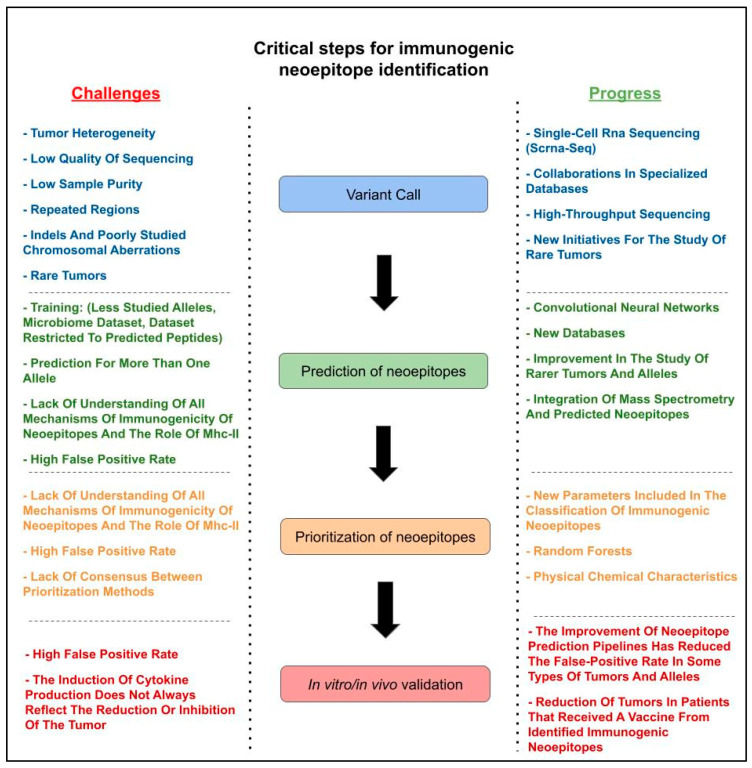
Critical steps flowchart. The figure presents the challenges (**left**) and current progress (**right**) of each of the critical steps in the identification of immunogenic neoepitopes.

To address the guiding question “What are the critical steps in identifying immunogenic neoepitopes?”, we identify these critical steps and summarize the main aspects for each of them. According to the results of the first section of the synthesis matrix [19,20,21,22,23,24,25,26,27,28,29,30,31,32,33,34,35,36,37,38,39,40,41], the critical steps are (a) variant calling; (b) the prediction of neoepitopes; (c) the prioritization of neoepitopes; and (d) in vitro and in vivo validation. In contrast, the second section of the matrix [42,43,44,45,46,47,48,49,50,51,52,53,54,55,56,57,58,59,60,61,62,63,64,65,66,67], which focuses on articles addressing the development of new algorithms and programs, identified the main steps as (a) the prediction of neoepitopes; and (b) the prioritization of neoepitopes. Figure 2 presents a flowchart with the critical steps identified, challenges, and progress.

In variant calling, mutations with the potential to alter the gene product were identified. This step is crucial when working with sequencing data, such as whole-genome sequencing (WGS), whole-exome sequencing (WES), or RNA-seq, as it directly impacts epitope prediction.

The prediction of neoepitopes involves using algorithms to analyze genes with inserted mutations. These algorithms predict the binding affinity of the peptide to the MHC molecule and assess other variables that impact its immunogenicity.

The prioritization of neoepitopes is the subsequent step, where the most promising neoepitopes are selected based on their predicted immunogenicity and other relevant factors.

Finally, the validation of the immunogenicity of neoepitopes is performed to test the validity of the in silico steps performed previously. This validation step is crucial for confirming the efficacy of the predicted neoepitopes in eliciting an immune response.

These critical stages in the neoepitope prediction process will be discussed in more detail below, connecting them with the review questions and objectives.

### 3.2. Main Types of Tumors Studied and Variant Calling

In the context of the guiding question “What are the main types of tumors studied?”, we explore the most studied types of tumors and their mutation frequency and investigate the challenges posed by tumor heterogeneity in the critical step of variant calling.

#### 3.2.1. Mutation Frequency

The mutation frequency of tumor cells varies depending on the tissue of origin, with some types of tumors exhibiting higher mutation frequencies than others. One of the factors that result in this increase in the mutation rate of a cell type is the degree of exposure of the tissue to mutagenic agents. For example, cells in the lung and skin, which are organs with a surface facing the external environment, and are exposed to mutagenic agents, like tobacco and UV radiation, respectively, typically show the highest mutation rates. Other factors such as toxins and chronic inflammation can also increase cellular stress, leading to increased mutation rates [68]. 

**Figure 3:**
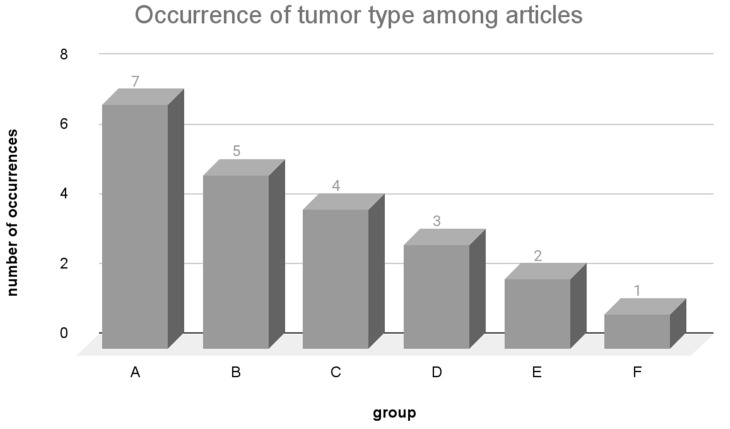
The occurrence of the type of tumor among articles. The horizontal (x) axis represents different groups of types of tumors, while the vertical (y) axis indicates the number of occurrences of each type of tumor across the articles. (**A**) lung carcinoma; (**B**) breast carcinoma, colorectal carcinoma, liver carcinoma, melanoma, ovarian carcinoma; (**C**) gastric adenocarcinoma; (**D**) glioblastoma, pancreatic carcinoma; (**E**) B-cell lymphocytic leukemia, glioma, neuroblastoma, prostate carcinoma; (**F**) Burkitt’s lymphoma, cervical adenocarcinoma, cholangiocarcinoma, chronic lymphocytic leukemia, chronic myeloid leukemia, colorectal adenocarcinoma, embryonal, endometrial, mesothelioma, myeloblastoma, osteosarcoma, renal carcinoma, squamous cell carcinoma, teratoid/rhabditoid tumor.

Consequently, tumors with a high mutation rate can generate a large number of potential neoantigen candidates for vaccine development [30]. However, even in tumors with a low mutation rate, neoepitopes still have the potential to induce specific immune responses through immunotherapy or therapeutic vaccines [36,69]. Figure 3 illustrates how each of the types of tumors were grouped (A, B, C, D, E, F) according to the number of articles where they were studied in the review. Each of the groups aggregated types of tumors with the same frequency of use among the articles. Supplementary Table S2 shows the articles where each type of tumor is found.

Among the analyzed articles, lung carcinoma was the most extensively studied type of tumor, featured in seven articles (group A). Group B had five articles, and the focus was on breast carcinoma, colorectal carcinoma, liver carcinoma, melanoma, and ovarian carcinoma. In the case of group C, covered by four articles, the research centered around gastric adenocarcinoma. Group D, explored in three articles, encompassed glioblastoma and pancreatic carcinoma. Group E, covered by two articles, studied B-cell lymphocytic leukemia, glioma, neuroblastoma, and prostate carcinoma. With tumors investigated in one article, group F encompassed a range of less commonly studied tumors, predominantly highlighted by a solitary article [19] that utilized database samples.

As reported by Martínez-Pérez in 2021 [70], discussing the average mutations per collected tumor sample from COSMIC, it becomes evident that many of the most extensively studied tumors also exhibit the highest mutation burdens. In groups A, B, and C, lung carcinoma, gastric adenocarcinoma, ovarian carcinoma, colorectal carcinoma, melanoma, and liver carcinoma exhibit averages of 40, 86, 72, 129, 170, and 76 mutations per sample, respectively. Meanwhile, within group F, less frequently cited tumors in articles are characterized by the lowest average mutation rates per sample, including renal carcinoma and myeloid leukemia, with 25 and 6 mutations per sample, respectively.

Tumors with a low mutation burden can pose challenges in identifying significant vaccine targets. Conversely, an extremely high mutation load results in a high load of neoepitopes, increasing the likelihood of identifying an immunogenic neoepitope that enhances cytotoxic activity against the tumor. Nevertheless, research indicates that the quest for neoepitopes remains feasible even in low-mutation-burden tumors [71].

The reviewed literature clearly shows an association between mutation burden and certain types of cancers, such as lung, colorectal, and breast cancer. It is important to highlight that, according to the 2023 report from the World Health Organization [72], these cancers are among the most common types worldwide, and their high prevalence rates mean that a larger patient population is available for research, increasing the statistical power of studies and the relevance of the findings. Additionally, lung, breast, and colorectal cancers are leading causes of cancer-related death, so research in these areas has the potential to significantly impact public health by reducing mortality.

Due to their high prevalence and mortality rates, these cancers often receive substantial research funding from government agencies, non-profit organizations, and private sector entities. There is also a well-established body of knowledge and research infrastructure for these cancers, including clinical trials, biobanks, and patient registries, making it easier to conduct and replicate studies. Furthermore, lung, breast, and colorectal cancers exhibit significant heterogeneity and have various subtypes, presenting opportunities to study different aspects of cancer biology, treatment responses, and resistance mechanisms.

In contrast, less well-studied tumors present a unique set of challenges and opportunities for research. These cancers face limited research funding, which can hinder extensive research and development efforts. Smaller patient populations also represent an issue, as fewer patients with these types of tumors make it difficult to conduct large-scale trials and achieve statistically significant results. Additionally, less common tumors can be highly heterogeneous, making it challenging to identify common therapeutic targets and treatment approaches. 

However, there are significant opportunities in researching less well-studied cancers. Addressing significant unmet medical needs, these efforts can provide hope and potentially life-saving treatments for patients with limited options. Furthermore, these tumors may offer unique biological insights that can contribute to the broader understanding of cancer mechanisms, leading to new discoveries that may also benefit more common cancers. The challenges mentioned above can drive innovative research approaches, such as the use of novel biomarkers, adaptive clinical trial designs, and advanced bioinformatics [73].

Another factor that directly impacts the effectiveness of computational neoepitope prediction is variant calling, which will be explored in a subsequent topic.

#### 3.2.2. Variant Calling

To predict patient-specific immunogenic neoepitopes, it is essential to identify which peptide sequences have undergone somatic mutations. Variant calling is the step where these mutations are identified prior to the neoepitope prediction.

**Figure 4:**
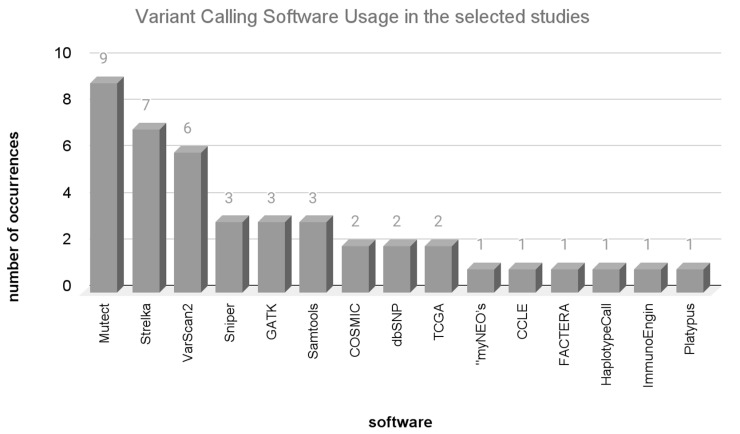
Variant calling software usage in the selected studies. The “x” axis represents the software or database, while the “y” axis represents the frequency of software utilization across the articles identified in this review.

The main challenge in this step is to distinguish common (germline) variants from a somatic tumor variant [74]. Programs such “Mutect” and “Samtools” are employed for sequence alignment and to define critical cutoff points (the quality of the sequencing at the position of a nucleotide and the comparison of germline and tumor sequences), which are essential for determining which mutations are truly somatic. Figure 4 presents the variant calling programs and how frequently they were used among the selected studies.

The methodologies employed in the articles for variant calling can be categorized into two primary forms: (a) variant calling programs that compare sequencing data from normal samples with those from tumor samples [20,21,22,23,24,26,29,30,31,32,33,34,35,36,37,38,39,40]; and (b) the retrieval of recognized and validated variants from databases [19,25,27,28,41].

According to the best practices for variant calling defined by Koboldt [74], the prediction results of these programs can be influenced by factors such as the proportion of pathologically mutated subclones and sequence depth. Sequencing depth represents a critical factor since the input data (normal and tumor sequencing data) are used to estimate the probability that the predicted variations are tumor somatic mutations. Subsequently, candidates for somatic variants undergo filtering and review to eliminate artifacts and germline variants.

The most frequently used variant calling programs were Mutect2, used in seven articles [20,23,29,31,35,36,37,40,42]; Strelka, used in six articles [22,23,24,26,32,36,38]; and GATK [31,34,39] and Samtools [22,30,33], used in 3 articles.

Mutect2 [75] is among the most extensively utilized mutation-calling programs, providing enhanced flexibility in accommodating data from various sequencing platforms. Additionally, it has the capability to predict a repertoire of somatic mutations even when only tumor sequencing data are available, while also excluding germline mutations identified in databases. However, this approach can result in a significant number of false positive predictions of mutation sites [74]. In a comparative study, Mutect2 demonstrated superior performance, especially when the mutation frequency was below 10% [76]. In contrast, Strelka showed better performance when the mutation frequency reached or exceeded 20%. Additionally, Strelka boasted a processing speed 34–39 times faster than Mutect2.

Several studies have utilized an approach to identify somatic mutations by querying databases that provide verified variants categorized as originating from either normal or tumor tissues. Platforms such as CCLE [77], COSMIC [78], dbSNP [79], and TCGA [11] offer a diverse range of tools and store data on known human variants.

TCGA and COSMIC store human variants, providing related data such as genes, clinical cases, and types of tumors. CCLE primarily stores data from tumor cell line models for use in other studies. dbSNP houses single-nucleotide variations, microsatellites, insertions, and deletions referenced in the literature. Some studies utilize this data to construct specific databases [19,25]. TCGA and dbSNP were utilized in studies related to personalized therapy [27,28,41].

When searching for variants in databases, it is important to highlight that numerous mutations generating potential neoepitopes might remain undetected. This is particularly relevant when studying tumors with limited data and/or less frequent HLA subtypes [80].

Additionally, it is worth mentioning that in this context, these databases can only confirm established mutations. Therefore, variant calling programs play a vital role in pinpointing novel and sample-specific mutations. They conduct statistical analyses that identify sites likely to harbor somatic mutations with substantial confidence, disregarding readings with numerous discrepancies or exceedingly low-quality scores due to their tendency to introduce more noise than signal [74].

Furthermore, it is also important to underscore that the selected studies integrated into this review investigated single-nucleotide polymorphisms (SNPs) mainly. These mutations, involving a single nucleotide base variation, represent the most prevalent mutations that can arise in tumor cells [81], which accounts for this outcome.

However, it is important to emphasize that some studies confirmed that insertion or deletion variations (INDELS) where one or more bases are inserted or removed are the second most abundant form of genetic variation in humans after SNPs (79% of the variants detected were SNPs and 21% of the variants were INDELs) [81,82]. It is evident that these deletions also have the potential to generate a neoepitope [83], and depending on the alteration to the genomic reading frame, insertions or deletions can lead to neoepitopes with multiple modified amino acids, entirely distinct from the wild-type form.

Several studies have utilized a combination of multiple programs rather than relying on a single one [22,23,24,31,34,35,36]. This approach of employing various programs in combination aims to reduce the occurrence of false negative variants [84]. Limitations associated with variant calling will be discussed in the next subtopic.

##### Limitations of Variant Calling

This section specifically addresses the guiding question “What are the strengths and limitations of the employed approaches?” by analyzing the limitations of the variant calling. Among these limitations, most are associated with the samples themselves, including those exhibiting low sequencing quality [33]. Factors such as contamination, low purity, and tumor heterogeneity can compromise the quality of the generated reads, along with meeting the necessary minimum coverage [83]. 

Tumor heterogeneity specially involves various subclones within a single tumor, each with unique spatial and mutational characteristics. This diversity influences the expression and immunogenicity of neoantigens. Different types and stages of tumors can exhibit high heterogeneity and diverse mutation profiles, impacting the effectiveness of variant calling [85].

Furthermore, the length of sequencing reads can introduce challenges and biases in genome assembly, particularly in repetitive regions, thereby impeding the identification of specific mutation types such as gene fusions, rearrangements, and inversions. While SNPs and INDELs are readily detectable in shorter reads, larger mutations may go unnoticed [86].

Moreover, the variant calling process itself can influence the selection of ineffective neoepitopes, depending on its execution [87]. Although INDEL-type mutations are less extensively studied, they also have the potential to yield immunogenic neoepitopes and have begun to be included in recent investigations. However, the identification of gene fusions remains challenging and necessitates specific tools like FACTERA [34]. Lastly, certain studies validate mutations using databases instead of sequence alignment, potentially overlooking significant tumor-specific and patient-specific mutations [19,25,27,28,41].

Advanced sequencing techniques like single-cell RNA sequencing (ScRNA-seq) provide detailed genomic data that reveal the clonal structure and mutation history of tumor subclones, helping in the identification of optimal neoantigens for targeted therapy. However, challenges such as cell isolation and viability, cost, and data integration issues remain hurdles to its widespread application [88].

In summary, the accuracy of variant calling hinges on factors such as sample quality, purity, and validation parameters, which persist as limiting factors in numerous studies.

### 3.3. Algorithms Employed for Prediction and Prioritization of Neoepitopes

In response to the guiding question “What algorithms and programs have been employed for predicting tumor neoepitopes?”, this section summarizes the results obtained regarding the algorithms and programs employed for predicting neoepitopes and examines the inherent limitations of current in silico methods and suggests potential strategies to overcome these challenges. First, we will present the programs for predicting epitopes with a binding affinity to MHC-I and MHC-II. The importance of the allele’s specificity will also be discussed. Next, the computational algorithms utilized by prediction programs will be summarized.

#### 3.3.1. Neoepitope Prediction Programs

**Figure 5:**
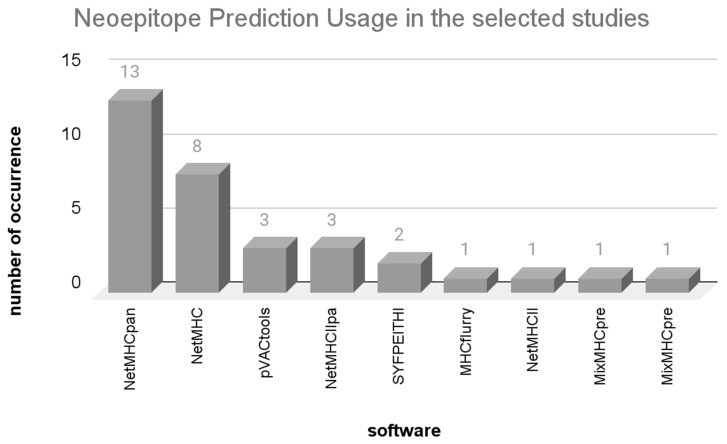
Neoepitope prediction usage in the selected studies. The horizontal (x) axis represents the neoepitope prediction programs, while the vertical (y) axis indicates the frequency of usage of each program across the articles.

After identifying genomic mutations within tumor cells, the functional consequences of these variants are assessed. This process leads to the creation of peptide sequences containing the mutations which are then used to predict neoepitopes. Subsequently, the second crucial step involves selecting or prioritizing those with greater immunogenic potential among them. Figure 5 showcases the neoepitope prediction programs identified in the reviewed studies. In response to the guiding question “What are the strengths and limitations of the employed approaches?”, Table 3 presents a list of all neoepitope predictors identified, with advantages and disadvantages.

As depicted, this review encompassed 49 studies, categorized into two sections (Table 1 and Table 2). The first segment (23 articles) included studies employing pre-existing neoepitope prediction programs already described in the literature. The subsequent segment (26 articles) included studies that developed novel neoepitope prediction programs.

The programs described in Figure 5 correspond to the initial segment of the review. These studies adopted an approach where NGS data were utilized for neoepitope prediction, employing established programs from the existing literature. The programs identified in the subsequent segment will be discussed in a forthcoming section.

The widely utilized programs encompassed NetMHCpan [19,20,23,24,25,26,30,32,34,36,37,40,41], closely followed by its counterpart NetMHC [22,27,28,29,32,33,34,38]. The pVAC-tools [22,29,35] program constitutes a package that provides supplementary tools alongside neoepitope prediction. Other programs such as NetMHC* (all variations of the program), pVAC-tools, MHCflurry [21], SYFPEITHI [32,38], and MixMHCpred [31] execute neoepitope prediction based on HLA-I binding affinity. Meanwhile, NetMHCIIpan [24,37,41], NetMHCII [34], and MixMHCpred2 [31] predict neoepitopes in relation to HLA-II binding affinity.

The second segment of the review, with 26 studies focusing on the development of new programs/algorithms, is outlined in Table 2. Among the programs identified in Table 2, 17 solely conduct neoepitope prediction with an affinity for HLA-I [42,44,46,47,48,49,50,52,53,56,57,58,59,63,64,65,66,67], 5 exclusively predict neoepitopes with an affinity for HLA-II [43,45,48,60,61], and 4 predict neoepitopes with an affinity for both HLA-I and HLA-II [51,54,55,62].

#### 3.3.2. HLA-I Restriction

In the context of personalized cancer therapy, the emphasis on predicting neoepitopes primarily revolves around the utilization and advancement of algorithms geared towards forecasting neoepitopes binding to HLA-I. This emphasis arises from the intracellular origin of tumor antigens. Such predictions are allele-specific, targeting a particular HLA allele. With over 25,000 distinct alleles of class I HLA genes (HLA-A, B, and C) cataloged [89], it underscores the importance of determining the specific HLA allele to aid in predicting potentially immunogenic epitopes.

**Figure 6:**
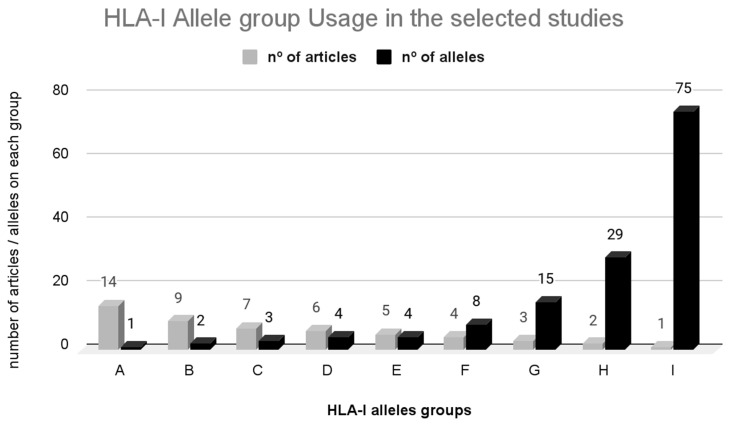
HLA-I allele group usage in the selected studies. The horizontal axis represents the group of alleles analyzed in the reviewed studies. The gray bars depict the count of articles utilizing alleles within the respective group, while the black bars indicate the number of alleles present in each group. (**A**) A*02:01; (**B**) A*01:01, A*03:01; (**C**) A*11:01, B*07:02, C*07:02; (**D**) A*24:02, B*08:01, B*27:05, B*35:01; (**E**) A*68:01, B*40:01, B*44:02, C*15:02; (**F**) A*26:01, B*15:01, B*18:01, B*44:03, B*51:01, C*01:02, C*02:02, C*04:01; (**G**) A*02:06, A*30:01, A*32:01, A*33:03, B*35:02, B*35:03, B*40:06, C*03:02, C*03:03, C*03:04, C*05:01, C*06:02, C*07:04, C*08:01, C*08:02; (**H**) A*02:02, A*02:05, A*23:01, A*25:01, A*29:01, A*29:02, A*30:02, A*31:01, A*33:01, A*02, B*07:05, B*13:02, B*14:01, B*15:18, B*37:01, B*38:01, B*39:01, B*45:01, B*52:01, B*54:01, B*55:01, B*55:02, B*58:01, C*07:01, C*12:02, C*12:03, C*14:02, C*15:05, C*16:01; (**I**) A*01:02, A*02:07, A*02:11, A*02:17, A*03:19, A*11:02, A*23:05, A*24:01, A*24:11, A*26:02, A*26:03, A*26:13, A*30:04, A*34:02, A*36:04, A*66:01, A*66:02, A*68:02, A*68:03, A*68:12, A*74:01, A*24, B*14:02, B*14:06, B*15:02, B*15:03, B*15:05, B*15:07, B*15:10, B*15:16, B*15:17, B*15:80, B*27:02, B*27:03, B*27:04, B*27:12, B*27:34, B*35:08, B*35:14, B*35:86, B*37:04, B*37:19, B*39:06, B*40:02, B*40:05, B*41:01, B*41:02, B*42:01, B*44:05, B*44:07, B*46:01, B*47:01, B*48:01, B*49:01, B*50:01, B*51:08, B*51:12, B*53:01, B*56:01, B*56:29, B*57:01, B*57:03, B*58:05, B*78:01, B*81:03, C*04:04, C*04:21, C*07:06, C*08:04, C*08:05, C*08:22, C*14:03, C*16:02, C*17:01, C*18:02.

The majority of epitope prediction programs utilize a 4-digit resolution of alleles, which denotes the level of detail in identifying HLA alleles (e.g., A*02:01, where “02” is the type and “01” is the subtype). In Figure 6, HLA-I alleles (A, B, C, D, E, F, G, H, I) were grouped according to the number of articles in which they were studied (in light gray) and the number of alleles in each group (in black). Each of the groups aggregated HLA-I alleles with the same frequency of use among the articles. The comprehensive list of alleles is provided in Supplementary Table S3.

For predicting neoepitopes associated with HLA-I alleles, Group A emerged as the most prevalent, utilized in 14 studies. This group includes the allele A*02:01, which is among the most widespread and extensively studied alleles worldwide [90]. Similarly, Group B and C encompass two and three alleles, respectively (B: A*03:01 and A*01:01; C: A*11:01, B*07:02, C*07:02), both highly prevalent with significant frequencies across continents. The alleles in Groups D, E, F, G, and H (featured in six, five, four, three, and two articles, respectively) also represent some of the most common alleles globally. In Group I, the alleles were less frequently used in studies (one article). Notably, this group also includes rarer alleles with heightened frequencies within the population [91].

Certain alleles demonstrate distinct distribution patterns across various regions. Notably, allele C*15:02 is more prevalent in Australia, while B*40:06 displays a higher frequency in South Asia. Additionally, A*31:01 is commonly found in Central and South America, B*35:08 and B*38:01 exhibit elevated frequencies in West Asia, and B*55:02, A*74:01, B*53:01, and C*16:01 are prevalent in Sub-Saharan Africa. Alleles A*02:07 and B*46:01 are more frequent in Southeast Asia, B*50:01 is prominent in North Africa, and B*56:01 prevails in Northeast Asia and Australia [91].

The precise identification of the HLA allele is of significant importance in neoepitope prediction. However, the distribution of these alleles is not uniform within the population, which affects algorithm training. Rarer alleles have limited available data in the existing literature, also posing challenges in algorithm training. Consequently, the prediction accuracy for these rarer alleles tends to be lower, resulting in an elevated false positive rate [92].

In summary, since the selection of major histocompatibility complex (MHC) alleles is crucial for neoepitope prediction due to their essential role in presenting peptide fragments to T cells, the rationales cited in the reviewed articles for selecting specific MHC alleles focus on several factors.

These include allele frequency and ethnicity-specific alleles, with studies commonly referencing the Allele Frequency Net Database [91] to choose alleles prevalent in specific populations. Binding affinity is also important, as alleles that bind a wide range of peptides with high affinity are preferred for presenting a diverse set of neoepitopes. Clinical relevance plays a role as well; certain MHC alleles are associated with specific diseases or conditions, and selecting these alleles can aid in understanding and targeting disease-specific neoepitopes. The availability of comprehensive sequence data for certain alleles can also facilitate more accurate neoepitope prediction. Finally, prior research influences allele selection, and well-studied alleles documented in the literature are often chosen due to the existing body of knowledge and available comparative data.

The articles discussing neoepitope prediction with HLA-I affinity primarily focused on processing peptides with a length of 8 to 11 amino acids. Specific studies adhered to a 9-amino-acid size [26,27,28,29,33], while others slightly expanded the prediction scope to encompass 7–11 amino acids [35] or 8–15 amino acids [39]. In these articles discussing neoepitope prediction to HLA-I affinity, emphasis was placed on the analysis of 9-amino-acid peptides due to their optimal binding stability with the MHC-I molecule [93].

#### 3.3.3. HLA-II Restriction

**Figure 7:**
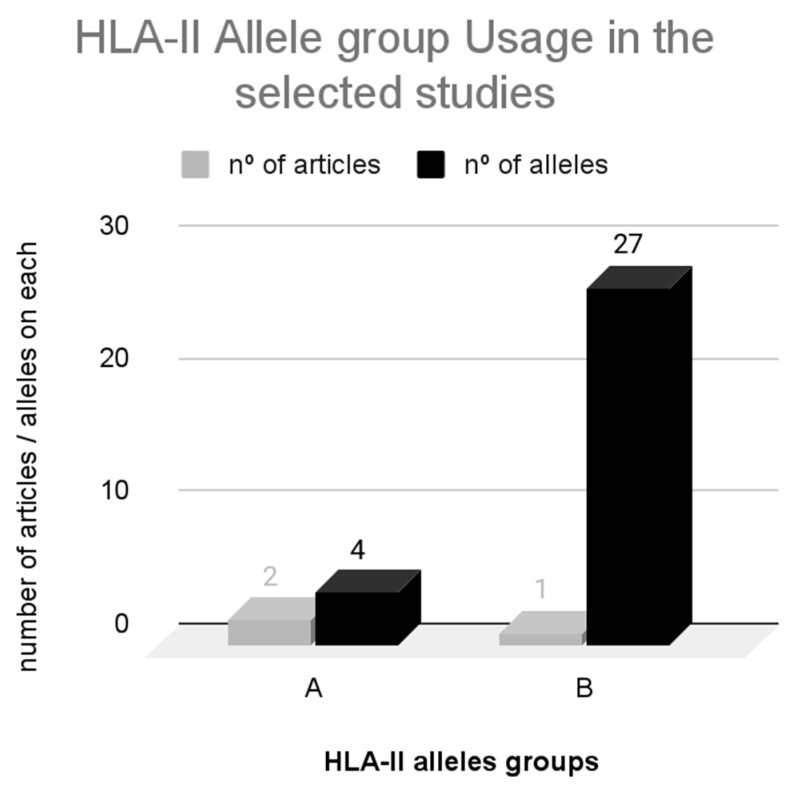
HLA-II allele group usage in the selected studies. The horizontal axis represents the allele group analyzed in the reviewed studies. The gray bars depict the count of articles employing alleles within the respective group, while the black bars indicate the number of alleles present in each group. (**A**) DQB1*03:01, DQB1*06:03, DRB1*12:01, DRB1*13:01; (**B**) DPB1*03:01, DPB1*04:01, DQB1*02:01, DQB1*02:02, DQB1*03:02, DQB1*03:03, DQB1*04:01, DQB1*04:02, DQB1*05:01, DQB1*06:01, DQB1*06:02, DQB1*11:01, DQB1*15:01, DRB1*01, DRB1*01:02, DRB1*03:01, DRB1*04:04, DRB1*04:05, DRB1*04:06, DRB1*07:01, DRB1*08:02, DRB1*08:03, DRB1*09:01, DRB1*11:01, DRB1*12:02, DRB1*15:01, DRB1*15:02.

In Figure 7, HLA-II alleles (A, B) were grouped according to the number of articles in which they were studied (in light gray) and the number of alleles in each group (in black). Each of the groups aggregated HLA-II alleles with the same frequency of use among the articles. The comprehensive list of alleles is provided in Supplementary Table S4.

The prediction of neoepitopes restricted by HLA-II is less extensively studied compared to HLA-I. In this context, the A allele group was employed in two articles, while the B allele group was utilized in one article. The majority of alleles from the B group were featured in a single article [34], which encompassed various distinct samples.

HLA class II molecules encompass three loci (HLA-DR, HLA-DP, and HLA-DQ) and exhibit affinities with epitopes spanning 15 to 24 amino acids, as highlighted in the works of GOSH (2019) [15] and WANG (2010) [16]. These characteristics were investigated in three recent articles [34,35,41] that underline the significance of identifying neoepitopes with an affinity for MHC class II, yet this aspect remains relatively underexplored in the context of therapeutic vaccine development. Predicting MHC-II-restricted neoepitopes poses challenges due to the limited understanding of the class II antigen processing and presentation pathway. Additionally, it is observed that class II epitopes tend to be larger and more versatile [94].

Limitations associated with HLA specificity will be discussed in a subsequent section.

#### 3.3.4. Prediction Algorithms for Neoepitopes

The discovery of peptide sequences bound to HLA molecules in the early 1990s sparked investigations into the sequence patterns responsible for allele-specific peptide–HLA binding. Given the complexities of the binding mechanism, various algorithms and software rooted in pattern recognition and machine learning principles were subsequently developed [95,96,97].

The latest and crucial algorithms for neoepitope prediction highlighted in this review predominantly employ neural networks. Neural networks represent a sophisticated learning approach capable of capturing the nonlinearity inherent in the binding process, along with the interrelation among distinct amino acid binding positions, ultimately resulting in enhanced performance [96].

Supplementary Table S5 presents the algorithms and matrices identified within the set of neoepitope predictors. Here, in response to the guiding question “How are the predictions validated?”, Table 4 presents the strengths and limitations of the neoepitope prediction algorithms.

Among the 23 studies covered in the initial section of this review, the SYFPEITHI predictor stands out as the earliest one, with its algorithm coded in Object Pascal, an object-oriented programming language [96]. This program remains the sole user of this particular object-oriented programming language. In contrast, the other programs identified (NetMHC, NetMHCpan, NetMHCII, NetMHCIIpan, pVACtools, and MHCflurry) were coded in the Python programming language and employ neural networks as their predictive methodology. Lastly, MixMHCpred and MixMHC2pred were coded in the C++ programming language.

The programs NetMHC, NetMHCpan, NetMHCII, and NetMHCIIpan are trained using data from in vitro peptide binding affinity tests. These programs are designed to be allele-specific, targeting the prevalent HLA alleles in the human population that have shown a connection with immunogenicity. The “pan” versions of these tools are particularly suited for analyzing cancer-related data. These versions incorporate mass spectrometry data, leading to a reduction in false positive results, and they encompass training data derived from tumor origins. Furthermore, the “pan” version enables predictions for HLA alleles that have been less extensively studied [57].

The pVAC-tools program [22,29,35,64] offers a versatile toolkit that seamlessly integrates with other prominent tools for detecting neoepitopes. It not only works well with programs like NetMHCpan and MHCflurry but also provides the unique advantage of directly incorporating data through the processing of variant calling information (including SNPs, indels, and gene fusions). This capability enables pVAC-tools to perform neoepitope prediction and prioritization while considering factors such as gene expression and homology analysis.

The MHCflurry program [21,52] demonstrates significant improvements in both processing speed and training. It integrates tumor epitopes detected through mass spectrometry, thereby enhancing accuracy and minimizing the risk of missing potential candidates. While the programs MixMHCpred and MixMHCpred2 [31] do not predict neoepitopes, they are capable of evaluating various peptide scores and ranking the most probable HLA-I ligands. Trained on naturally occurring peptides, these programs provide a score rather than a predicted affinity value.

In the latter section of the review, among the 26 articles detailing the development of new programs, neural networks emerged as the method most frequently utilized [43,44,45,46,47,48,49,52,53,56,57,58,60,61,62,63,64,66,67]. Some programs implement modified versions of neural networks, such as convolutional neural networks [43,46,47,49]. Using this variation of the algorithm has led to enhanced performance in neoepitope prediction programs, particularly regarding HLA-II affinity, as this method is capable of modeling the nonlinearity of peptides [26].

For predicting neoepitopes that account for both HLA-I and -II affinity, the programs Ivax [51] and Ancer [55] employ the EpiMatrix algorithm [98]. The strength of this algorithm lies in its ability to explore HLA families that share common characteristics, thereby reducing redundancy in epitope mapping and addressing HLA polymorphisms, both utilizing six HLA-I alleles and eight HLA-II alleles for neoepitope prediction. In the case of Ivax [51], this program uses overlapping 9-mer frames of amino acids, whereas Ancer [55] employs 9- and 10-mer frames of amino acids. Additionally, these same programs utilize JanusMatrix to evaluate the homology of peptide sequences.

Moreover, in the context of predicting neoepitopes for both HLA classes, the program MS2Rescore [54] leverages the XGBoost [99] algorithm for its efficiency and rapid processing. MS2Rescore stands out from other neoepitope predictors by integrating newly trained immunopeptidome MS2PIP models, DeepLC, and Percolator into a single software package. This integration significantly enhances neoepitope identification rates and specificity. Additionally, the program ProTECT [62] predicts neoepitopes using the NetMHC programs. It distinguishes itself in the realm of neoepitope prioritization by seamlessly integrating variant calling data and peptide sequence homology with the rankboost technique [100].

Programs that predict neoepitopes with only HLA-I affinity primarily utilize neural networks. ACME [44] predicts peptide transport and cleavage and evaluates T cell response using the BLOSUM50 matrix [101]. ACME offers significant improvements over other methods, boasting an approximately 5 percentage point increase in the Spearman correlation coefficient (SRCC). Furthermore, ACME outperforms the NetMHCpan 3.0 model on several metrics such as PCC and AUROC, particularly for peptides of 9, 10, and 11 amino acids. ACME can also predict new alleles not available in the training data. INeo-Epp [50] prioritizes neoepitopes by evaluating their physicochemical characteristics using random forest and Boruta algorithms, which help to eliminate false positive neoantigens, significantly reducing the number of candidates requiring experimental verification. MHCSeqNet [53] also employs neural networks for peptide transport and cleavage prediction. It is flexible, allowing predictions for peptides of any length and for any MHC class I allele with a known amino acid sequence. MHCSeqNet outperforms tools like NetMHCPan and MHCflurry in terms of performance. OnionMHC [58] differentiates itself by incorporating both structure- and sequence-based features to predict the binding affinity of peptides with the HLA-I allele A*02:01. While models such as NetMHCpan4.0 rely solely on peptide sequences, OnionMHC combines information from both areas, resulting in improved performance. SHERPA [59] evaluates physicochemical characteristics with the BLOSUM62 matrix, increasing the selectivity of immunogenic neoepitopes. PTuneos [63] integrates variant calling data, uses the NetMHCpan program for neoepitope prediction, and employs random forest to assess T cell activation. Seq2Neo [66] integrates variant calling data, epitope prediction with convolutional neural networks, T cell activation, and gene expression.

Other programs employ various algorithms for neoepitope prediction. 3pHLA [42] uses the CART and random forest algorithms [102] for direct neoepitope prediction, integrated with structural analysis. These methods can handle binary and multi-class classification problems and are widely used in various biological applications. PLATO [65] uses Bootstrap [103] and random forest algorithms, integrating neoepitope prediction with peptide transport and cleavage, variant calling integration, gene expression, and a T cell activation score. Neopepsee [56] uses neural networks for neoepitope prediction and prioritizes using six physicochemical characteristics, gene expression, homology, peptide transport, and cleavage, employing naïve Bayes, random forest, and support vector machine algorithms.

For programs predicting neoepitopes with HLA-II affinity [43,45,48,60,61], neural networks are also the predominant method. FIONA [43] utilizes convolutional neural networks. The program developed by Mettu and collaborators [45] employs NetMHCII and prioritizes neoepitopes with a score that assesses binding stability. ITcell not only predicts neoepitopes using neural networks but also evaluates peptide transport and cleavage and creates a neoepitope model to assess T cell recognition. MARIA [61] uses neural networks and also accesses gene expression.

Optimizing these algorithms involves incorporating additional variables into epitope prediction to account for antigen processing and presentation pathways, thereby increasing the accuracy of predictions. However, studies still exhibit a high rate of false positives in both in vitro and in vivo tests, estimated to be around 50% to 95% [3]. This indicates that not all predicted epitopes presented by MHC-I on the surface of tumor cells and/or APCs will induce effective CD8+ T cell responses, necessitating further methods of neoepitope prioritization.

#### 3.3.5. Limitations in MHC-I Neoepitope Prediction

To address the guiding question “What are the strengths and limitations of the employed approaches?”, we present the limitations identified in the MHC-I neoepitope prediction in the subsequent discussion. Machine learning stands as the most commonly utilized and well-established method for predicting neoepitopes when applied to epitope prediction in sequences derived from microbial samples. However, when addressing tumor-origin samples, a key challenge arises concerning the composition of the database used to train the algorithm [104]. Given that the method relies on sequence similarity and pattern recognition, it is imperative that algorithm training incorporates tumor-origin data. Yet, there remains a scarcity of data for constructing these databases, thereby diminishing the predictor’s performance. In terms of training, another issue is allele-specific prediction. Common and extensively studied HLA alleles, such as HLA-A2, benefit from ample training data. Conversely, for rarer alleles, data availability is limited, significantly reducing reliability in these cases [90].

These limitations are evident in the reviewed programs that heavily rely on microbial data for algorithm training. Many studies confine their analyses to the HLA alleles identified in the NGS data utilized. This necessitates caution when extrapolating results to other alleles. Programs like OnionMHC [58] validated their results for only one allele (A*02:01), potentially displaying varied performances for other alleles. Thus far, most algorithms have been trained with data obtained by testing predicted epitopes. In other words, the database is confined to data acquired from in silico prediction, disregarding other potential immunogenic epitopes. One approach to circumvent this is to incorporate data generated by mass spectrometry, which directly identifies sequences presented on the cell plasma membrane. However, this approach was scarcely explored in the review [49,52,57,65,67], mainly due to its inherent difficulties, including sample quality dependency and low yield. Nonetheless, mass spectrometry has been pivotal in understanding physicochemical properties in epitope–MHC binding and holds the potential to identify rare sequences overlooked by standard techniques. It is important to emphasize that the inclusion of this data in algorithm training has enhanced our understanding of antigen processing and presentation [105].

The majority of neoepitope prediction programs solely consider the binding affinity of the peptide to class I MHC molecules as the metric to evaluate immunogenicity [105]. However, it is known that numerous biological factors, many of which are still unknown, can influence or determine the immunogenicity of these sequences. Cleavage, transport, expression level, and recognition by T cells are among the factors that notably influence peptide immunogenicity. Additionally, other factors related to structural analysis are beginning to be explored, such as the physicochemical characteristics of sequences influencing T cell activation and the affinity of epitopes to class II MHC molecules [106].

#### 3.3.6. Limitations in MHC-II Neoepitope Prediction

In line with the previous discussion, we provide in the subsequent analysis the limitations identified in the MHC-II neoepitope prediction to answer the guiding question “What are the strengths and limitations of the employed approaches?”. CD4+ T cells play a crucial role in generating specific and long-lasting cellular immunity. Within the tumor microenvironment, some studies [107] have demonstrated that the activation of CD4+ T cells can impact the immune response against tumors. This response can occur even in instances where tumor cells negatively regulate class I MHC expression. Consequently, precise identification of class II neoepitopes can prove critical for optimizing neoepitope prediction and devising therapeutic cancer vaccines.

Predicting epitopes with class II MHC affinity has been relatively underexplored in the reviewed studies and presents additional challenges. These epitopes tend to be longer and more variable in length than class I MHC peptides, spanning from 15 to 24 amino acids. Moreover, besides exhibiting more promiscuous peptide binding, unstable regions contribute to epitope non-specificity, complicating the identification of immunogenic epitopes, particularly in terms of pattern recognition by the utilized algorithms. When these molecular factors are combined with the lack of validation for these epitopes as tumor neoepitopes, algorithm training becomes challenging, resulting in diminished performance [107].

Similar to predicting neoepitopes binding to class I MHC, the utilization of mass spectrometry data for class II MHC affinity epitopes has facilitated the development of more reliable binding prediction programs, as evidenced by NetMHCIIpan [108].

Peptide immunogenicity is influenced by various factors, including those related to structural analysis. These factors are beginning to be explored, such as the physicochemical characteristics of sequences influencing T cell activation and the affinity of epitopes to class II MHC molecules [106].

#### 3.3.7. Prioritization Methods of Immunogenic Neoepitopes

The programs identified in this review can be classified into two categories: those exclusively focused on neoepitope prediction [43,45,46,47,49,52,54,57,58] and those that, in addition to neoepitope prediction, integrate various prioritization methods to reduce false positives [42,44,48,50,51,53,55,56,59,60,61,62,63,64,65,66,67]. Neoepitope prioritization methods aim to evaluate other crucial parameters for generating an efficient immune response. While predicting neoepitopes with HLA affinity is a pivotal step, it alone is insufficient for accurately selecting immunogenic sequences [109].

One of the most important prioritization methods is the assessment of gene expression [56,59,61,63,65,66,67], as a selected neoepitope may be contained within a non-expressed gene. It is important to emphasize that there is no consensus on the optimal gene expression cutoff point. Moreover, even if a neoepitope is overexpressed, it may not trigger an effective immune response, as this relies on various other factors.

Another prioritization method explored by some programs [42,50,56,60] is structural analysis, which evaluates intrinsic physicochemical characteristics of the neoepitope, such as polarity, amino acid position, pair interactions, and entropy. This prioritization process seeks to assess which amino acid sequence parameters determine or influence T cell recognition and activation.

Additional prioritization parameters commonly used include peptide transport and cleavage analysis, and homology assessment. Peptide transport and cleavage are important for antigen presentation processing pathways, but some studies suggest that analyzing this separately has a limited impact on the results [110]. This is because current epitope predictors, such as NetMHCpan and MHCflurry, take into account processed sequences (transported and cleaved). Homology searches are performed between selected neoepitopes and naturally expressed peptide sequences in the genome. Neoepitopes highly similar to natural epitopes increase the risk of cross-reactivity in vaccine formulation.

Since the programs identified in this review use machine learning for neoepitope prediction, an important factor to consider is the data used for algorithm training.

### 3.4. Training Datasets Models

To answer the guiding question “What dataset is used as a training model?”, we delve into the findings concerning the training of algorithms outlined in the review, aiming to address the datasets utilized in model training.

Training algorithms for epitope prediction entail the use of experimentally validated peptide sequence data. Typically, these datasets are sourced from microbes, a trend observed across all reviewed programs except for SHERPA [59], PLATO [65], and SIGNEO [67], which were exclusively trained using tumor data. Other programs opted for a blend of microbiome and tumor data [42,43,44,46,47,48,49,50,52,53,54,55,56,57,58,62,63,64,66].

Incorporating tumor-origin data can aid in reducing the false positive rate. Additionally, employing data obtained from mass spectrometry analysis can further mitigate this rate. Research has demonstrated that including mass spectrometry data in the training set is crucial for developing a high-performance predictor [109]. Without this data, both the size of the training set and allele coverage are significantly diminished. Moreover, achieving a balance in the training set, combining mass spectrometry data with peptide–MHC class I layer depth, is imperative, as mass spectrometry data alone do not accurately represent antigen processing [111].

Despite these advancements, in silico methods, have limitations, spanning from biases in sequencing technologies and variant calling to issues in the epitope prediction algorithm that fail to consider other factors influencing immunogenicity. Notably, there is a lack of exploration into neoepitopes binding to class II MHC molecules.

### 3.5. Performance and In Silico Validation

The guiding question “How are the predictions validated?” is tackled in the following paragraphs, where we delve into the in silico validation of predicted neoepitopes and present the findings regarding the performance evaluation of the algorithms and programs. In silico analyses, in addition to accelerating the identification of potential immunogenic neoepitopes, are important for identifying key points and genetic mechanisms that trigger an immune response against the tumor. Across the studies reviewed in this manuscript, the methodologies employed to assess performance measures displayed some degree of variability. Typically, the aim is to evaluate the likelihood of a predicted peptide sequence being a true neoepitope with affinity for an HLA allele, as well as to determine if this neoepitope genuinely elicits an immune response. However, a recurring shortcoming in this domain is the lack of validation of the acquired data [112].

Many studies showcase predictor performance using performance metrics, often quantified by the AUC (area under the curve). This metric is commonly utilized for evaluating classification models, particularly in binary classification scenarios where the goal is to distinguish between two classes, such as “positive” and “negative”. Within a receiver operating characteristic (ROC) curve—a standard tool for assessing binary classification models—the AUC represents the area under the curve. This curve plots the true positive rate (sensitivity) against the false positive rate (1-specificity) at various classification thresholds. The AUC value serves to quantify the overall ability of the model to differentiate between the two classes. A higher AUC generally signifies a better-performing model, with an AUC of 1 representing a perfect classifier, and an AUC of 0.5 representing a random classifier. Neoepitope predictors use this metric to define the performance of the algorithm when subjected to a test group after training.

The predictors identified in this review exhibited AUC values spanning from 0.65 [55] to 0.99 [53]. However, each predictor possesses unique characteristics, such as the algorithm employed, biological traits covered, HLA alleles utilized for training, and the composition of the training data. Consequently, depending on the object of study, the predictor with the highest AUC value may not necessarily be the optimal predictor of immunogenic neoepitopes. This is due to the specific nuances of each predictor, such as the training dataset and the parameters defined in the program.

ACME [44] demonstrated an AUC value of 0.9 for HLA-A and 0.88 for HLA-B, shedding light on performance discrepancies among alleles. However, no performance breakdown is available for HLA-C alleles within the same study. Additionally, the study revealed variations in predictions concerning neoepitopes of different sizes, with 9 amino acids (9AA) exhibiting the most favorable performance.

MHCSeqNet [53] exhibited an impressive AUC performance of 0.99 for alleles prevalent in the population and 0.79 for what are termed “invisible” alleles. This suggests that the predictor possesses robust predictive capabilities for epitopes binding to any patient’s HLA allele, a highly desirable trait given that neoepitopes capable of binding to multiple HLA alleles are generally more likely to be immunogenic.

MHCflurry [52] achieved an AUC performance of 0.8 and highlighted its superior performance when dealing with peptide sequences longer than nine amino acids compared to other tools. This distinction may be influenced by the algorithm’s training data, which amalgamates microbiome and tumor data alongside mass spectrometry data.

The predictor 3pHLA [42] also demonstrated outstanding performance, attaining a precision rate of 0.99, validated across 28 HLA-I alleles.

A significant leap in predicting neoepitopes with HLA-II affinity comes from the FIONA predictor [43], boasting an AUC value of 0.94. Utilizing convolutional neural networks and integrating tumor data into its training algorithm, FIONA achieves superior performance compared to other predictors like MARIA [61] (0.87) and NetMHCIIpan (0.76).

It is worth noting that the majority of predictors primarily assess the binding of the peptide sequence to the HLA molecule, without considering the subsequent recognition and activation of T cells. Currently, this gap has been explored by numerous studies, predominantly through the structural analysis and physicochemical characterization of neoepitopes.

INeo-Epp [50], while not making direct predictions of neoepitopes, prioritizes immunogenic neoepitopes based on physicochemical characteristics, exhibiting an AUC performance of 0.78. Conversely, Neopepse [56] directly predicts neoepitopes and conducts supplementary analyses encompassing physicochemical traits, such as variant calling data, homology searches, gene expression, transport, and cleavage, achieving an AUC performance exceeding 0.9.

OnionMHC [58] achieves an AUC performance of 0.83 and utilizes molecular modeling to prioritize neoepitopes. However, its predictions are limited to a single allele and a specific neoepitope size.

These studies highlight the varied factors that differentiate neoepitope prediction programs and algorithms. The choice of which one to utilize should consider the nature of the data under examination. Depending on variables such as type of tumor, sequencing data quality, patient HLA alleles, and the predictor’s approach to these factors, certain programs may prove more suitable than others.

Programs like Ivax [51], Neopepsee [56], ProTECT [62], and pVACtools [64] boast expansive scopes, encompassing numerous critical parameters within the neoepitope prediction process. They directly integrate variant calling data, predict neoepitopes for both HLA-I and -II, employ prioritization techniques such as gene expression analysis, consider aspects such as transport and cleavage, assess physicochemical properties, and explore homology.

A discernible trend emerges in recent studies spanning from 2020 to 2022 [43,45,47,49,58,66], where programs developed during this period tend to incorporate convolutional neural networks, primarily due to their exceptional performance.

### 3.6. Can This Prediction Accurately Guide the Identification of Potential Targets for the Development of Therapeutic Vaccines?

To answer the guiding questions “What are the strengths and limitations of the employed approaches?” and “Can this prediction accurately guide the identification of potential targets for the development of therapeutic vaccines?”, we emphasize the importance of experimental validation and present the conduct of some clinical tests. In studies implementing neoepitope identification protocols using NGS data, the validation of predicted neoepitopes involves in vitro and in vivo tests that identify cytokines such as IFN-γ (interferon-gamma) and TNF-α (tumor necrosis factor-alpha), which indicate T cell activation. In this review, 55% of the selected studies performed an in vitro or in vivo validation of the predicted neoepitopes [21,22,23,24,26,27,28,30,31,32,33,34,36,37,38,39,40,41,44,45,51,58,59,60,61,63,66]. However, 45% did not perform such validation assays [19,20,25,29,35,42,43,46,47,48,49,50,52,53,54,55,56,57,62,64,65,67]. Instead, these studies conducted an in silico validation using known neoepitopes. Among these, some studies focused on developing new tools for predicting neoepitopes, evaluating the program’s performance in classifying known neoepitopes, but not its effectiveness with previously unknown peptides.

Five studies conducted clinical tests [34,36,38,40,41]. In one study [34], immunogenic peptides were identified and used for the vaccine design. The results observed in patients receiving personalized neoantigen therapy included a complete and durable response beyond 29 months in a patient with metastatic thymoma. A patient with metastatic pancreatic cancer showed a partial response related to the immune system. The other four patients achieved prolonged disease stabilization, with a median progression-free survival of 8.6 months.

In a separate study [36], despite generating specific immune responses against both systemic and intratumoral neoantigens after vaccination, all patients experienced tumor recurrence and died of progressive disease. This indicates that the T cell responses elicited still need to overcome considerable challenges to produce clinically relevant antitumor activity, including tumor-intrinsic damage and immunosuppressive factors in the microenvironment. Low MHC class I expression in the tumor and lack of class II expression may hinder tumor recognition by neoantigen-specific T cells. 

In the study reported in [38], 41 months after the start of vaccination, the radiological imaging results (February 2016) showed no evidence of tumor recurrence. In yet another study [40], approximately 55% (61 sequences) of the predicted neoepitopes were confirmed to bind to HLA-I molecules, with three of them eliciting an IFN-γ response. The administered vaccine led to tumor remission alongside bone marrow transplantation.

Finally, in [41], a neoantigen vaccine developed against melanoma has demonstrated that neoantigen vaccination, in combination with checkpoint blockade, can significantly enhance vaccine-induced immune responses and lead to lasting clinical effects in some patients.

Nonetheless, numerous challenges persist within the pipelines established by the scientific community. The lack of standardization in computational methods, applied parameters, utilized datasets, and even the presentation of results complicates the formulation of a comprehensive gold standard protocol, particularly given the genomic diversity of tumors and human alleles, alongside the incomplete understanding of biochemical mechanisms mediating the immune response. Furthermore, many studies do not validate their predictions through in vivo or in vitro methods, despite the importance of this validation given that the error rate for predictions can be as high as 95%. It is also crucial to note that reports of cytokine production in response to neoepitopes in these tests do not always translate to effective tumor inhibition or reduction. Therefore, in addition to considering the specific alleles and types of tumors studied, it is essential to evaluate the combination of identified neoepitopes with adjuvants and conventional chemotherapy drugs for effective treatment.

Despite these limitations, based on the results extracted from in vitro, in vivo, and some clinical tests in humans in the selected studies for this review and the preceding discussions, the prediction of immunogenic neoepitopes appears promising, offering robust evidence for the development of therapeutic vaccines against cancer. 

### 3.7. Restrictions Regarding the Article Selection Process

Regarding the constraints of this scoping review, firstly, we have solely included articles written in English and publicly accessible. Second, it is conceivable that certain programs and algorithms for neoepitope prediction remain proprietary, rendering both their code and reference articles inaccessible to the public.

Additionally, articles focusing solely on specific genes have been excluded. The methodologies employed in these studies vary considerably based on their objectives, gene types, and available datasets. The divergence in the target-specific approach adopted by those studies distinguishes them from our scope of focus, leading to their exclusion.

Furthermore, given that our review focuses on computational approaches applied to human data for personalized therapy, articles that did not utilize human high-throughput sequencing data were not incorporated.

## 4. Conclusions and Perspectives

Here, we provide an overview of the approaches for identifying immunogenic neoepitopes and future directions in neoepitope prediction research. This includes advancements in computational models and the need for cross-disciplinary collaboration. 

Personalized cancer therapy has emerged as a burgeoning field of research, focusing on understanding the biological mechanisms underlying the immune response to disease. This field integrates computational approaches aimed at developing neoepitope predictors and cancer vaccines. Given the intricate nature of cancer and the limitations inherent in specific in silico methods in addressing this complexity, studies have ventured into exploring diverse parameters and data integration techniques to refine the selection of cancer vaccine targets.

The design of therapeutic vaccines has been facilitated by a deeper understanding of the diversity of tumor-associated neoantigens, the native immune response, and the advancements in genomic sequencing technologies. Consequently, therapeutic cancer vaccines hold the potential to induce tumor regression and eradicate minimal residual disease.

The focus of this scoping review was guided by primary investigations aiming to define the most commonly used computational methods and approaches currently available for predicting tumor neoepitopes, crucial for the development of therapeutic vaccines. We assess the application of neoepitope prediction algorithms, pivotal steps in in silico approaches facilitating the selection of potential immunogenic tumor targets, algorithm performance, and associated limitations spanning from 2012 to 2024. To structure this review, we followed seven guiding questions: “What are the critical steps in identifying immunogenic neoepitopes?”; “What are the main types of tumors studied?”; “What algorithms and programs have been employed for predicting tumor neoepitopes?”; “What dataset is used as a training model?”; “How are the predictions validated?”; “What are the strengths and limitations of the employed approaches?”; and “Can this prediction accurately guide the identification of potential targets for the development of therapeutic vaccines?”, aiming to answer them using data obtained from the selected studies.

The critical steps identified in this review for selecting immunogenic neoepitopes (variant calling, the prediction of neoepitopes and prioritization, and validation) represent the primary approach for identifying immunogenic neoepitopes in next-generation sequencing data. The first step, variant calling, plays a crucial role in facilitating the selection of patient-specific tumor mutations. Programs that compare normal and tumor sequences emerge as the optimal strategy at this stage, enhancing the likelihood of identifying novel mutations and tumor-specific mutations and evaluating parameters such as tumor purity. Mutect stands out as the most commonly utilized tool in this category; however, as evidenced, the choice of program may vary depending on the dataset, with some outperforming others. An essential consideration at this stage is the tumor origin of the sequencing data, as it significantly influences mutation rates and purity. Limitations associated with this stage center around the quality of sequenced samples, encompassing the sample collection process and the choice of the sequencing approach.

Challenges such as errors in identifying true mutations (false positives/negatives), tumor heterogeneity, and sequence depth can be addressed through several strategies. Enhanced next-generation sequencing (NGS) platforms offering improved accuracy and coverage can reduce errors. Additionally, advanced bioinformatics pipelines integrating algorithms and machine learning techniques can more effectively distinguish genuine variants from sequencing artifacts. Multi-sample analysis, which integrates multiple regions of both tumor and normal tissue, allows for a comprehensive assessment of mutation spectra and accommodates tumor heterogeneity. Finally, employing cross-validation techniques such as digital droplet PCR or Sanger sequencing can validate key variants identified by NGS, ensuring their accuracy and reliability.

The second and third critical steps, neoepitope prediction and prioritization, respectively, are directly interconnected and represent the stages that receive the most emphasis in the development of novel algorithms and in silico approaches aimed at enhancing the performance of these predictors. 

In the context of neoepitope prediction, despite the diversity of machine learning algorithms available, neural networks, and more recently, convolutional neural networks, dominate as the preferred choices for neoepitope prediction. These algorithms offer advantages such as the ability to discern intricate patterns, high accuracy compared to other methods, scalability, adaptability, processing speed, and a reduced rate of false positives. Consequently, they hold the potential to deepen our understanding of the immune response and identify novel targets for immunotherapy.

In the context of neoepitope prioritization, which involves identifying and selecting the most promising neoepitopes, the critical steps include ranking neoepitopes based on their predicted binding affinity and immunogenicity, prioritizing neoepitopes unique to the tumor and not present in normal tissues, and considering the mutational load and expression levels of the mutant protein in the tumor. These approaches could be enhanced with the development of more accurate prediction algorithms that incorporate machine learning and deep learning techniques to improve binding affinity and immunogenicity predictions. Integrating sequencing data with proteomics and transcriptomics data to validate the expression of neoepitopes, along with using multiple prediction tools to provide a consensus prediction for better reliability, would also help enhance performance. Additionally, high-throughput screening techniques such as peptide–MHC tetramer assays and in vitro T cell activation assays can be employed to experimentally validate predicted neoepitopes. Using patient-specific HLA typing and tumor heterogeneity data to tailor predictions to individual patients, incorporating structural modeling to better understand binding interactions of neoepitopes/HLA molecules, and applying network-based approaches to study the neoepitope landscape to identify key immunogenic hotspots within the tumor would further improve the effectiveness of these approaches.

In brief, future directions should focus on expanding HLA databases to include diverse and rare HLA alleles, enhancing the predictive power for all populations. Employing machine learning models trained on large, diverse datasets can improve the prediction of peptide–HLA binding affinities and immunogenicity. Additionally, developing integrative models that consider multiple factors, such as proteasomal cleavage, TAP transport, peptide–MHC stability, and T cell receptor affinity, will further refine predictions. Utilizing high-throughput experimental techniques like mass spectrometry to validate and refine computational predictions can create feedback loops for continuous model improvement.

The fourth critical step, in vitro and in vivo validation of predicted neoepitopes, highlights additional issues such as a high rate of false positives, indicating that the ability to induce the production of cytokines does not guarantee that there will be an immune response against the tumor. Current limitations include the relatively small number of experimentally validated predicted neoepitopes (ensuring that predicted neoepitopes elicit a robust T cell response) and the translation of findings from in vitro and in vivo models to human patients. To address these challenges, future efforts should concentrate on developing high-throughput assays, such as peptide–MHC binding assays and T cell activation assays, to efficiently validate large numbers of predicted neoepitopes. Using humanized mouse models, organoid systems, and organ-on-chip systems can help assess the immunogenicity and therapeutic potential of predicted neoepitopes in a more clinically relevant context. Finally, applying iterative cycles of prediction and validation, where computational predictions are continually refined based on experimental results, will enhance the accuracy and reliability of neoepitope prediction.

Translational approaches and cross-disciplinary collaborations among bioinformaticians, immunologists, and clinicians should also be pursued to develop prediction models that are more clinically relevant. Such collaborations can facilitate the creation of prediction models that are both accurate and comprehensive. Another crucial aspect, of continued relevance, is standardization and benchmarking. Establishing standardized benchmarks and datasets for assessing the performance of neoepitope prediction tools should be addressed through the establishment of open access repositories, enabling researchers to share validated neoepitopes and associated data.

In this context, in obtaining and sequencing tumor data, an important challenge faced in the development of therapeutic vaccines is tumor heterogeneity. Tumor heterogeneity refers to the existence of different subclones within a single tumor, which are separated not only spatially but also by distinct mutational patterns. This intratumoral heterogeneity can affect the expression of neoantigens and their immunogenicity. In other words, the type of tumor and the stage of the disease may present tumors with high heterogeneity, diverse mutation profiles, and the expression of neoepitopes, leading to greater complexity in targeting therapies, since the predicted neoepitopes may only be effective against one subset of tumor cells.

Currently, sequencing and bioinformatics tools, such as single-cell RNA sequencing (ScRNA-seq), can be used to acquire high-resolution genomic sequence information, allowing for the provision of the clonal architecture and mutational ancestry of subclones, helping to map the ideal neoantigens to combat the tumor. However, despite being promising, this technique still faces some problems of its own, such as the difficulty of isolating cells and maintaining their integrity and viability for future analyses, the high cost, and challenges in data integration.

In summary, overcoming the limitations in the critical steps of variant calling, neoepitope prediction and prioritization, and validation requires a multifaceted approach. Future studies should leverage advances in sequencing technologies, machine learning, high-throughput screening, and collaborative frameworks to enhance the accuracy, efficiency, and clinical relevance of neoepitope prediction for therapeutic vaccine development. By integrating these approaches, the field can move closer to developing effective personalized cancer vaccines.

## Figures and Tables

**Table 1:** Synthesis matrix of neoepitope identification studies in NGS data.

Ref.	NGS Dataset Used	Type of Tumor	Variant Calling Program/DATABASE	Neoepitope Prediction Programs	MHC Class Used for Predictions	Prediction Selection Scores	Immunological Characteristics Evaluated	In Vivo Validation	In Vitro Validation	In Silico Validation	Clinical Trial	Positive Aspects	Limitations
[19]	NGS RNA-seq of 177 lineages	Melanoma, squamous cell carcinoma, lung carcinoma, gastric adenocarcinoma, Burkitt’s lymphoma, breast carcinoma, colorectal adenocarcinoma, ovarian carcinoma, B-cell lymphocytic leukemia, colorectal carcinoma, cervical adenocarcinoma, liver carcinoma, chronic myeloid leukemia, gastric adenocarcinoma, prostate carcinoma, neuroblastoma, glioblastoma, osteosarcoma	CCLE e COSMIC	NetMHCpan	MHC-I	ic50 ≤ 500 nM	Affinity to MHC-I expression	-	-	HLA confirmation compared to other methods	-	Allows for the selection of cell lines based on HLA expression	The literature: limited immunological characterization
[20]	NGS (WES)	Colorectal carcinoma	Mutect2: snp	NetMHCpan pNovo3	MHC-I	ic50 ≤ 500 nM	MHC-I affinity cleavage processing	-	-	-	-	It is also possible to identify non-linear epitopes and those arising from changes in other stages of antigen processing	The new peptides have not yet been validated—other mechanisms of peptide alteration are not well understood
[21]	NGS (WGS RNA-seq)	Melanoma	myNEO’s (snp and indel) Immuno Engine	MHCflurry neoIM (T cell activation)	MHC-I	ic50 ≤ 500 nM	MHC-I binding expression T cell activation	-	IFN-γ detection	-	-	Presented a cheaper and faster method of constructing mRNA	T cell prediction and activation steps are not described
[22]	NGS (WES)	Breast carcinoma	SAMtools Somatic Sniper VarScan Somatic Strelka (snp)	NetMHC pVACseq	MHC-I	ic50 ≤ 500 nM	MHC-I binding variant frequency expression	Measurement of tumor size and detection of interferon-gamma and TNF-alpha	Activation of CD8+ T cells (detection of interferon-gamma and TNF-alpha)	-	-	It was possible to identify some antigenic neoepitopes for each patient	Does not evaluate other biological characteristics for prediction resulting in a high false negative rate
[23]	NGS (WES e RNA-seq)	Breast carcinoma	Mutect Varscan Somatic Sniper Strelka	NetMHCpan	MHC-I	ic50 ≤ 500 nM	Binding to MHC-I expression	Activation of CD8+ T cells (IFN-γ detection)	-	-	-	These patients harbor a sufficient number of immunogenic neoAgs suitable for vaccine development setting the basis for future combinatorial therapies containing vaccines	Only one allele and mutation type were considered
[24]	NGS (WES e RNA-seq)	Gastric adenocarcinoma	Strelka Somatic Sniper Varscan2 Mutect (snp and indel)	NetMHCpan, NetMHCpan II	MHC-I MHC-II	ic50 ≤ 500 nM	Binding to MHC-I expression	Activation of CD8+ T cells (IFN-γ detection	Activation of CD8+ T cells (IFN-γ detection	-	mRNA vaccine encoding defined neoantigen mutations in driver genes and HLA-I–predicted epitopes in patients with metastatic gastrointestinal cancer	mRNA neoantigen vaccine could also possibly be used to improve adoptive T cell therapy with neoantigen-specific cells by restimulating T cells in vivo	Lack of tumor shrinkage in the present pilot trial
[25]	NGS	Lung carcinoma, breast carcinoma, colorectal carcinoma, glioma, ovarian carcinoma, pancreatic carcinoma, gastric adenocarcinoma, melanoma, liver carcinoma	Filtered germlines from dbSNP; COSMIC	NetMHCpan	MHC-I	ic50 ≤ 500 nM	Proteasome TAP binding to MHC-I and II	-	-	-	-	It is possible to find neoepitopes in common between specific groups of patients	- Some regions of the exome were left out of the analyses; - Only a small portion of the population can benefit from the technique when searching for universal neoantigens
[26]	NGS (WES RNA-seq)	B-cell lymphocytic leukemia	Strelka 2 (snp and indel)	NetMHCpan NetChop	MHC-I	ic50 ≤ 500 nM	MHC-I binding expression transport	-	IFN-γ detection	-	-	Allowed for the identification of disease-specific neoepitopes	Exclusion of many variables associated with the immune response
[27]	NGS (WGS e RNA-seq)	Lung carcinoma	TCGA (snp and indel)	NetMHC	MHC-I	ic50 ≤ 500 nM	Binding to MHC-I expression	-	Peptide expression; confirmation of T cell reactivity	-	-	A new algorithm that evaluates the possible factors that characterize the immunogenicity of a neoepitope	Does not perform a specific variant calling for the samples used
[28]	NGS (WES)	Melanoma	Germline filtered with dbSNP; (snp)	NetMHC	MHC-I	ic50 ≤ 500 nM	Binding to MHC-I expression	-	Activation of CD8+ T cells (interferon- gamma detection)	Docking	-	A single-cell RNA sequencing platform combined with microfluidics and NGS technology will allow every researcher to analyze biomarkers in terms of their genetic phenotypic and even functional properties	Validation for just one HLA allele
[29]	NGS	Melanoma, prostate carcinoma, pancreatic carcinoma, lung carcinoma, colorectal carcinoma, neuroblastoma, glioblastoma, renal carcinoma, myeloblastoma, mesothelioma	Mutect2: snp	pVACtools	MHC-I	ic50 ≤ 500 nM	Binding to MHC-I	-	-	-	-	Confirmation that there is great difficulty in constructing allogeneic vaccines for cancer	- Analysis is limited to a few epitope sizes; - There is no analysis of other biological factors for prioritizing neoepitopes
[30]	NGS (WGS e RNA-seq)	Ovarian carcinoma, lung carcinoma	Samtools (snp and indel)	NetMHCpan	MHC-I	ic50 ≤ 500 nM	Binding to MHC-I	There was activation of T CD8+ and T CD4+, but there was no tumor reduction	Activation of CD8+ T cells (detection of interferon-gamma and TNF-alpha)	-	-	Highlights of the limitation involved in the formulation of neoepitope vaccines in tumors with low mutational load	- Analysis is limited to a few epitope sizes; - There is no analysis of other biological factors for prioritizing neoepitopes
[31]	NGS	Ovarian carcinoma	GATK Haplotype Caller; MuTect; VarScan (snp and indel)	MixMHCpred MixMHC2pred	MHC-I MHC-II	ic50 ≤ 500 nM	Binding to MHC-I and II expression	-	IFN-γ detection	-	PEP-DC	NeoAgs are more likely to elicit strong T cell responses because T cell tolerance does not hamper their immunogenicity and consequently epitope spreading and a broad anti-tumor immune response	The process of sequencing immunopeptidomics analysis peptide manufacturing and good manufacturing practices for the manufacturing of NeoAg vaccines is long and expensive although costs may decrease as a result of technological improvements
[32]	NGS (WES e RNA-seq)	Liver carcinoma	Strelka 2 (snps and indels)	SYFPEITHI netMHC netMHCpan	MHC-I	ic50 ≤ 500 nM	Binding to MHC-I expression	-	Activation of CD8+ T cells (IFN-γ detection)	Identification of neoepitopes identified in TCGA	-	Evidence of results obtained depending on the type of tumor and mutational load	Validation is limited to what exists in the literature, hepatocellular carcinoma genomic data are limited
[33]	NGS (WGS)	Atypical teratoid rhabdoid tumor	Samtools Platypus	VaxiJen NetMHC	MHC-I	ic50 ≤ 500 nM	Binding to MHC-I, 3 physicochemical characteristics	-	Activation of CD8+ T cells (detection of IFN-γ and TNF-alpha)	-	-	It is possible to find immunogenic neoepitopes even in tumors with low mutational load	- Analyses carried out on low-quality samples - Lack of data on the type of tumor studied
[34]	NGS	Embryonal, liver carcinoma, colorectal carcinoma, gastric adenocarcinoma, endometrial, pancreatic carcinoma, ovarian carcinoma, glioma	GATK VarScan2 (snp and indel) FACTERA (fusion)	NetMHC NetMHCpan IEDB e NetMHCII	MHC-I MHC-II	ic50 ≤ 500 nM	Binding to MHC-I and MHC-II	-	Activation of CD8+ T cells (IFN-γ detection)	-	T cells activated for the A*11:01 allele	The neoepitopes selected for immunotherapy generated tumor regression	Difficulty controlling heterogeneous tumor
[35]	NGS (WES RNA-seq)	Breast carcinoma	Mutect 2 (WGS—WES) VarScan2 (WES—RNA-seq)	pVACtools	MHC-I	ic50 ≤ 500 nM	Binding to MHC-I expression	-	-	Intersection of 3 variant calling methods	-	The combination method has potential clinical applications, including vaccine target detection and prediction of therapeutic effects of immune checkpoint inhibitors	No experimental validation
[36]	NGS (WES RNA-seq)	Glioblastoma	Mutect 2 (snp) Indelocator e Strelka (indel)	NetMHCpan	MHC-I	ic50 ≤ 500 nM	Binding to MHC-I expression physicochemical characteristics	-	Activation of CD8+ T cells (detection of IFN-γ and TNF-alpha)	-	Testing for MHC-I allele epitopes included in MHC-II peptides	It was possible to identify epitopes and immune response even though it was a tumor with a low mutational load	- Clinical tests with patients receiving dexamethasone may have affected the T cell response against neoepitope - Few immunological characteristics evaluated
[37]	NGS (WES RNA-seq)	Liver carcinoma	Mutect2 (snp)	NetMHCpan NetMHCpan II	MHC-I MHC-II	ic50 ≤ 500 nM	Binding to MHC-I and II cleavage expression	Activation of CD8+ T cells (detection of interferon-gamma and TNF-alpha)	-	Comparison of mutated and wild-type neoepitopes	-	Generated responses in MHC-I and MHC-II	Test performed on only 2 alleles
[38]	NGS (WES RNA-seq)	Cholangiocarcinoma	Strelka (snp)	SYFPEITHI NetMHC	MHC-I	ic50 ≤ 500 nM	Binding to MHC-I expression	-	Activation of CD8+ T and CD4+ T cells (detection of IFN-γ and TNF-alpha)	-	7 peptides tested 3 generated an immune response and showed no evidence of tumor recurrence	The immune response was efficient and prevented the emergence of new tumors during the period	None of the epitopes were validated by mass spectrometry
[39]	NGS (WES RNA-seq)	Lung carcinoma	GATK	NetMHC	MHC-I	ic50 ≤ 500 nM	Binding to MHC-I expression	-	IFN-γ detection high-throughput imaging	-	-	High-throughput imaging allows for the detection of expressed neoepitopes even at low concentrations; the combined method is highly selective	Few variables were explored
[40]	NGS (WES)	Chronic lymphocytic leukemia	Mutect	NetMHCpan	MHC-I	ic50 ≤ 500 nM	Binding to MHC-I expression	-	MHC binding IFN-γ detection	-	Combined treatment with transplant for 2 patients and vaccine led to tumor remission	Neoepitopes generated response and tumor remission	The number of identified neoepitopes is underestimated due to the cell therapy applied and the lack of sensitivity of the clonal expansion method
[41]	NGS (WES RNA-seq)	Lung carcinoma	TCGA (snp and indel)	NetMHCpan NetMHCIIpan	MHC-I MHC-II	ic50 ≤ 500 nM	Binding to MHC-I expression	-	IFN-γ detection	-	MyVac vaccine in preclinical testing against 18 mutated genes generated a response detected by IFN-γ ELISpot	The in silico approach allowed for the formulation of a vaccine even in a type of tumor with low mutational load	A relatively simple approach may be difficult in rarer alleles

**Table 2:** Synthesis matrix of studies addressing development of new programs for neoepitope prediction.

Ref.	Program Training	Variant Calling Data	MHC-I Neoepitope Size/Alleles	MHC-II Neoepitope Size/Allele	Algorithm/Matrix for Neoepitope Prediction and Prioritization	Training Dataset for Neoepitope Prediction	Neoepitope Prioritization Criteria	Presented Performance
[42]	**3pHLA**	None	9–10 AA, 28 alleles	None	CART algorithm, random forest	Tumor microbiome	Structural analysis	Increase from 0.82 to 0.99
[43]	**FIONA**	None	None	9–25 AA	Convolutional neural networks	Tumor microbiome	None	AUC: 0.94
[44]	**ACME**	None	9–11 AA	None	Neural networks, BLOSUM50	Tumor microbiome	Transport/cleavage; T cell activation	AUC: 0.9 (HLA-A), 0.88 (HLA-B)
[45]	(Unnamed)	None	None	15–20 AA	NetMHCII, z-score (stability)	Tumor microbiome	None	AUC: 0.76
[46]	**DeepImmuno**	None	9–10 AA	None	Convolutional neural networks	Tumor microbiome	None	AUC: 0.85
[47]	**DeepNetBim**	None	9 AA, 104 alleles	None	Convolutional neural networks	Tumor microbiome	None	AUC: 0.93
[48]	**NUCC**	None	8–11 AA	None	Generalized linear model (GLM), random forest (RF), extreme gradient Boosting (Xgboost), gradient learning machine (GBM), fully connected neural network (FCNN)	Tumor microbiome	Binding affinity score, binding stability, and probability of presentation	AUC: 0.85 with XGboot and GMB
[49]	**APPM**	None	8–11 AA	None	Convolutional neural networks	Tumor microbiome (larger proportion of mass spectrometry data)	None	PPV: 0.4
[50]	**INeo-Epp**	None	8–11 AA (does not predict neoepitopes)	None	Random forest, Boruta	Tumor microbiome	Physicochemical features	AUC: 0.78
[51]	**Ivax**	None	9 AA/6 alleles	8 alleles/15–30 AA	Epitope (EpiMatrix, ClustiMer), homology (Conservatrix, JanusMatrix), HLA (EpiAssembler), epitope concatenation (VaccineCAD)	Microbiome	Transport/cleavage; T cell activation; homology	None
[52]	**MHCflurry**	None	8–15 AA	None	neural networks	Tumor microbiome/mass spectrometry	None	AUC: 0.8
[53]	**MHCSeqNet**	None	9–11 AA	None	neural networks	Tumor microbiome	Transport/cleavage	AUC: 0.99 (common alleles), AUC: 0.79 (indistinct alleles)
[54]	**MS2Rescore**	None	None	None	XGBoost	Tumor microbiome	None	PCC: 0.94
[55]	**Ancer**	None	9–10 AA	15–30 AA	EpiMatrix (neoepitope prediction), JanusMatrix (homology)	Tumor microbiome	Homology	AUC: 0.65
[56]	**Neopepsee**	Snp/indel	None	None	NetCTL (nnalign), Gaussian naive Bayes (GNB), locally weighted naive Bayes (LNB), random forest (RF), and support vector machine (SVM)	Tumor microbiome	Transport/cleavage; hydrophobicity, polarity, and charge at positions 2, 3, 5, and 6; molecular size, entropy, differential antigenic index (DAI), and amino acid pairwise contact potentials (AAPPs); gene expression; homology	AUC: >0.9
[57]	**NetMHCpan**	None	8–11 AA	None	Neural networks (nnalign)	Tumor microbiome/mass spectrometry	None	AUC: 0.83—0.97
[58]	**OnionMHC**	None	9 AA/1 alleles	None	Convolutional neural networks, BLOSUM62	Tumor microbiome	Modeling	AUC: 0.83
[59]	**SHERPA**	None	8–11 AA, 167 alleles	None	BLOSUM62	Tumor	T cell activation; gene expression	1.44-fold more accurate
[60]	**ITCell**	None	None	None	Neural networks	Microbiome	Transport/cleavage; modeling	None
[61]	**MARIA**	None	None	8–26 AA	Neural networks	Tumor microbiome	Gene expression	AUC: 0.87
[62]	**ProTECT**	Snp/indel/fusion	9–10 AA	15 AA	NetMHC *	Tumor microbiome	RankBoost; homology	None
[63]	**PTuneos**	Snp/indel	9–11 AA	None	NetMHCpan (neural networks), random forest	Microbiome	T cell activation; gene expression; homology	AUC: 0.65, AUC: 0.83
[64]	**PVACtools**	Snp/indel/fusion	None	None	NetMHCpan, NetMHC, NetMHCcons, PickPocket, SMM, SMMPMBEC, MHCflurry, MHCnuggets, NetMHCIIpan, SMMalign, NNalign, MHCnuggets	Tumor microbiome	Transport/cleavage; gene expression; homology	None
[65]	**PLATO**	Snp/indel	None	None	Bootstrap, random forest	Tumor microbiome/mass spectrometry	Transport/cleavage; F1 score; gene expression	AUC: 0.71–0.86
[66]	**Seq2Neo**	Snp/indel/fusion	8–11 AA	None	Convolutional neural networks	Tumor microbiome	Transport/cleavage; T cell activation; gene expression	AUC: 0.8
[67]	**SIGANEO**	None	8–11 AA	None	NetMHCpan, NetMHCstabpan, generative adversarial network	Tumor	Gene expression; stability; homology	AUROC: 0.94; AUPRC: 0.338

*: all variations of NetMHC, NetMHCII programs provided by IEDB tools.

**Table 3:** Neoepitope predictors, advantages, and disadvantages.

Program	Advantages	Disadvantages
3pHLA (https://github.com/KavrakiLab/3pHLA-score (accessed on 10 July 2024))	- Predicts peptide–HLA binding affinity with focus on personalized cancer vaccines - Considers peptide processing and presentation pathways	- Limited to specific HLA alleles and peptide lengths
ACME (https://github.com/HYsxe/ACME (accessed on 10 July 2024))	- Predicts immunogenicity of neoantigens based on peptide–MHC binding and T cell receptor recognition	- Limited validation in real-world data
Ancer (version not provided)	- Predicts neoantigens from RNA-seq and mass spectrometry data	- Limited to specific types of cancer and data availability
APPM (https://github.com/haoqing12/APPM.git (accessed on 10 July 2024))	- Predicts MHC-binding peptides and their immunogenicity	- Requires specialized knowledge in immunoinformatics
DeepImmuno (Version 1.0)	- Uses deep learning for predicting neoantigens from tumor sequencing data	- Requires large datasets for training
DeepNetBim (https://github.com/Li-Lab-Proteomics/DeepNetBim (accessed on 10 July 2024))	- Deep learning-based tool for neoantigen prediction	- Performance may vary based on dataset quality and size
FIONA (http://therarna.cn/fiona.html (accessed on 10 July 2024))	- Integrates tumor mutations with RNA expression data for neoantigen prediction	- Requires high-quality RNA-seq data - Computationally heavy
INeo-Epp (http://www.biostatistics.online/ineo-epp/neoantigen.php (accessed on 10 July 2024))	- Predicts neoepitopes based on sequence and structural properties of peptides	- Limited to specific HLA alleles and peptide lengths
ITCell (http://salilab.org/itcell (accessed on 10 July 2024))	- Predicts T cell epitopes from tumor mutations and HLA types	- Limited to T cell epitope prediction
Ivax (version not provided)	- Integrates tumor mutations and HLA typing for neoantigen prediction	- Requires comprehensive tumor and HLA data
MARIA (https://maria.stanford.edu/ (accessed on 10 July 2024))	- Predicts neoantigens and their immunogenic potential	- Requires large datasets for training - Computationally heavy
MHCflurry (version 1.2.0)	- Uses deep learning for accurate prediction of peptide–MHC binding affinities	- Requires training for specific MHC alleles - Computationally heavy
MHCSeqNet https://github.com/cmbcu/MHCSeqNet (accessed on 10 July 2024))	- Predicts binding affinity for MHC class I and II alleles using deep learning	- Limited to specific MHC alleles and peptide lengths
MixMHCpred (version 2.0.2)	- Combines predictions from multiple algorithms to improve accuracy	- Results interpretation may be complex - Limited to specific alleles
MixMHC2pred (version 1.1)	- Enhanced version of MixMHCpred with improved prediction accuracy	- Similar challenges as MixMHCpred - Requires validation in specific contexts
MS2Rescore (version 2.1.2)	- Re-evaluates neoantigen predictions based on mass spectrometry data	- Requires experimental validation for accuracy
Neopepsee (http://sourceforge.net/projects/neopepsee/ (accessed on 10 July 2024))	- Predicts neoantigens using machine learning models	- Performance may vary based on dataset quality and size
NetMHC (version 3.0 or higher)	- Predicts peptide–MHC binding affinity for MHC class I alleles	- Limited to most common alleles - Does not consider antigen processing and presentation processes
NetMHCII (version 2.3)	- Predicts binding affinity for MHC class II alleles	- Limited to most common alleles - Sensitivity and specificity can vary
NetMHCIIpan (version 4.3)	- Predicts peptide–MHC binding affinity for MHC class II rare alleles	- Requires large datasets for training
NetMHCpan (version 4.1)	- Predicts binding affinity for rare alleles	- Requires significant computational resources
NUCC (https://github.com/roubaokai/NUCC.git (accessed on 10 July 2024))	- Predicts neoantigens based on somatic mutations and HLA alleles	- Limited documentation and community support
OnionMHC (https://github.com/shikhar249/OnionMHC (accessed on 10 July 2024))	- Integrates multi-omics data for neoantigen prediction	- Complex integration of data sources
PLATO (version not provided)	- Predicts neoantigens from RNA-seq and proteomics data	- Requires high-quality data for accurate predictions
ProTECT (version 2.5.0)	- Predicts neoantigens using ensemble machine learning models	- Requires comprehensive validation in real-world datasets
PTuneos (version 1.0.2)	- Predicts neoantigens from tumor sequencing data using machine learning	- Limited to specific types of cancer and data availability
PVACtools (http://pvactools.org/ (accessed on 10 July 2024))	- Integrates epitope prediction, variant calling, and HLA typing for personalized vaccine design	- Requires proficiency in command-line usage and bioinformatics pipelines
Seq2Neo (prior to 2.1)	- Predicts neoantigens using sequence-based features	- Performance may vary based on dataset quality and size
SHERPA (version not provided)	- Predicts neoantigens and their potential immunogenicity	- Performance may vary based on dataset quality and size
SIGANEO (https://github.com/NCTool/SIGANEO-GAN (accessed on 10 July 2024))	- Uses deep learning for neoantigen prediction	- Requires extensive computational resources - Limited to specific datasets and applications
SYFPEITHI (version 1.0)	- Provides experimental binding data and prediction of MHC ligands	- Limited to experimental data for prediction - Coverage may be limited for some alleles

**Table 4:** Prediction and prioritization algorithms, advantages, and disadvantages.

Algorithm	Advantages	Disadvantages
Artificial Neural Network	- Powerful for complex pattern recognition tasks - Can learn non-linear relationships	- Requires large amounts of data for training - Prone to overfitting
Convolutional Neural Network	- Automatically learns hierarchical features	- Computationally heavy - Requires large datasets for training
Random Forest	- Handles high-dimensional data well	- Can be slow for real-time prediction - Lack of transparency in complex ensembles
EpiMatrix	- Designed specifically for epitope prediction tasks - Can identify less frequent epitopes	- Computationally heavy
JanusMatrix	- Specialized for protein interaction prediction	- Require adequation for different datasets
XGBoost	- High predictive power - Handles missing data well	- Sensitive to overfitting
CART Algorithm	- Easy and intuitive to interpret	- Sensitive to overfitting - Instability with small variations in data
BLOSUM	- Effective for sequence alignment in bioinformatics	- Limited to specific sequence alignment tasks
Boruta	- Can learn non-linear relationships - Easy to use	- Computationally heavy - Can be slow with large datasets
ClustiMer	- Effective in identifying motifs in biological sequences	- Limited to biological sequence analysis - Requires specific input formats
Conservatrix	- Designed for conservation analysis in bioinformatics	- Limited applicability outside of biological conservation studies
EpiAssembler	- Assembly of epitope sequences	- May require extensive pre-processing of data
VaccineCAD	- Supports vaccine design and evaluation	- Interpretation of results requires expertise in bioinformatics and immunology
Gaussian Naive Bayes	- Performs well with small datasets	- Not suitable for complex data
Locally Weighted Naive Bayes	- Handles non-linear relationships	- Computationally heavy
Support Vector Machine	- Effective in high-dimensional spaces	- Requires careful selection of kernel functions - High memory consumption for large datasets
Bootstrap	- Robust estimation of statistical measures	- Can be computationally heavy - Requires multiple iterations for reliable results
Generalized Linear Model	- Flexible in modeling various types of data	- Assumes linear relationship between predictors and response
Gradient Learning Machine	- Efficient learning with large datasets	- Requires tuning for optimal performance
Fully Connected Neural Network	- Suitable for a wide range of applications	- Prone to overfitting - Requires large amounts of data for training
Generative Adversarial Network	- Generates synthetic data - Useful for unsupervised learning	- Can be unstable when training

## Supplementary Materials

The following supporting information can be downloaded at: https://www.mdpi.com/article/10.3390/vaccines12080836/s1, Table S1: Reviewed articles excluded due to the adopted criteria—exclusion.pdf; Table S2: Types of tumor by articles; Table S3: HLA-I allele groups; Table S4: HLA-II allele groups, Table S5: Algorithms in review; PRISMA-ScR-Fillable-Checklist [18].

## References

[1] Schmidt J., Smith A.R., Magnin M., Racle J., Devlin J.R., Bobisse S., Cesbron J., Bonnet V., Carmona S.J., Huber F. (2021). Prediction of Neo-Epitope Immunogenicity Reveals TCR Recognition Determinants and Provides Insight into Immunoediting. Cell Rep. Med..

[2] Lundegaard C., Lamberth K., Harndahl M., Buus S., Lund O., Nielsen M. (2018). NetMHC-3.0: Accurate web accessible predictions of human, mouse and monkey MHC class I affinities for peptides of length 8–11. Nucleic Acids Res..

[3] Brennick C.A., George M.M., Corwin W.L., Srivastava P.K., Ebrahimi-Nik H. (2017). Neoepitopes as cancer immunotherapy targets: Key challenges and opportunities. Immunotherapy.

[4] Nagpal G., Chaudhary K., Agrawal P., Raghava G.P.S. (2018). Computer-aided prediction of antigen presenting cell modulators for designing peptide-based vaccine adjuvants. J. Transl. Med..

[5] Tomar N., De R.K. (2010). Immunoinformatics: An Integrated Scenario. Immunology.

[6] Reardon B., Koşaloğlu-Yalçın Z., Paul S., Peters B., Sette A. (2021). Allele-specific thresholds of eluted ligands for T-cell epitope prediction. Mol.Cell. Proteom..

[7] Ghosh M., Di Marco M., Stevanović S. (2019). Identification of MHC Ligands and Establishing MHC Class I Peptide Motifs. Met. Mol. Biol..

[8] Wang P., Sidney J., Kim Y., Sette A., Lund O., Nielsen M., Peters B. (2010). Peptide Binding Predictions for HLA DR, DP and DQ Molecules. BMC Bioinform..

[9] Lv J., Zhu Y., Ji A., Zhang Q., Liao G. (2020). Mining TCGA Database for Tumor Mutation Burden and Their Clinical Significance in Bladder Cancer. Biosci. Rep..

[10] Suda K., Tomizawa K., Mitsudomi T. (2010). Biological and Clinical Significance of KRAS Mutations in Lung Cancer: An Oncogenic Driver That Contrasts with EGFR Mutation. Cancer Metastasis Rev..

[11] Tomczak K., Czerwińska P., Wiznerowicz M. (2015). Review The Cancer Genome Atlas (TCGA): An Immeasurable Source of Knowledge. Contemp. Oncol..

[12] Walter K., Min J.L., Huang J., Crooks L., Memari Y., McCarthy S., Perry J.R.B., Xu C., The UK10K Consortium, Writing Group (2015). The UK10K Project Identifies Rare Variants in Health and Disease. Nature.

[13] Butterfield L.H. (2015). Cancer Vaccines. BMJ.

[14] Sanchez-Trincado J.L., Gomez-Perosanz M., Reche P.A. (2017). Fundamentals and methods for T- and B-cell epitope prediction. J. Immunol. Res..

[15] Moreira A.L., Eng J. (2014). Personalized Therapy for Lung Cancer. Chest.

[16] Behjati S., Tarpey P.S. (2013). What Is next Generation Sequencing?. Arch. Dis. Child. Educ. Pract. Ed..

[17] Blanc E., Holtgrewe M., Dhamodaran A., Messerschmidt C., Willimsky G., Blankenstein T., Beule D. (2019). Identification and Ranking of Recurrent Neo-Epitopes in Cancer. BMC Med. Genom..

[18] Tricco A.C., Lillie E., Zarin W., O’Brien K.K., Colquhoun H., Levac D., Moher D., Peters M.D., Horsley T., Weeks L. (2018). PRISMA Extension for Scoping Reviews (PRISMAScR): Checklist and Explanation. Ann. Intern. Med..

[19] Boegel S., Löwer M., Bukur T., Sahin U., Castle J.C. (2014). A Catalog of HLA Type, HLA Expression, and Neo-Epitope Candidates in Human Cancer Cell Lines. Oncoimmunology.

[20] Xiang H., Zhang L., Bu F., Guan X., Chen L., Zhang H., Zhao Y., Chen H., Zhang W., Li Y. (2022). A Novel Proteogenomic Integration Strategy Expands the Breadth of Neo-Epitope Sources. Cancers.

[21] de Mey W., De Schrijver P., Autaers D., Pfitzer L., Fant B., Locy H., Esprit A., Lybaert L., Bogaert C., Verdonck M. (2022). A Synthetic DNA Template for Fast Manufacturing of Versatile Single Epitope MRNA. Mol. Ther. Nucleic Acids.

[22] Zhang X., Kim S., Hundal J., Herndon J., Li S., Petti A., Soysal S., Li L., McLellan M., Hoog J. (2017). Breast Cancer Neoantigens Can Induce CD8^+^ T-Cell Responses and Antitumor Immunity. Cancer Immunol. Res..

[23] Aparicio B., Repáraz D., Ruiz M., Llopiz D., Silva L., Vercher E., Theunissen P., Tamayo I., Smerdou C., Igea A. (2022). Identification of HLA Class I-Restricted Immunogenic Neoantigens in Triple Negative Breast Cancer. Front. Immunol..

[24] Cafri G., Gartner J.J., Zaks T., Hopson K., Levin N., Paria B.C., Parkhurst M.R., Yossef R., Lowery F.J., Jafferji M.S. (2020). MRNA Vaccine-Induced Neoantigen-Specific T Cell Immunity in Patients with Gastrointestinal Cancer. J. Clin. Investig..

[25] Hartmaier R.J., Charo J., Fabrizio D., Goldberg M.E., Albacker L.A., Pao W., Chmielecki J. (2017). Genomic Analysis of 63,220 Tumors Reveals Insights into Tumor Uniqueness and Targeted Cancer Immunotherapy Strategies. Genome Med..

[26] Thakur S., Jain M., Zhang C., Major C., Bielamowicz K.J., Lacayo N.J., Vaske O., Lewis V., Murguia-Favela L., Narendran A. (2021). Identification and in Vitro Validation of Neoantigens for Immune Activation against High-Risk Pediatric Leukemia Cells. Hum. Vaccin. Immunother..

[27] Reimann H., Nguyen A., Sanborn J.Z., Vaske C.J., Benz S.C., Niazi K., Rabizadeh S., Spilman P., Mackensen A., Ruebner M. (2021). Identification and Validation of Expressed HLA-Binding Breast Cancer Neoepitopes for Potential Use in Individualized Cancer Therapy. J. Immunother. Cancer.

[28] Nonomura C., Otsuka M., Kondou R., Iizuka A., Miyata H., Ashizawa T., Sakura N., Yoshikawa S., Kiyohara Y., Ohshima K. (2019). Identification of a Neoantigen Epitope in a Melanoma Patient with Good Response to Anti-PD-1 Antibody Therapy. Immunol. Lett..

[29] James C.A., Ronning P., Cullinan D., Cotto K.C., Barnell E.K., Campbell K.M., Skidmore Z.L., Sanford D.E., Goedegebuure S.P., Gillanders W.E. (2021). In Silico Epitope Prediction Analyses Highlight the Potential for Distracting Antigen Immunodominance with Allogeneic Cancer Vaccines. Cancer Res. Commun..

[30] Martin S.D., Brown S.D., Wick D.A., Nielsen J.S., Kroeger D.R., Twumasi-Boateng K., Holt R.A., Nelson B.H. (2016). Low Mutation Burden in Ovarian Cancer May Limit the Utility of Neoantigen-Targeted Vaccines. PLoS ONE.

[31] Sarivalasis A., Boudousquié C., Balint K., Stevenson B.J., Gannon P.O., Iancu E.M., Rossier L., Martin Lluesma S., Mathevet P., Sempoux C. (2019). A Phase I/II Trial Comparing Autologous Dendritic Cell Vaccine Pulsed Either with Personalized Peptides (PEP-DC) or with Tumor Lysate (OC-DC) in Patients with Advanced High-Grade Ovarian Serous Carcinoma. J. Transl. Med..

[32] Löffler M., Mohr C., Bichmann L., Freudenmann L., Walzer M., Schroeder C., Trautwein N., Hilke F., Zinser R., Mühlenbruch L. (2019). Multi-Omics Discovery of Exome-Derived Neoantigens in Hepatocellular Carcinoma. Genome Med..

[33] Marcu A., Schlosser A., Keupp A., Trautwein N., Johann P., Wölfl M., Lager J., Monoranu C.M., Walz J.S., Henkel L.M. (2021). Natural and Cryptic Peptides Dominate the Immunopeptidome of Atypical Teratoid Rhabdoid Tumors. J. ImmunoTherapy Cancer.

[34] Chen F., Zou Z., Du J., Su S., Shao J., Meng F., Yang J., Xu Q., Ding N., Yang Y. (2019). Neoantigen Identification Strategies Enable Personalized Immunotherapy in Refractory Solid Tumors. J. Clin. Investig..

[35] Hashimoto S., Noguchi E., Bando H., Miyadera H., Morii W., Nakamura T., Hara H. (2021). Neoantigen Prediction in Human Breast Cancer Using RNA Sequencing Data. Cancer Sci..

[36] Keskin D., Anandappa A., Sun J., Tirosh I., Mathewson N., Li S., Oliveira G., Giobbie-Hurder A., Felt K., Gjini E. (2019). Neoantigen Vaccine Generates Intratumoral T Cell Responses in Phase Ib Glioblastoma Trial. Nature.

[37] Repáraz D., Ruiz M., Llopiz D., Silva L., Vercher E., Aparicio B., Egea J., Tamayo-Uria I., Hervás-Stubbs S., García-Balduz J. (2022). Neoantigens as Potential Vaccines in Hepatocellular Carcinoma. J. Immunother. Cancer.

[38] Löffler M.W., Chandran P.A., Laske K., Schroeder C., Bonzheim I., Walzer M., Hilke F.J., Trautwein N., Kowalewski D.J., Schuster H. (2016). Personalized Peptide Vaccine-Induced Immune Response Associated with Long-Term Survival of a Metastatic Cholangiocarcinoma Patient. J. Hepatol..

[39] Han K.-C., Park D., Ju S., Lee Y.E., Heo S.-H., Kim Y.-A., Lee J.E., Lee Y., Park K.H., Park S.-H. (2020). Streamlined Selection of Cancer Antigens for Vaccine Development through Integrative Multi-Omics and High-Content Cell Imaging. Sci. Rep..

[40] Rajasagi M., Shukla S.A., Fritsch E.F., Keskin D.B., DeLuca D., Carmona E., Zhang W., Sougnez C., Cibulskis K., Sidney J. (2014). Systematic Identification of Personal Tumor-Specific Neoantigens in Chronic Lymphocytic Leukemia. Blood.

[41] McCann K., von Witzleben A., Thomas J., Wang C., Wood O., Singh D., Boukas K., Bendjama K., Silvestre N., Nielsen F.C. (2022). Targeting the Tumor Mutanome for Personalized Vaccination in a TMB Low Non-Small Cell Lung Cancer. J. Immunother. Cancer.

[42] Conev A., Devaurs D., Rigo M.M., Antunes D.A., Kavraki L.E. (2022). 3pHLA-Score Improves Structure-Based Peptide-HLA Binding Affinity Prediction. Sci. Rep..

[43] Xu S., Wang X., Fei C. (2022). A Highly Effective System for Predicting MHC-II Epitopes with Immunogenicity. Front. Oncol..

[44] Hu Y., Wang Z., Hu H., Wan F., Chen L., Xiong Y., Wang X., Zhao D., Huang W., Zeng J. (2019). ACME: Pan-Specific Peptide-MHC Class i Binding Prediction through Attention-Based Deep Neural Networks. Bioinformatics.

[45] Mettu R., Charles T., Landry S. (2016). CD4^+^T-Cell Epitope Prediction Using Antigen Processing Constraints. J. Immunol. Methods.

[46] Li G., Iyer B., Prasath V.B.S., Ni Y., Salomonis N. (2021). DeepImmuno: Deep Learning-Empowered Prediction and Generation of Immunogenic Peptides for T Cell Immunity. Brief. Bioinform..

[47] Yang X., Zhao L., Wei F., Li J. (2021). DeepNetBim: Deep Learning Model for Predicting HLA-Epitope Interactions Based on Network Analysis by Harnessing Binding and Immunogenicity Information. BMC Bioinform..

[48] Xin K., Wei X., Shao J., Chen F., Liu Q., Liu B. (2024). Establishment of a Novel Tumor Neoantigen Prediction Tool for Personalized Vaccine Design. Hum. Vaccin. Immunother..

[49] Hao Q., Wei P., Shu Y., Zhang Y.-G., Xu H., Zhao J.-N. (2021). Improvement of Neoantigen Identification Through Convolution Neural Network. Front. Immunol..

[50] Wang G., Wan H., Jian X., Li Y., Ouyang J., Tan X., Zhao Y., Lin Y., Xie L. (2020). INeo-Epp: A Novel T-Cell HLA Class-I Immunogenicity or Neoantigenic Epitope Prediction Method Based on Sequence-Related Amino Acid Features. Biomed. Res. Int..

[51] Moise L., Gutierrez A., Kibria F., Martin R., Tassone R., Liu R., Terry F., Martin B., De Groot A.S. (2015). IVAX: An Integrated Toolkit for the Selection and Optimization of Antigens and the Design of Epitope-Driven Vaccines. Hum. Vaccin. Immunother..

[52] O’Donnell T.J., Rubinsteyn A., Bonsack M., Riemer A.B., Laserson U., Hammerbacher J. (2018). MHCflurry: Open-Source Class I MHC Binding Affinity Prediction. Cell Syst..

[53] Phloyphisut P., Pornputtapong N., Sriswasdi S., Chuangsuwanich E. (2019). MHCSeqNet: A Deep Neural Network Model for Universal MHC Binding Prediction. BMC Bioinform..

[54] Declercq A., Bouwmeester R., Hirschler A., Carapito C., Degroeve S., Martens L., Gabriels R. (2022). MS2Rescore: Data-Driven Rescoring Dramatically Boosts Immunopeptide Identification Rates. Mol. Cell. Proteom..

[55] Richard G., De Groot A.S., Steinberg G.D., Garcia T.I., Kacew A., Ardito M., Martin W.D., Berdugo G., Princiotta M.F., Balar A.V. (2021). Multi-Step Screening of Neoantigens’ HLA- and TCR-Interfaces Improves Prediction of Survival. Sci. Rep..

[56] Kim S., Kim H.S., Kim E., Lee M.G., Shin E.-C., Paik S., Kim S. (2018). Neopepsee: Accurate Genome-Level Prediction of Neoantigens by Harnessing Sequence and Amino Acid Immunogenicity Information. Ann. Oncol..

[57] Jurtz V., Paul S., Andreatta M., Marcatili P., Peters B., Nielsen M. (2017). NetMHCpan-4.0: Improved Peptide-MHC Class I Interaction Predictions Integrating Eluted Ligand and Peptide Binding Affinity Data. J. Immunol..

[58] Saxena S., Animesh S., Fullwood M.J., Mu Y. (2020). Onionmhc: A Deep Learning Model for Peptide—Hla-A*02:01 Binding Predictions Using Both Structure and Sequence Feature Sets. J. Micromechanics Mol. Phys..

[59] Pyke R.M., Mellacheruvu D., Dea S., Abbott C.W., Zhang S.V., Phillips N.A., Harris J., Bartha G., Desai S., McClory R. (2021). Precision Neoantigen Discovery Using Large-Scale Immunopeptidomes and Composite Modeling of MHC Peptide Presentation. Mol. Cell Proteom..

[60] Schneidman-Duhovny D., Khuri N., Dong G.Q., Winter M.B., Shifrut E., Friedman N., Craik C.S., Pratt K.P., Paz P., Aswad F. (2018). Predicting CD4^+^ T-Cell Epitopes Based on Antigen Cleavage, MHCII Presentation, and TCR Recognition. PLoS ONE.

[61] Chen B., Khodadoust M.S., Olsson N., Wagar L.E., Fast E., Liu C.L., Muftuoglu Y., Sworder B.J., Diehn M., Levy R. (2019). Predicting HLA Class II Antigen Presentation through Integrated Deep Learning. Nat. Biotechnol..

[62] Rao A.A., Madejska A.A., Pfeil J., Paten B., Salama S.R., Haussler D. (2020). ProTECT—Prediction of T-Cell Epitopes for Cancer Therapy. Front. Immunol..

[63] Zhou C., Wei Z., Zhang Z., Zhang B., Zhu C., Chen K., Chuai G., Qu S., Xie L., Gao Y. (2019). PTuneos: Prioritizing Tumor Neoantigens from next-Generation Sequencing Data. Genome Med..

[64] Hundal J., Kiwala S., McMichael J., Miller C.A., Xia H., Wollam A.T., Liu C.J., Zhao S., Feng Y.-Y., Graubert A.P. (2020). PVACtools: A Computational Toolkit to Identify and Visualize Cancer Neoantigens. Cancer Immunol. Res..

[65] Sherafat E., Force J., Măndoiu I.I. (2020). Semi-Supervised Learning for Somatic Variant Calling and Peptide Identification in Personalized Cancer Immunotherapy. BMC Bioinform..

[66] Diao K., Chen J., Wu T., Wang X., Wang G., Sun X., Zhao X., Wu C., Wang J., Yao H. (2022). Seq2Neo: A Comprehensive Pipeline for Cancer Neoantigen Immunogenicity Prediction. Int. J. Mol. Sci..

[67] Ye Y., Shen Y., Wang J., Li D., Zhu Y., Zhao Z., Pan Y., Wang Y., Liu X., Wan J. (2023). SIGANEO: Similarity Network with GAN Enhancement for Immunogenic Neoepitope Prediction. Comput. Struct. Biotechnol. J..

[68] Alexandrov L.B., Nik-Zainal S., Wedge D.C., Aparicio S.A.J.R., Behjati S., Biankin A.V., Australian Pancreatic Cancer Genome Initiative, ICGC Breast Cancer Consortium, ICGC MMML-Seq Consortium, ICGC PedBrain (2013). Signatures of Mutational Processes in Human Cancer. Nature.

[69] Hilf N., Kuttruff-Coqui S., Frenzel K., Bukur V., Stevanović S., Gouttefangeas C., Platten M., Tabatabai G., Dutoit V., Van Der Burg S.H. (2019). Actively Personalized Vaccination Trial for Newly Diagnosed Glioblastoma. Nature.

[70] Martínez-Pérez E., Molina-Vila M.A., Marino-Buslje C. (2021). Panels and Models for Accurate Prediction of Tumor Mutation Burden in Tumor Samples. NPJ Precis. Oncol..

[71] Blaeschke F., Paul M.C., Schuhmann M.U., Rabsteyn A., Schroeder C., Casadei N., Matthes J., Mohr C., Lotfi R., Wagner B. (2019). Low Mutational Load in Pediatric Medulloblastoma Still Translates into Neoantigens as Targets for Specific T-Cell Immunotherapy. Cytotherapy.

[72] World Health Organization (2023). World Health Statistics 2023: Monitoring Health for the SDGs, Sustainable Development Goals.

[73] Vivekanandhan S., Bahr D., Kothari A., Ashary M.A., Baksh M., Gabriel E. (2023). Immunotherapies in rare cancers. Mol. Cancer.

[74] Koboldt D.C. (2020). Best Practices for Variant Calling in Clinical Sequencing. Genome Med..

[75] do Valle Í.F., Giampieri E., Simonetti G., Padella A., Manfrini M., Ferrari A., Castellani G. (2016). Optimized pipeline of MuTect and GATK tools to improve the detection of somatic single nucleotide polymorphisms in whole-exome sequencing data. BMC Bioinform..

[76] Chen Z., Yuan Y., Chen X., Chen J., Lin S., Li X., Du H. (2020). Systematic Comparison of Somatic Variant Calling Performance among Different Sequencing Depth and Mutation Frequency. Sci. Rep..

[77] Ghandi M., Huang F.W., Jané-Valbuena J., Kryukov G.V., Lo C.C., McDonald E.R., Barretina J., Gelfand E.T., Bielski C.M., Li H. (2019). Next-generation characterization of the Cancer Cell Line Encyclopedia. Nature.

[78] Tate J.G., Bamford S., Jubb H.C., Sondka Z., Beare D.M., Bindal N., Boutselakis H., Cole C.G., Creatore C., Dawson E. (2019). COSMIC: The Catalogue Of Somatic Mutations In Cancer. Nucleic Acids Res..

[79] Sherry S.T., Ward M.H., Kholodov M., Baker J., Phan L., Smigielski E.M., Sirotkin K. (2001). dbSNP: The NCBI database of genetic variation. Nucleic Acids Res..

[80] Broeckx B.J.G., Peelman L., Saunders J.H., Deforce D., Clement L. (2017). Using variant databases for variant prioritization and to detect erroneous genotype-phenotype associations. BMC Bioinform..

[81] Mullaney J.M., Mills R.E., Pittard W.S., Devine S.E. (2010). Small Insertions and Deletions (INDELs) in Human Genomes. Hum. Mol. Genet..

[82] Weber J.L., David D., Heil J., Fan Y., Zhao C., Marth G. (2002). Human diallelic insertion/deletion polymorphisms. Am. J. Hum. Genet..

[83] Cibulskis K., Lawrence M.S., Carter S.L., Sivachenko A., Jaffe D., Sougnez C., Gabriel S., Meyerson M., Lander E.S., Getz G. (2013). Sensitive Detection of Somatic Point Mutations in Impure and Heterogeneous Cancer Samples. Nat. Biotechnol..

[84] Trevarton A.J., Chang J.T., Symmans W.F. (2023). Simple combination of multiple somatic variant callers to increase accuracy. Sci. Rep..

[85] Fennemann F.L., de Vries I.J.M., Figdor C.G., Verdoes M. (2019). Attacking Tumors From All Sides: Personalized Multiplex Vaccines to Tackle Intratumor Heterogeneity. Front. Immunol..

[86] Van Dijk E.L., Jaszczyszyn Y., Naquin D., Thermes C. (2018). The Third Revolution in Sequencing Technology. Trends Genet..

[87] Wang Z., Liu X., Yang B.-Z., Gelernter J. (2013). The Role and Challenges of Exome Sequencing in Studies of Human Diseases. Front. Genet..

[88] Ding S., Chen X., Shen K. (2020). Single-cell RNA sequencing in breast cancer: Understanding tumor heterogeneity and paving roads to individualized therapy. Cancer Commun..

[89] Robinson J., Barker D.J., Georgiou X., Cooper M.A., Flicek P., Marsh S.G.E. (2020). IPD-IMGT/HLA Database. Nucleic Acids Res..

[90] Hurley C.K., Kempenich J., Wadsworth K., Sauter J., Hofmann J.A., Schefzyk D., Schmidt A.H., Galarza P., Cardozo M.B.R., Dudkiewicz M. (2020). Common, Intermediate and Well-documented HLA Alleles in World Populations: CIWD Version 3.0.0. HLA.

[91] Gonzalez-Galarza F.F., McCabe A., Santos E.J.M.D., Jones J., Takeshita L., Ortega-Rivera N.D., Cid-Pavon G.M.D., Ramsbottom K., Ghattaoraya G., Alfirevic A. (2020). Allele Frequency Net Database (AFND) 2020 Update: Gold-Standard Data Classification, Open Access Genotype Data and New Query Tools. Nucleic Acids Res..

[92] Roider J., Meissner T., Kraut F., Vollbrecht T., Stirner R., Bogner J.R., Draenert R. (2014). Comparison of Experimental Fine-mapping to in Silico Prediction Results of HIV-1 Epitopes Reveals Ongoing Need for Mapping Experiments. Immunology.

[93] Momburg F., Roelse J., Hämmerling G.J., Neefjes J.J. (1994). Peptide size selection by the major histocompatibility complex-encoded peptide transporter. J. Exp. Med..

[94] Sahin U., Derhovanessian E., Miller M., Kloke B.-P., Simon P., Löwer M., Bukur V., Tadmor A.D., Luxemburger U., Schrörs B. (2017). Personalized RNA Mutanome Vaccines Mobilize Poly-Specific Therapeutic Immunity against Cancer. Nature.

[95] Falk K., Rötzschke O., Stevanovié S., Jung G., Rammensee H.-G. (1991). Allele-Specific Motifs Revealed by Sequencing of Self-Peptides Eluted from MHC Molecules. Nature.

[96] Lundegaard C., Lund O., Nielsen M. (2011). Prediction of Epitopes Using Neural Network Based Methods. J. Immunol. Methods.

[97] Luo H., Ye H., Ng H.W., Shi L., Tong W., Mendrick D.L., Hong H. (2015). Machine Learning Methods for Predicting HLA-Peptide Binding Activity. Bioinform. Biol. Insights.

[98] Jesdale B.M., Deocampo G., Meisell J., Beall J., Marinello M.J., Chicz R.M., De Groot A.S. (1997). Matrix-based prediction of MHC-binding peptides: The EpiMatrix algorithm, reagent for HIV research. Vaccines.

[99] Chen T., Guestrin C. (2016). XGBoost: A Scalable Tree Boosting System. Proceedings of the 22nd ACM SIGKDD International Conference on Knowledge Discovery and Data Mining (KDD ‘16).

[100] Ghansah B., Wu S., Ghansah N. Rankboost-based result merging. Proceedings of the International Conference on Computer and Information Technology; Ubiquitous Computing and Communications; Dependable, Autonomic and Secure Computing; Pervasive Intelligence and Computing.

[101] Song D., Chen J., Chen G., Li N., Li J., Fan J., Li S.C. (2014). Parameterized BLOSUM matrices for protein alignment. Trans. Comput. Biol. Bioinform..

[102] Rigatti S.J. (2017). Random Forest. J. Insur. Med..

[103] Hesterberg T. (2011). Bootstrap. Wiley Interdiscip. Rev. Comput. Stat..

[104] Freitas P., Silva F., Sousa J.V., Ferreira R.M., Figueiredo C., Pereira T., Oliveira H.P. (2023). Machine learning-based approaches for cancer prediction using microbiome data. Sci. Rep..

[105] Bassani-Sternberg M., Pletscher-Frankild S., Jensen L.J., Mann M. (2015). Mass Spectrometry of Human Leukocyte Antigen Class I Peptidomes Reveals Strong Effects of Protein Abundance and Turnover on Antigen Presentation. Mol. Cell. Proteom..

[106] Blum J.S., Wearsch P.A., Cresswell P. (2013). Pathways of Antigen Processing. Annu. Rev. Immunol..

[107] Alspach E., Lussier D.M., Miceli A.P., Kizhvatov I., DuPage M., Luoma A.M., Meng W., Lichti C.F., Esaulova E., Vomund A.N. (2019). MHC-II Neoantigens Shape Tumour Immunity and Response to Immunotherapy. Nature.

[108] Reynisson B., Alvarez B., Paul S., Peters B., Nielsen M. (2020). NetMHCpan-4.1 and NetMHCIIpan-4.0: Improved Predictions of MHC Antigen Presentation by Concurrent Motif Deconvolution and Integration of MS MHC Eluted Ligand Data. Nucleic Acids Res..

[109] Fritsch E.F., Rajasagi M., Ott P.A., Brusic V., Hacohen N., Wu C.J. (2014). HLA-binding properties of tumor neoepitopes in humans. Cancer Immunol. Res..

[110] Bulik-Sullivan B., Busby J., Palmer C.D., Davis M.J., Murphy T., Clark A., Busby M., Duke F., Yang A., Young L. (2018). Deep learning using tumor HLA peptide mass spectrometry datasets improves neoantigen identification. Nat. Biotechnol..

[111] O’Donnell T.J., Rubinsteyn A., Laserson U. (2020). MHCflurry 2.0: Improved Pan-Allele Prediction of MHC Class I-Presented Peptides by Incorporating Antigen Processing. Cell Syst..

[112] Vitiello A., Zanetti M. (2017). Neoantigen prediction and the need for validation. Nat. Biotechnol..

