# Supplementary material for: Tumor Neoepitope-Based Vaccines: A Scoping Review on Current Predictive Computational Strategies
Source: Vaccines (Basel). 2024 Jul 24;12(8):836. doi: 10.3390/vaccines12080836 (PMC11360805; doi:10.3390/vaccines12080836)
Supplement: Supplementary file 1 [file vaccines-12-00836-s001.zip › Table S2_ Types of tumor by articles.pdf]

| Types of tumor | Reference | Group |
|---|---|---|
| lung carcinoma | 18, 24, 26, 28, 29, 38, 40 | A |
| breast carcinoma | 18, 21, 22, 24, 34 | B |
| colorectal carcinoma | 18, 19, 24, 28, 33 | B |
| liver carcinoma | 18, 24, 31, 33, 36 | B |
| ovarian carcinoma | 18, 24, 29, 30, 33 | B |
| melanoma | 18, 20, 24, 27, 28 | B |
| gastric adenocarcinoma | 18, 23, 24, 33 | C |
| glioblastoma | 18, 28, 35 | D |
| pancreatic carcinoma | 24, 28, 33 | D |
| B-cell lymphocytic leukemia | 18, 25 | E |
| glioma | 24, 33 | E |
| prostate carcinoma | 18, 28 | E |
| neuroblastoma | 18, 28 | E |
| Burkitt's lymphoma | 18 | F |
| cervical adenocarcinoma | 18 | F |
| cholangiocarcinoma | 37 | F |
| chronic lymphocytic leukemia | 39 | F |
| chronic myeloid leukemia | 18 | F |
| colorectal adenocarcinoma | 18 | F |
| embryonal | 33 | F |
| endometrial | 33 | F |
| osteosarcoma | 18 | F |
| renal carcinoma | 28 | F |
| mesothelioma | 28 | F |
| myeloblastoma | 28 | F |
| squamous cell carcinoma | 18 | F |
| teratoid/rhabditoid tumor | 32 | F |
