# Supplementary material for: Tumor Neoepitope-Based Vaccines: A Scoping Review on Current Predictive Computational Strategies
Source: Vaccines (Basel). 2024 Jul 24;12(8):836. doi: 10.3390/vaccines12080836 (PMC11360805; doi:10.3390/vaccines12080836)
Supplement: Supplementary file 1 [file vaccines-12-00836-s001.zip › Table S4 _ HLA-II alleles groups.pdf]

HLA-II allele	Reference	Group
DQB1*03:01, DQB1*06:03, DRB1*12:01, DRB1*13:01	33, 40	A
DPB1*03:01, DPB1*04:01	40	B
DQB1*02:01, DQB1*02:02, DQB1*03:02, DQB1*03:03, DQB1*04:01, DQB1*04:02, DQB1*05:01, DQB1*06:01, DQB1*06:02, DQB1*11:01, DQB1*15:01, DRB1*01:02, DRB1*03:01, DRB1*04:04, DRB1*04:05, DRB1*04:06, DRB1*07:01, DRB1*08:02, DRB1*08:03, DRB1*09:01, DRB1*11:01, DRB1*12:02, DRB1*15:01, DRB1*15:02	33	B
DRB1*01	36	B
