# Supplementary material for: Tumor Neoepitope-Based Vaccines: A Scoping Review on Current Predictive Computational Strategies
Source: Vaccines (Basel). 2024 Jul 24;12(8):836. doi: 10.3390/vaccines12080836 (PMC11360805; doi:10.3390/vaccines12080836)
Supplement: Supplementary file 1 [file vaccines-12-00836-s001.zip › Table S5_ Algorithms in the review.pdf]

Algorithm	Articles
Artificial Neural Network	18, 19, 20, 21, 22, 23, 24, 25, 26, 27, 28, 29, 30, 31, 32, 33, 34, 35, 36, 37, 38, 39, 40, 43, 44, 51, 52, 55, 56, 59, 60, 61, 62, 63, 66
Convolutional Neural Network	42, 45, 46, 57, 65
Random Forest	49, 55, 62, 64
EpiMatrix	50, 54
JanusMatrix	50, 54
XGBoost	47, 53
CART Algorithm	41
BLOSUM50	43
Boruta	49
ClustiMer	50
Conservatrix	50
EpiAssembler	50
VaccineCAD	50
Gaussian Naive Bayes	55
Locally Weighted Naive Bayes	55
Support Vector Machine	55
BLOSUM62	57
Bootstrap	64
Object Pascoal	31
Generalized Linear Model	47
Gradient Learning Machine	47
Fully Connected Neural Network	47
Generative Adversarial Network	66
