# Supplementary material for: Tumor Neoepitope-Based Vaccines: A Scoping Review on Current Predictive Computational Strategies
Source: Vaccines (Basel). 2024 Jul 24;12(8):836. doi: 10.3390/vaccines12080836 (PMC11360805; doi:10.3390/vaccines12080836)
Supplement: Supplementary file 1 [file vaccines-12-00836-s001.zip › Table S1 _ Reviewed articles excluded due to the adopted criterias - exclusion.pdf]

| | First exclusion | |
|---|---|---|
| | Article | Exclusion criteria |
| 1 | A Comprehensive in Silico Analysis for Identification of Immunotherapeutic Epitopes of HPV-18. | viral infection-mediated cancer |
| 2 | A comprehensive in silico analysis for identification of therapeutic epitopes in HPV16, 18, 31 and 45 oncoproteins | viral infection-mediated cancer |
| 3 | A conjoined universal helper epitope can unveil antitumor effects of a neoantigen vaccine targeting an MHC class I-restricted neoepitope | does not specify the use of neoepitope identification algorithms |
| 4 | A conserved subunit vaccine designed against SARS-CoV-2 variants showed evidence in neutralizing the virus | non-relevant subject |
| 5 | A Meta-analysis of Passive Immunization Studies Shows that Serum-Neutralizing Antibody Titer Associates with Protection against SHIV Challenge | non-relevant subject |
| 6 | A multiple peptides vaccine against COVID-19 designed from the nucleocapsid phosphoprotein (N) and Spike Glycoprotein (S) via the immunoinformatics approach | non-relevant subject |
| 7 | A Mutated Prostatic Acid Phosphatase (PAP) Peptide-Based Vaccine Induces PAP-Specific CD8+ T Cells with Ex Vivo Cytotoxic Capacities in HHDII/DR1 Transgenic Mice. | does not report the use of neoepitope identification algorithms and murine model |
| 8 | A Non-interventional Clinical Trial Assessing Immune Responses After Radiofrequency Ablation of Liver Metastases From Colorectal Cancer | non-relevant subject |
| 9 | A novel candidate HPV vaccine: MS2 phage VLP displaying a tandem HPV L2 peptide offers similar protection in mice to Gardasil-9 | viral infection-mediated cancer |
| 10 | A novel minigene scaffold for therapeutic cancer vaccines. | non-relevant subject |

| | Second exclusion | |
|---|---|---|
| | Article | Exclusion criteria |
| 1 | A combination of epitope prediction and molecular docking allows for good identification of MHC class I restricted T-cell epitopes | viral model |
| 2 | A high-throughput yeast display approach to profile pathogen proteomes for MHC-II binding | fungal model |
| 3 | A human B cell receptor epitope-based erbB-2 peptide (N: 163-182) with pan-reactivity to the T cells of Japanese breast cancer patients | analysis of specific genes |
| 4 | A large peptidome dataset improves HLA class I epitope prediction across most of the human population | does not present a relevant computational approach |
| 5 | A modified HLA-A*0201-restricted CTL epitope from human oncoprotein (hPEBP4) induces more efficient antitumor responses | analysis of specific genes |
| 6 | A neoepitope derived from a novel human germline APC gene mutation in familial adenomatous polyposis shows selective immunogenicity | analysis of specific genes |
| 7 | A New Epitope Selection Method: Application to Design a Multi-Valent Epitope Vaccine Targeting HRAS Oncogene in Squamous Cell Carcinoma. | analysis of specific genes |
| 8 | A novel in silico framework to improve MHC-I epitopes and break the tolerance to melanoma | wild-type peptide editing analysis |
| 9 | A pipeline for identification and validation of tumor-specific antigens in a mouse model of metastatic breast cancer | does not use human data |
| 10 | A Platform for Designing Genome-Based Personalized Immunotherapy or Vaccine against Cancer | does not use NGS data |

| | | |
|---|---|---|
| 11 | A Phase I/II trial comparing autologous dendritic cell vaccine pulsed either with personalized peptides (PEP-DC) or with tumor lysate (OC-DC) in patients with advanced high-grade ovarian serous carcinoma | does not specify the use of neoepitope identification algorithms |
| 12 | A Phase Ib Trial of Personalized Neoantigen Therapy Plus Anti-PD-1 in Patients with Advanced Melanoma, Non-small Cell Lung Cancer, or Bladder Cancer | does not specify the use of neoepitope identification algorithms |
| 13 | A SARS-CoV-2 Vaccination Strategy Focused on Population-Scale Immunity. | non-relevant subject |
| 14 | A Targeted LC-MS Strategy for Low-Abundant HLA Class-I-Presented Peptide Detection Identifies Novel Human Papillomavirus T-Cell Epitopes | viral infection-mediated cancer |
| 15 | A versatile papaya mosaic virus (PapMV) vaccine platform based on sortase-mediated antigen coupling | non-relevant subject |
| 16 | Abstracts from the 3rd International Genomic Medicine Conference (3rd IGMC 2015) : Jeddah, Kingdom of Saudi Arabia. 30 November - 3 December 2015. | non-relevant subject |
| 17 | Advances in personalized neoantigen vaccines for cancer immunotherapy | review |
| 18 | Advances in the study of HLA-restricted epitope vaccines | review |
| 19 | An analogue peptide from the cancer/testis antigen PASD1 induces CD8+ T cell responses against naturally processed peptide | does not specify the use of neoepitope identification algorithms |
| 20 | An anti-Gn glycoprotein antibody from a convalescent patient potently inhibits the infection of severe fever with thrombocytopenia syndrome virus | non-relevant subject |
| 21 | An antigenic space framework for understanding antibody escape of sars-cov-2 variants | non-relevant subject |

| | | |
|---|---|---|
| 11 | Advanced Pancreatic Cancer Patient Benefit From Personalized Neoantigen Nanovaccine Based Immunotherapy: A Case Report | case study, does not focus on in silico analysis |
| 12 | An efficient T-cell epitope discovery strategy using in silico prediction and the iTopia assay platform | analysis of specific genes |
| 13 | An immunogenic WT1-derived peptide that induces T cell response in the context of HLA-A*02:01 and HLA-A*24:02 molecules | analysis of specific genes |
| 14 | An in silico chimeric vaccine targeting breast cancer containing inherent adjuvant | analysis of specific genes |
| 15 | An oncofetal antigen, IMP-3-derived long peptides induce immune responses of both helper T cells and CTLs. | analysis of specific genes |
| 16 | Anti-tumor activity of a T-helper 1 multiantigen vaccine in a murine model of prostate cancer | does not present a relevant computational approach |
| 17 | Application of mass spectrometry-based MHC immunopeptidome profiling in neoantigen identification for tumor | review |
| 18 | Aspartate-β-hydroxylase induces epitope-specific T cell responses in hepatocellular carcinoma | analysis of specific genes |
| 19 | Biochemical and functional characterization of mutant KRAS epitopes validates this oncoprotein for immunological targeting | analysis of specific genes |
| 20 | Case Report: Pathological Complete Response in a Lung Metastasis of Phyllodes Tumor Patient Following Treatment Containing Peptide Neoantigen Nano-Vaccine. | case study, does not focus on in silico analysis |
| 21 | CD171 Multi-epitope peptide design based on immuno-informatics approach as a cancer vaccine candidate for glioblastoma | analysis of specific genes |

| | | |
|---|---|---|
| 22 | An Integrated Genomic, Proteomic, and Immunopeptidomic Approach to Discover Treatment-Induced Neoantigens | does not specify the use of neoepitope identification algorithms |
| 23 | Analysis of HLA A*02 Association with Vaccine Efficacy in the RV144 HIV-1 Vaccine Trial | non-relevant subject |
| 24 | Analysis of Major Histocompatibility Complex-Bound HIV Peptides Identified from Various Cell Types Reveals Common Nested Peptides and Novel T Cell Responses | non-relevant subject |
| 25 | Antibody isotype diversity against SARS-CoV-2 is associated with differential serum neutralization capacities | non-relevant subject |
| 26 | Antibody-dependent cellular cytotoxicity targeting CD4-inducible epitopes predicts mortality in HIV-infected infants | non-relevant subject |
| 27 | Antigen Choice Determines Vaccine-Induced Generation of Immunogenic versus Tolerogenic Dendritic Cells That Are Marked by Differential Expression of Pancreatic Enzymes | does not specify the use of neoepitope identification algorithms |
| 28 | Antigen processing and presentation in cancer immunotherapy | review |
| 29 | Antigenic Peptide Prediction From E6 and E7 Oncoproteins of HPV Types 16 and 18 for Therapeutic Vaccine Design Using Immunoinformatics and MD Simulation Analysis | viral infection-mediated cancer |
| 30 | Application of the pMHC array to characterise tumour antigen specific T cell populations in leukaemia patients at disease diagnosis | does not specify the use of neoepitope identification algorithms |
| 31 | ARTEMIS: A Novel Mass-Spec Platform for HLA-Restricted Self and Disease-Associated Peptide Discovery | does not specify the use of neoepitope identification algorithms |

| | | |
|---|---|---|
| 22 | CD8 T cell function and cross-reactivity explored by stepwise increased peptide-HLA versus TCR affinity | analysis of specific genes |
| 23 | Combined assessment of MHC binding and antigen abundance improves T cell epitope predictions. | viral model |
| 24 | Combining STING-based neoantigen-targeted vaccine with checkpoint modulators enhances antitumor immunity in murine pancreatic cancer | murine model |
| 25 | Comparative analysis of evolutionarily conserved motifs of epidermal growth factor receptor 2 (HER2) predicts novel potential therapeutic epitopes | analysis of specific genes |
| 26 | Computational Design of the Affinity and Specificity of a Therapeutic T Cell Receptor | does not present a relevant computation al approach |
| 27 | Computational prediction of vaccine potential epitopes and 3-dimensional structure of XAGE-1b for non-small cell lung cancer immunotherapy | analysis of specific genes |
| 28 | Cytotoxic T-lymphocyte elicited therapeutic vaccine candidate targeting cancer against MAGE-A11 carcinogenic protein | analysis of specific genes |
| 29 | Design of a new multi-epitope peptide vaccine for non-small cell Lung cancer via vaccinology methods: an in silico study | analysis of specific genes |
| 30 | Design of a Novel Recombinant Multi-Epitope Vaccine against Triple-Negative Breast Cancer | analysis of specific genes |
| 31 | Designing a Chimeric Vaccine Against Colorectal Cancer | analysis of specific genes |

| | | |
|---|---|---|
| 32 | Artificial Intelligence for COVID-19 Drug Discovery and Vaccine Development. | non-relevant subject |
| 33 | Autologous Dendritic Cells in Combination With Chemotherapy Restore Responsiveness of T Cells in Breast Cancer Patients: A Single-Arm Phase | does not specify the use of neoepitope identification algorithms |
| 34 | B-cell epitopes: Discontinuity and conformational analysis | non-relevant subject |
| 35 | Benchmarking predictions of MHC class I restricted T cell epitopes in a comprehensively studied model system | non-relevant subject |
| 36 | BepiTBR: T-B reciprocity enhances B cell epitope prediction. | non-relevant subject |
| 37 | Better epitope discovery, precision immune engineering, and accelerated vaccine design using Immunoinformatics tools | non-relevant subject |
| 38 | Bioinformatic prediction of potential T cell epitopes for SARS-Cov-2 | non-relevant subject |
| 39 | Bioinformatics analysis of HPV-68 E6 and E7 oncoproteins for designing a therapeutic epitope vaccine against HPV infection | viral infection-mediated cancer |
| 40 | Bladder cancer-associated cancer-testis antigen-derived long peptides encompassing both CTL and promiscuous HLA class II-restricted Th cell epitopes induced CD4+ T cells expressing converged T-cell receptor genes in vitro | viral infection-mediated cancer |
| 41 | BlockLogo: visualization of peptide and sequence motif conservation. | non-relevant subject |
| 42 | Breast cancer vaccination comes to age: impacts of bioinformatics | review |

| | | |
|---|---|---|
| 32 | Designing a Novel Multi-epitope T Vaccine for ``Targeting Protein for Xklp-2'' (TPX2) in Hepatocellular Carcinoma Based on Immunoinformatics Approach | analysis of specific genes |
| 33 | Designing a novel SOX9 based multi-epitope vaccine to combat metastatic triple-negative breast cancer using immunoinformatics approach | analysis of specific genes |
| 34 | Development of a peptide-based vaccine targeting TMPRSS2:ERG fusion-positive prostate cancer | analysis of specific genes |
| 35 | DockTope: A Web-based tool for automated pMHC-I modelling | does not present a relevant computation al approach |
| 36 | Empirical Evaluation of the Use of Computational HLA Binding as an Early Filter to the Mass Spectrometry-Based Epitope Discovery Workflow. | does not present a relevant computation al approach |
| 37 | Enhanced anti-colon cancer immune responses with modified eEF2-derived peptides | wild-type peptide editing analysis |
| 38 | Establishment of HLA-DR4 transgenic mice for the identification of CD4 + T cell epitopes of tumor-associated antigens | does not present a relevant computation al approach |
| 39 | Evaluating performance of existing computational models in predicting CD8+ T cell pathogenic epitopes and cancer neoantigens | does not present a relevant computation al approach |
| 40 | Experimental Study of Potential CD8+Trivalent Synthetic Peptides for Liver Cancer Vaccine Development Using Sprague Dawley Rat Models | does not address personalized therapy |
| 41 | Glypican-3-specific cytotoxic T lymphocytes induced by human leucocyte antigen-A*0201-restricted peptide effectively kill hepatocellular carcinoma cells in vitro. | analysis of specific genes |
| 42 | Graph-theoretical formulation of the generalized epitope-based vaccine design problem | does not present a relevant computation al approach |

| | | |
|---|---|---|
| 43 | Broadly neutralizing monoclonal antibodies for HIV prevention | non-relevant subject |
| 44 | Broadly Reactive Human CD8 T Cells that Recognize an Epitope Conserved between VZV, HSV and EBV | non-relevant subject |
| 45 | Can we predict mutant neoepitopes in human cancers for patient-specific vaccine therapy? | comment |
| 46 | Cancer immunotherapy | review |
| 47 | CD4 T Cells Specific for a Latency-Associated gamma-Herpesvirus Epitope Are Polyfunctional and Cytotoxic | viral infection-mediated cancer |
| 48 | CD8 and CD4 epitope predictions in RV144: No strong evidence of a T-cell driven sieve effect in HIV-1 Breakthrough sequences from trial participants | non-relevant subject |
| 49 | CD8 T-cell responses against the immunodominant Theileria parva peptide Tp249–59 are composed of two distinct populations specific for overlapping 11-mer and 10-mer epitopes | non-relevant subject |
| 50 | Cell death induced by cytotoxic CD8 + T cells is immunogenic and primes caspase-3-dependent spread immunity against endogenous tumor antigens | does not specify the use of neoepitope identification algorithms |
| 51 | Cellular therapy against public neoantigens | comment |
| 52 | Challenges targeting cancer neoantigens in 2021: a systematic literature review | review |
| 53 | Characterization of conserved and promiscuous human rhinovirus cd4 t cell epitopes | non-relevant subject |
| 54 | Chimerically fused antigen rich of overlapped epitopes from latent membrane protein 2 (LMP2) of Epstein-Barr virus as a potential vaccine and diagnostic agent | viral infection-mediated cancer |

| | | |
|---|---|---|
| 43 | High sensitivity of cancer exome-based CD8 T cell neo-antigen identification | does not use NGS data |
| 44 | HLA class I restricted epitopes prediction of common tumor antigens in white and East Asian populations: Implication on antigen selection for cancer vaccine design | specific gene analysis |
| 45 | HLA-A2-restricted Cytotoxic T Lymphocyte Epitopes from Human Hepsin as Novel Targets for Prostate Cancer Immunotherapy | analysis of specific genes |
| 46 | HLA-binding properties of tumor neoepitopes in humans | review |
| 47 | Hotspots' of Antigen Presentation Revealed by Human Leukocyte Antigen Ligandomics for Neoantigen Prioritization. | does not address personalized therapy |
| 48 | Identification and translational validation of novel mammaglobin-A CD8 T cell epitopes | analysis of specific genes |
| 49 | Identification of a novel HLA-A*02: 01-restricted cytotoxic T lymphocyte epitope derived from the EML4-ALK fusion gene | analysis of specific genes |
| 50 | Identification of a Promiscuous Epitope Peptide Derived from HSP70 | analysis of specific genes |
| 51 | Identification of an H2-Kb or H2-Db restricted and glypican-3-derived cytotoxic T-lymphocyte epitope peptide | analysis of specific genes |
| 52 | Identification of CD8+ T-cell epitope from multiple myeloma-specific antigen AKAP4 | analysis of specific genes |
| 53 | Identification of CDCA1-derived long peptides bearing both CD4+ and CD8+ T-cell epitopes: CDCA1-specific CD4+ T-cell immunity in cancer patients | analysis of specific genes |
| 54 | Identification of cross-reactive CD8+ T cell receptors with high functional avidity to a SARS-CoV-2 immunodominant epitope and its natural mutant variants. | viral model |

| | | |
|---|---|---|
| 55 | Classification of human leukocyte antigen (HLA) supertypes | book |
| 56 | Combining vaccines and immune checkpoint inhibitors to prime, expand, and facilitate effective tumor immunotherapy | review |
| 57 | Comparative immunogenicity and structural analysis of epitopes of different bacterial L-asparaginases | non-relevant subject |
| 58 | Comparison of personal and shared frameshift neoantigen vaccines in a mouse mammary cancer model | non-relevant subject |
| 59 | Complete mapping of mutations to the SARS-CoV-2 spike receptor-binding domain that escape antibody recognition. | non-relevant subject |
| 60 | Comprehensive viromewide antibody responses by systematic epitope scanning after hematopoietic cell transplantation | non-relevant subject |
| 61 | Computational analysis of anti-HIV-1 antibody neutralization panel data to identify potential functional epitope residues | non-relevant subject |
| 62 | Computational Design and Analysis of a Multi-epitope Against Influenza A | non-relevant subject |
| 63 | Computational identification, characterization and validation of potential antigenic peptide vaccines from hrHPVs E6 proteins using immunoinformatics and computational systems biology approaches | viral infection-mediated cancer |
| 64 | Computational Prediction and Validation of Tumor-Associated Neoantigens | review |
| 65 | Computational Screening of the Human TF-Glycome Provides a Structural Definition for the Specificity of Anti-Tumor Antibody JAA-F11 | non-relevant subject |
| 66 | Computational tools for modern vaccine development | review |

| | | |
|---|---|---|
| 55 | Identification of glypican-3-derived long peptides activating both CD8+ and CD4+ T cells; prolonged overall survival in cancer patients with Th cell response. | analysis of specific genes |
| 56 | Identification of HLA-A*1101-restricted cytotoxic T lymphocyte epitopes derived from epidermal growth factor pathway substrate number 8 | analysis of specific genes |
| 57 | Identification of HLA-A24-restricted CD8 + cytotoxic T-cell epitopes derived from mammaglobin-A, a human breast cancer-associated antigen | analysis of specific genes |
| 58 | Identification of human leukemia antigen A*0201-restricted epitopes derived from epidermal growth factor pathway substrate number 8 | analysis of specific genes |
| 59 | Identification of Immunogenic MHC Class II Human HER3 Peptides that Mediate Anti-HER3 CD4 Th1 Responses and Potential Use as a Cancer Vaccine | analysis of specific genes |
| 60 | Identification of Immunogenic MHC Class II Human HER3 Peptides that Mediate Anti-HER3 CD4 Th1 Responses and Potential Use as a Cancer Vaccine | analysis of specific genes |
| 61 | Identification of Neoantigens in Two Murine Gastric Cancer Cell Lines Leading to the Neoantigen-Based Immunotherapy | murine model |
| 62 | Identification of new HLA-A*0201-restricted cytotoxic T lymphocyte epitopes from neuritin | analysis of specific genes |
| 63 | Identification of Prostate-Specific G-Protein Coupled Receptor as a Tumor Antigen Recognized by CD8+ T Cells for Cancer Immunotherapy | analysis of specific genes |
| 64 | Identification of Special AT-Rich Sequence Binding Protein 1 as a Novel Tumor Antigen Recognized by CD8+ T Cells: Implication for Cancer Immunotherapy | analysis of specific genes |
| 65 | Identifying T Cell Receptors from High-Throughput Sequencing: Dealing with Promiscuity in TCRα and TCRβ Pairing | does not present a relevant computational approach |
| 66 | Immunogenicity of Del19 EGFR mutations in Chinese patients affected by lung adenocarcinoma | analysis of specific genes |

| | | |
|---|---|---|
| 67 | Computational Tools for the Identification and Interpretation of Sequence Motifs in Immunopeptidomes | review |
| 68 | Computationally Optimized SARS-CoV-2 MHC Class I and II Vaccine Formulations Predicted to Target Human Haplotype Distributions | non-relevant subject |
| 69 | Computer-Aided Design of an Epitope-Based Vaccine against Epstein-Barr Virus | viral infection-mediated cancer |
| 70 | Computer-aided prediction and design of IL-6 inducing peptides: IL-6 | non-relevant subject |
| 71 | Construction of A Synthetic Gene Encoding the Multi-Epitope of Toxoplasma gondii and Demonstration of the Relevant Recombinant Protein Production: A Vaccine Candidate. | non-relevant subject |
| 72 | Construction, expression, and in vitro assembly of virus-like particles of L1 protein of human papillomavirus type 52 in Escherichia coli BL21 DE3 | viral infection-mediated cancer |
| 73 | Cross-sectional analysis of CD8 T cell immunity to human herpesvirus 6B | non-relevant subject |
| 74 | Current perspectives in cancer immunotherapy | review |
| 75 | Current tools for predicting cancer-specific T cell immunity. | review |
| 76 | Cutting Edge: An Antibody Recognizing Ancestral Endogenous Virus Glycoproteins Mediates Antibody-Dependent Cellular Cytotoxicity on HIV-1–Infected Cells | non-relevant subject |
| 77 | Cytotoxic T lymphocytes targeting a conserved SARS-CoV-2 spike epitope are efficient serial killers | non-relevant subject |
| 78 | Cytotoxic T-Cell-Based Vaccine against SARS-CoV-2: A Hybrid Immunoinformatic Approach. | non-relevant subject |

| | | |
|---|---|---|
| 67 | Immunoinformatics Approach to Design T-cell Epitope-Based Vaccine | viral model |
| 68 | Implementation of Vaccinomics and In-Silico Approaches to Construct Multimeric Based Vaccine Against Ovarian Cancer | analysis of specific genes |
| 69 | In silico analysis, molecular docking, molecular dynamic, cloning, expression and purification of chimeric protein in colorectal cancer treatment | analysis of specific genes |
| 70 | In silico and cell-based analyses reveal strong divergence between prediction and observation of T-cell-recognized tumor antigen T-cell epitopes | does not present a relevant computation al approach |
| 71 | In silico approach in designing a novel multi-epitope vaccine candidate against non-small cell lung cancer with overexpressed G protein-coupled receptor 56 | analysis of specific genes |
| 72 | In silico design and evaluation of PRAME+FliC δD2D3 as a new breast cancer vaccine candidate | analysis of specific genes |
| 73 | In silico design of a triple-negative breast cancer vaccine by targeting cancer testis antigens | analysis of specific genes |
| 74 | In silico design of discontinuous peptides representative of b and t-cell epitopes from her2-ecd as potential novel cancer peptide vaccines | analysis of specific genes |
| 75 | In silico evaluation of PLAC1-flic as a chimeric vaccine against breast cancer | analysis of specific genes |
| 76 | In silico prediction of B cell epitopes of the extracellular domain of insulin-like growth factor-1 receptor. | analysis of specific genes |
| 77 | In silico-guided sequence modifications of K-ras epitopes improve immunological outcome against G12V and G13D mutant KRAS antigens. | wild-type peptide editing analysis |
| 78 | Integrating CD4+ T cell help for therapeutic cancer vaccination in a preclinical head and neck cancer model | analysis of specific genes |

| | | |
|---|---|---|
| 79 | De novo protein design enables the precise induction of RSV-neutralizing antibodies | non-relevant subject |
| 80 | Deconvoluting virome-wide antibody epitope reactivity profiles | non-relevant subject |
| 81 | Deep learning boosts sensitivity of mass spectrometry-based immunopeptidomics | non-relevant subject |
| 82 | Deep sequencing in pre- and clinical vaccine research | review |
| 83 | Deimmunization of flagellin for repeated administration as a vaccine | non-relevant subject |
| 84 | Deimmunizing substitutions in Pseudomonas exotoxin domain III perturb antigen processing without eliminating T-cell epitopes | non-relevant subject |
| 85 | Delineating surface epitopes of lyme disease pathogen targeted by highly protective antibodies of New Zealand white rabbits | non-relevant subject |
| 86 | Design of a multi-epitope protein vaccine against herpes simplex virus, human papillomavirus and Chlamydia trachomatis as the main causes of sexually transmitted diseases | viral infection-mediated cancer |
| 87 | Design of a multi-epitope vaccine against cervical cancer using immunoinformatics approaches | viral infection-mediated cancer |
| 88 | Designing a multi-epitope based vaccine to combat Kaposi Sarcoma utilizing immunoinformatics approach | viral infection-mediated cancer |
| 89 | Designing a multi-epitopic vaccine against the enterotoxigenic Bacteroides fragilis based on immunoinformatics approach | non-relevant subject |
| 90 | Designing a sars-cov-2 t-cell-inducing vaccine for high-risk patient groups | non-relevant subject |

| | | |
|---|---|---|
| 79 | Key Parameters of Tumor Epitope Immunogenicity Revealed Through a Consortium Approach Improve Neoantigen Prediction | does not present a relevant computational approach |
| 80 | Linear and conformational B cell epitope prediction of the HER 2 ECD-subdomain III by in silico methods. | analysis of specific genes |
| 81 | Metadherin peptides containing CD4+ and CD8+ T cell epitopes as a therapeutic vaccine candidate against cancer | analysis of specific genes |
| 82 | MHC class I loaded ligands from breast cancer cell lines: A potential HLA-I-typed antigen collection | does not use NGS data |
| 83 | Mismatch Repair Deficiency Drives Durable Complete Remission by Targeting Programmed Death Receptor 1 in a Metastatic Luminal Breast Cancer Patient. | does not present a relevant computational approach |
| 84 | MUC1 glycopeptide epitopes predicted by computational glycomics | analysis of specific genes |
| 85 | Mutant MHC class II epitopes drive therapeutic immune responses to cancer | murine model |
| 86 | Novel peptide-based vaccine targeting heat shock protein 90 induces effective antitumor immunity in a HER2+ breast cancer murine model | analysis of specific genes |
| 87 | Novel Predicted B-Cell Epitopes of PSMA for Development of Prostate Cancer Vaccine | analysis of specific genes |
| 88 | Optimized polyepitope neoantigen DNA vaccines elicit neoantigen-specific immune responses in preclinical models and in clinical translation | murine model |
| 89 | Pan-cancer analysis of neoepitopes | does not present a relevant computational approach |
| 90 | Peptide FLNPDVLDI of heparanase is a novel HLA-A2-restricted CTL epitope and elicits potent immunological antitumor effects in vitro with an 8-branched-design | analysis of specific genes |

| | | |
|---|---|---|
| 91 | Designing of CD8 + and CD8 + -overlapped CD4 + epitope vaccine by targeting late and early proteins of human papillomavirus | viral infection-mediated cancer |
| 92 | Designing of multi-epitope chimeric vaccine using immunoinformatic platform by targeting oncogenic strain HPV 16 and 18 against cervical cancer | viral infection-mediated cancer |
| 93 | Determination of B- And T- cell epitopes for Helicobacter pylori cagPAI: An in silico approach | non-relevant subject |
| 94 | Development of a novel immunoproteasome digestion assay for synthetic long peptide vaccine design | non-relevant subject |
| 95 | Development of a novel, quantitative protein microarray platform for the multiplexed serological analysis of autoantibodies to cancer-testis antigens | non-relevant subject |
| 96 | Development of an epitope panel for consistent identification of antigen-specific T-cells in humans | does not specify the use of neoepitope identification algorithms |
| 97 | Development of Envelope Protein Antigens To Serologically Differentiate Zika Virus Infection from Dengue Virus Infection | non-relevant subject |
| 98 | Development of thyroglobulin antibodies after GVAX immunotherapy is associated with prolonged survival | does not specify the use of neoepitope identification algorithms |
| 99 | Development of tumour peptide vaccines: From universalization to personalization | review |
| 100 | Early prediction of antigenic transitions for influenza A/H3N2 | non-relevant subject |
| 101 | Editing the immunopeptidome of melanoma cells using a potent inhibitor of endoplasmic reticulum aminopeptidase 1 (ERAP1) | does not specify the use of neoepitope identification algorithms |

| | | |
|---|---|---|
| 91 | Population-level distribution and putative immunogenicity of cancer neoepitopes | does not use NGS data |
| 92 | Predicting T cell recognition of MHC class I restricted neoepitopes | does not use NGS data |
| 93 | Presence of antigen-specific somatic allelic mutations and splice variants do not predict for immunological response to genetic vaccination. | does not present a relevant computational approach |
| 94 | Recurrent Frameshift Neoantigen Vaccine Elicits Protective Immunity With Reduced Tumor Burden and Improved Overall Survival in a Lynch Syndrome Mouse Model | murine model |
| 95 | Residue substitution enhances the immunogenicity of neoepitopes from gastric cancers | wild-type peptide editing analysis |
| 96 | Robust prediction of HLA class II epitopes by deep motif deconvolution of immunopeptidomes | closed |
| 97 | Role of in silico structural modeling in predicting immunogenic neoepitopes for cancer vaccine development | murine model |
| 98 | Screening of Human Epidermal Growth Factor Receptor 2 (HER2) Extracellular Domain for Potential Epitopes by Using Immuno-informatics Tools | analysis of specific genes |
| 99 | Shared Immunogenic Poly-Epitope Frameshift Mutations in Microsatellite Unstable Tumors | does not present a relevant computational approach |
| 100 | Structural Analysis and Epitope Prediction of MHC Class-1-Chain Related Protein-A for Cancer Vaccine Development. | analysis of specific genes |
| 101 | Structural Features of Antibody-Peptide Recognition | does not present a relevant computational approach |

| | | |
|---|---|---|
| 102 | Elucidating the immunological effects of 5-azacytidine treatment in patients with myelo-dysplastic syndrome and identifying new conditional ligands and T-cell epitopes of relevance in melanoma | review |
| 103 | Engineering $\alpha$-fetoprotein-based gene vaccines to prevent and treat hepatocellular carcinoma: Review and future prospects | review |
| 104 | Enhancement of specific T-lymphocyte responses by monocyte-derived dendritic cells pulsed with E2 protein of human papillomavirus 16 and human p16INK4A. | viral infection-mediated cancer |
| 105 | Enhancing antitumor immune responses by optimized combinations of cell-penetrating peptide-based vaccines and adjuvants | does not specify the use of neoepitope identification algorithms |
| 106 | Epitope design of L1 protein for vaccine production against Human Papilloma Virus types 16 and 18. | viral infection-mediated cancer |
| 107 | Evaluation of a novel monoclonal antibody against tumor-associated MUC1 for diagnosis and prognosis of breast cancer | does not specify the use of neoepitope identification algorithms |
| 108 | Evaluation of human antibody responses to keyhole limpet hemocyanin on a carbohydrate microarray | non-relevant subject |
| 109 | Exploring SARS-COV-2 structural proteins to design a multi-epitope vaccine using immunoinformatics approach: An in silico study | non-relevant subject |
| 110 | Exploring the out of sight antigens of SARS-CoV-2 to design a candidate multi-epitope vaccine by utilizing immunoinformatics approaches | non-relevant subject |
| 111 | Exploring the Papillomaviral Proteome to Identify Potential Candidates for a Chimeric Vaccine against Cervix Papilloma Using Immunomics and Computational Structural Vaccinology | viral infection-mediated cancer |

| | | |
|---|---|---|
| 102 | Systematically benchmarking peptide-MHC binding predictors: From synthetic to naturally processed epitopes | does not use mutation data |
| 103 | TANTIGEN 2.0: a knowledge base of tumor T cell antigens and epitopes | database construction |
| 104 | Targeting Tumor Markers with Antisense Peptides: An Example of Human Prostate Specific Antigen | analysis of specific genes |
| 105 | TCR contact residue hydrophobicity is a hallmark of immunogenic CD8(+) T cell epitopes | viral model |
| 106 | The Cancer Epitope Database and Analysis Resource: A Blueprint for the Establishment of a New Bioinformatics Resource for Use by the Cancer Immunology Community | does not present a relevant computational approach |
| 107 | Tumor neoantigens: Building a framework for personalized cancer immunotherapy | review |
| 108 | Two novel squamous cell carcinoma antigen-derived HLA-A <sup>*</sup>0201-binding peptides induce in vitro and in vivo CD8 <sup>+</sup> cytotoxic T lymphocyte responses | analysis of specific genes |
| 109 | USMPep: universal sequence models for major histocompatibility complex | viral model |
| 110 | Vaccine candidate designed against carcinoembryonic antigen-related cell adhesion molecules using immunoinformatics tools | analysis of specific genes |

| | | |
|---|---|---|
| 112 | Fine epitope signature of antibody neutralization breadth at the HIV-1 envelope CD4-binding site | non-relevant subject |
| 113 | Footprints of antigen processing boost MHC class II natural ligand predictions | does not specify the use of neoepitope identification algorithms |
| 114 | Frequency of Interferon-Resistance Conferring Substitutions in Amino Acid Positions 70 and 91 of Core Protein of the Russian HCV 1b Isolates Analyzed in the T-Cell Epitopic Context | non-relevant subject |
| 115 | Functional Implications of the Binding Mode of a Human Conformation-Dependent V2 Monoclonal Antibody against HIV | non-relevant subject |
| 116 | Gag and env conserved element CE DNA vaccines elicit broad cytotoxic T cell responses targeting subdominant epitopes of HIV and SIV Able to recognize virus-infected cells in macaques | non-relevant subject |
| 117 | Genetic and Structural Analysis of SARS-CoV-2 Spike Protein for Universal Epitope Selection | non-relevant subject |
| 118 | Genetic variability in minor capsid protein (L2 gene) of human papillomavirus type 16 among Indian women | viral infection-mediated cancer |
| 119 | Genetic variability of E6 and E7 genes of human papillomavirus type 58 in Jingzhou, Hubei Province of central China | viral infection-mediated cancer |
| 120 | Genetically barcoded SIV reveals the emergence of escape mutations in multiple viral lineages during immune escape | non-relevant subject |
| 121 | Genome Diversity of Epstein-Barr Virus from Multiple Tumor Types and Normal Infection | viral infection-mediated cancer |
| 122 | Getting Personal with Neoantigen-Based Therapeutic Cancer Vaccines | review |
| 123 | Glycoconjugate Nanoparticle-Based Systems in Cancer Immunotherapy: Novel Designs and Recent Updates | review |
| 124 | Hepatitis C virus E2 envelope glycoprotein core structure | review |

| | | |
|---|---|---|
| 125 | High immunogenicity of the human leukocyte antigen peptidomes of melanoma tumor cells | non-relevant subject |
| 126 | High-density peptide arrays help to identify linear immunogenic B-cell epitopes in individuals naturally exposed to malaria infection | non-relevant subject |
| 127 | HIV-1 Vaccine-Induced T-Cell Reponses Cluster in Epitope Hotspots that Differ from Those Induced in Natural Infection with HIV-1 | non-relevant subject |
| 128 | HLA-A2-Restricted Epitopes Identified from MTA1 Could Elicit Antigen-Specific Cytotoxic T Lymphocyte Response | non-relevant subject |
| 129 | HLA-DQB1*06 and breadth of Nef core region-specific T-cell response are associated with slow disease progression in antiretroviral therapy-naive Chinese HIV-1 subtype B patients | non-relevant subject |
| 130 | HPV-Associated Tumor Eradication by Vaccination with Synthetic Short Peptides and Particle-Forming Liposomes | viral infection-mediated cancer |
| 131 | HPV16 E6-specific T cell response and HLA-A alleles are related to the prognosis of patients with cervical cancer. | viral infection-mediated cancer |
| 132 | HPV16-E7 Protein T Cell Epitope Prediction and Global Therapeutic Peptide Vaccine Design Based on Human Leukocyte Antigen Frequency: An In-Silico Study. | viral infection-mediated cancer |
| 133 | HPVdb: A data mining system for knowledge discovery in human papillomavirus with applications in Tcell immunology and vaccinology | viral infection-mediated cancer |
| 134 | HPVMD-C: a disease-based mutation database of human papillomavirus in China | viral infection-mediated cancer |
| 135 | HPVomics: An integrated resource for the human papillomavirus epitome and therapeutics | viral infection-mediated cancer |
| 136 | Human immune responses to H. Pylori HLA class II epitopes identified by immunoinformatic methods | non-relevant subject |

| | | |
|---|---|---|
| 137 | Identification and characterization of enhancer agonist human cytotoxic T-cell epitopes of the human papillomavirus type 16 (HPV16) E6/E7 | viral infection-mediated cancer |
| 138 | Identification and validation of immunogenic potential of India specific HPV-16 variant constructs: In-silico &in-vivo insight to vaccine development | viral infection-mediated cancer |
| 139 | Identification and visualization of multidimensional antigen-specific T-cell populations in polychromatic cytometry data | non-relevant subject |
| 140 | Identification B and T-Cell epitopes and functional exposed amino acids of S protein as a potential vaccine candidate against SARS-CoV-2/COVID-19 | non-relevant subject |
| 141 | Identification of cross-reactive CD8+ T cell receptors with high functional avidity to a SARS-CoV-2 immunodominant epitope and its natural mutant variants. | non-relevant subject |
| 142 | Identification of Effective Subdominant Anti-HIV-1 CD8+ T Cells Within Entire Post-infection and Post-vaccination Immune Responses | non-relevant subject |
| 143 | Identification of evolutionarily stable functional and immunogenic sites across the SARS-CoV-2 proteome and greater coronavirus family | non-relevant subject |
| 144 | Identification of HLA-A*24:02-Restricted CTL Candidate Epitopes Derived from the Nonstructural Polyprotein 1a of SARS-CoV-2 and Analysis of Their Conservation Using the Mutation Database of SARS-CoV-2 Variants | non-relevant subject |
| 145 | Identification of Human Anti-HIV gp160 Monoclonal Antibodies That Make Effective Immunotoxins | viral infection-mediated cancer |
| 146 | Identification of human leucocyte antigen (HLA)-A*0201-restricted cytotoxic T lymphocyte epitopes derived from HLA-DOβ as a novel target for multiple myeloma | does not specify the use of neoepitope identification algorithms |

| | | |
|---|---|---|
| 147 | Identification of Naturally Processed Epitope Region Using Artificial APC Expressing a Single HLA Class I Allotype and mRNA of HCMV pp65 Antigen Fragments. | non-relevant subject |
| 148 | Identification of promiscuous HPV16-derived T helper cell epitopes for therapeutic HPV vaccine design | viral infection-mediated cancer |
| 149 | Identification of Relevant Conformational Epitopes on the HER2 Oncoprotein by Using Large Fragment Phage Display (LFPD) | does not specify the use of neoepitope identification algorithms |
| 150 | Identification of SARS-CoV-2 Nucleocapsid and Spike T-Cell Epitopes for Assessing T-Cell Immunity | non-relevant subject |
| 151 | Identification of SARS-CoV-2 Vaccine Epitopes Predicted to Induce Long-Term Population-Scale Immunity. | non-relevant subject |
| 152 | Identification of variants and therapeutic epitopes in HPV-33/HPV-58 E6 and E7 in Southwest China | viral infection-mediated cancer |
| 153 | Immune epitopes identification and designing of a multi-epitope vaccine against bovine leukemia virus: a molecular dynamics and immune simulation approaches | non-relevant subject |
| 154 | Immune recognition of tumor-associated mucin MUC1 is achieved by a fully synthetic aberrantly glycosylated MUC1 tripartite vaccine | non-relevant subject |
| 155 | Immunization with short peptide particles reveals a functional CD8 + T-cell neoepitope in a murine renal carcinoma model | non-relevant subject |
| 156 | Immunobiological effects of gemcitabine and capecitabine combination chemotherapy in advanced pancreatic ductal adenocarcinoma | non-relevant subject |
| 157 | Immunogenic T cell epitopes of SARS-CoV-2 are recognized by circulating memory and naive CD8 T cells of unexposed individuals | non-relevant subject |

| | | |
|---|---|---|
| 158 | Immunoinformatic Design of a Multivalent Peptide Vaccine Against Mucormycosis: Targeting FTR1 Protein of Major Causative Fungi | non-relevant subject |
| 159 | Immunoinformatics and molecular dynamics approaches: Next generation vaccine design against West Nile virus | non-relevant subject |
| 160 | Immunoinformatics and Structural Analysis for Identification of Immunodominant Epitopes in SARS-CoV-2 as Potential Vaccine Targets. | non-relevant subject |
| 161 | Immunoinformatics Approach to Design a Novel Epitope-Based Oral Vaccine Against Helicobacter pylori | non-relevant subject |
| 162 | Immunoinformatics guided design of a next generation epitope-based vaccine against Kaposi Sarcoma | viral infection-mediated cancer |
| 163 | Immunoinformatics- and Bioinformatics-Assisted Computational Designing of a Novel Multiepitopes Vaccine Against Cancer-Causing Merkel Cell Polyomavirus | viral infection-mediated cancer |
| 164 | Immunoinformatics: Predicting Peptide-MHC Binding | book |
| 165 | Immunopathologic stratification of colorectal cancer for checkpoint blockade immunotherapy | does not specify the use of neoepitope identification algorithms |
| 166 | Immunopeptidomic analysis of influenza A virus infected human tissues identifies internal proteins as a rich source of HLA ligands | non-relevant subject |
| 167 | Improved MHC II epitope prediction - a step towards personalized medicine | non-relevant subject |
| 168 | Improvement of epitope prediction using peptide sequence descriptors and machine learning | does not specify the use of neoepitope identification algorithms |
| 169 | Improving Neutralization Potency and Breadth by Combining Broadly Reactive HIV-1 Antibodies Targeting Major Neutralization Epitopes | non-relevant subject |

| | | |
|---|---|---|
| 170 | In silico analysis of epitope-based vaccine candidate against tuberculosis using reverse vaccinology | non-relevant subject |
| 171 | In Silico Analysis Predicts a Limited Impact of SARS-CoV-2 Variants on CD8 T Cell Recognition | non-relevant subject |
| 172 | In silico CD4+T-cell multiepitope prediction and HLA distribution analysis for Marburg Virus-A strategy for vaccine designing | non-relevant subject |
| 173 | In silico design of a multi-epitope vaccine against HPV16/18 | viral infection-mediated cancer |
| 174 | In silico detection of SARS-CoV-2 specific B-cell epitopes and validation in ELISA for serological diagnosis of COVID-19 | non-relevant subject |
| 175 | In Silico Methods in Antibody | review |
| 176 | In silico model estimates the clinical trial outcome of cancer vaccines | meta-analysis |
| 177 | In silico prediction of cancer immunogens: Current state of the art | review |
| 178 | In silico selection of functionally important proteins from the mialome of Ornithodoros erraticus ticks and assessment of their protective efficacy as vaccine targets | non-relevant subject |
| 179 | In silico/In vivo analysis of high-risk papillomavirus L1 and L2 conserved sequences for development of cross-subtype prophylactic vaccine | viral infection-mediated cancer |
| 180 | In vivo and in vitro immunogenicity of novel MHC class I presented epitopes to confer protective immunity against chronic HTLV-1 infection | viral infection-mediated cancer |
| 181 | Induction of HER2 immunity in outbred domestic cats by DNA electrovaccination | non-relevant subject |
| 182 | Infectious disease mRNA vaccines and a review on epitope prediction for vaccine design | review |

| | | |
|---|---|---|
| 183 | Integrated Multi-omics, Virtual Screening and Molecular Docking Analysis of Methicillin-Resistant Staphylococcus aureus USA300 for the Identification of Potential Therapeutic Targets: An In-Silico Approach. | non-relevant subject |
| 184 | Japanese Encephalitis Virus Vaccination Elicits Cross-Reactive HLA-Class I-Restricted CD8 T Cell Response Against Zika Virus Infection | non-relevant subject |
| 185 | Landscape and selection of vaccine epitopes in SARS-CoV-2 | non-relevant subject |
| 186 | Leishmaniac Quest for Developing a Novel Vaccine Platform. Is a Roadmap for Its Advances Provided by the Mad Dash to Produce Vaccines for COVID-19? | non-relevant subject |
| 187 | Limited Predictability of Amino Acid Substitutions in Seasonal Influenza Viruses | non-relevant subject |
| 188 | Long-term survival correlates with immunological responses in renal cell carcinoma patients treated with mRNA-based immunotherapy. | does not specify the use of neoepitope identification algorithms |
| 189 | Lymphocyte-polarized dendritic cells are highly effective in inducing tumor-specific CTLs | non-relevant subject |
| 190 | Machine Learning for Cancer Immunotherapies Based on Epitope Recognition by T Cell Receptors. | review |
| 191 | Mapping Polyclonal HIV-1 Antibody Responses via Next-Generation Neutralization Fingerprinting | non-relevant subject |
| 192 | Melanoma-specific antigen-associated antitumor antibody reactivity as an immune-related biomarker for targeted immunotherapies. | does not specify the use of neoepitope identification algorithms |
| 193 | Metaviromic identification of discriminative genomic features in SARS-CoV-2 using machine learning. | non-relevant subject |
| 194 | MHC class I antigen presentation and implications for developing a new generation of therapeutic vaccines | comment |
| 195 | Mining the mutanome: developing highly personalized Immunotherapies based on mutational analysis of tumors. | non-relevant subject |

| | | |
|---|---|---|
| 196 | Molecular cloning, expression, IgE binding activities and in silico epitope prediction of per a 9 allergens of the American cockroach | non-relevant subject |
| 197 | MRKAD5 HIV-1 Gag/Pol/Nef vaccine-induced T-cell responses inadequately predict distance of breakthrough HIV-1 sequences to the vaccine or viral load | non-relevant subject |
| 198 | mRNA vaccine–induced neoantigen-specific T cell immunity in patients with gastrointestinal cancer | does not specify the use of neoepitope identification algorithms |
| 199 | MUCIN-4 (MUC4) is a novel tumor antigen in pancreatic cancer immunotherapy | review |
| 200 | Multi-antigen Vaccination With Simultaneous Engagement of the OX40 Receptor Delays Malignant Mesothelioma Growth and Increases Survival in Animal Models. | non-relevant subject |
| 201 | Multi-epitope vaccines: A promising strategy against tumors and viral infections | review |
| 202 | Multi-Epitope-Based Vaccines for Colon Cancer Treatment and Prevention | does not specify the use of neoepitope identification algorithms |
| 203 | Multiple-Allele MHC Class II Epitope Engineering by a Molecular Dynamics-Based Evolution Protocol | non-relevant subject |
| 204 | N-glycosylation of Colorectal Cancer Tissues | non-relevant subject |
| 205 | Naturally occurring capsid protein variants L1 of human papillomavirus | viral infection-mediated cancer |
| 206 | Neoantigen Targeting-Dawn of a New Era in Cancer Immunotherapy? | review |
| 207 | Neoantigens in Hematological Malignancies—Ultimate Targets for Immunotherapy? | review |
| 208 | Neoantigens in immunotherapy and personalized vaccines: Implications for head and neck squamous cell carcinoma | review |
| 209 | Neoepitopes-based vaccines: challenges and perspectives | review |

| | | |
|---|---|---|
| 210 | New tools for MHC research from machine learning and predictive algorithms to the tumour immunopeptidome | comment |
| 211 | Oncolytic virotherapy induced CSDE1 neo-antigenesis restricts VSV replication but can be targeted by immunotherapy | non-relevant subject |
| 212 | Pan-genome analysis of human gastric pathogen H. pylori: Comparative genomics and pathogenomics approaches to identify regions associated with pathogenicity and prediction of potential core therapeutic targets | non-relevant subject |
| 213 | Peptide targeted by human antibodies associated with HIV vaccine-associated protection assumes a dynamic $\alpha$-helical structure | non-relevant subject |
| 214 | Peptides Designed To Spatially Depict the Epstein-Barr Virus Major Virion Glycoprotein gp350 Neutralization Epitope Elicit Antibodies That Block Virus-Neutralizing Antibody 72A1 Interaction with the Native gp350 Molecule | viral infection-mediated cancer |
| 215 | Personal neoantigen cancer vaccines: A road not fully paved | review |
| 216 | Personal neoantigen vaccines induce persistent memory T cell responses and epitope spreading in patients with melanoma | does not specify the use of neoepitope identification algorithms |
| 217 | Personalized cancer immunotherapy using Systems Medicine approaches | review |
| 218 | Phage-DMS: A Comprehensive Method for Fine Mapping of Antibody Epitopes. | non-relevant subject |
| 219 | Pharmacokinetics and predicted neutralisation coverage of VRC01 in HIV-uninfected participants of the Antibody Mediated Prevention (AMP) trials | non-relevant subject |
| 220 | Phase II clinical trial using novel peptide cocktail vaccine as a postoperative adjuvant treatment for surgically resected pancreatic cancer patients | does not specify the use of neoepitope identification algorithms |

| | | |
|---|---|---|
| 221 | Phenotype of p53 wild-type epitope-specific T cells in the circulation of patients with head and neck cancer | does not specify the use of neoepitope identification algorithms |
| 222 | Physical detection of influenza A epitopes identifies a stealth subset on human lung epithelium evading natural CD8 immunity | non-relevant subject |
| 223 | Plasma immune analytes in patients with epithelial ovarian cancer | does not specify the use of neoepitope identification algorithms |
| 224 | Point-of-View T-cell Immunity Directed toward Human Tumors | review |
| 225 | Poor correlation between T-cell activation assays and HLA-DR binding prediction algorithms in an immunogenic fragment of Pseudomonas exotoxin A | non-relevant subject |
| 226 | Pre-vaccination frequencies of Th17 cells correlate with vaccine-induced T-Cell responses to survivin-derived peptide epitopes | non-relevant subject |
| 227 | Predicting the broadly neutralizing antibody susceptibility of the HIV reservoir | non-relevant subject |
| 228 | Predicting the Mutating Distribution at Antigenic Sites of the Influenza Virus | non-relevant subject |
| 229 | Prediction and identification of CD4+ T cell epitope for the protective antigens of Mycobacterium tuberculosis | non-relevant subject |
| 230 | Prediction and identification of human leukocyte antigen-A2-restricted cytotoxic T lymphocyte epitope peptides from the human papillomavirus 58 E7 protein. | viral infection-mediated cancer |
| 231 | Prediction of Epitope-Associated TCR by Using Network Topological Similarity Based on Deepwalk | non-relevant subject |
| 232 | Prediction of promiscuous epitopes in the e6 protein of three high risk human papilloma viruses: A computational approach | viral infection-mediated cancer |

| | | |
|---|---|---|
| 233 | Prediction of the binding interface between monoclonal antibody m102.4 and Nipah attachment glycoprotein using structure-guided alanine scanning and computational docking | non-relevant subject |
| 234 | Prediction of the efficacy of immunotherapy by measuring the integrity of cell-free DNA in plasma in colorectal cancer | non-relevant subject |
| 235 | Prediction, dynamics, and visualization of antigenic phenotypes of seasonal influenza viruses | non-relevant subject |
| 236 | Prioritization of SARS-CoV-2 epitopes using a pan-HLA and global population inference approach. | non-relevant subject |
| 237 | Probable HLA-mediated immunoediting of JAK2 V617F-driven oncogenesis | does not specify the use of neoepitope identification algorithms |
| 238 | Proof of concept study with an HER-2 mimotope anticancer vaccine deduced from a novel AAV-mimotope library platform | does not specify the use of neoepitope identification algorithms |
| 239 | Prophylactic and therapeutic adenoviral vector-based multivirus-specific T-cell immunotherapy for transplant patients. | non-relevant subject |
| 240 | Protein structure shapes immunodominance in the CD4 T cell response to yellow fever vaccination | non-relevant subject |
| 241 | Quantifiable predictive features define epitope-specific T cell receptor repertoires | non-relevant subject |
| 242 | Quantitative analysis and clonal characterization of T-cell receptor β repertoires in patients with advanced non-small cell lung cancer treated with cancer vaccine | non-relevant subject |
| 243 | Quantitative and qualitative impairments in dendritic cell subsets of patients with ovarian or prostate cancer | non-relevant subject |
| 244 | Radiotherapy-exposed CD8+ and CD4+ neoantigens enhance tumor control | non-relevant subject |

| | | |
|---|---|---|
| 245 | Response of high-risk of recurrence/progression bladder tumours expressing sialyl-Tn and sialyl-6-T to BCG immunotherapy | non-relevant subject |
| 246 | Risk of rapid evolutionary escape from biomedical interventions targeting SARS-CoV-2 spike protein | non-relevant subject |
| 247 | Role of HLA-DP in the Presentation of Epitopes from the Truncated Bacterial PE38 Immunotoxin | non-relevant subject |
| 248 | SARS-CoV-2 Proteome Harbors Peptides Which Are Able to Trigger Autoimmunity Responses: Implications for Infection, Vaccination, and Population Coverage | non-relevant subject |
| 249 | Selection of conserved epitopes from hepatitis c virus for pan-populational stimulation of T-cell responses | non-relevant subject |
| 250 | Soluble HLA-associated peptide from PSF1 has a cancer vaccine potency | non-relevant subject |
| 251 | Statistical Linkage Analysis of Substitutions in Patient-Derived Sequences of Genotype 1a Hepatitis C Virus Nonstructural Protein 3 Exposes Targets for Immunogen Design | non-relevant subject |
| 252 | Structural and Epitope Analysis (T- and B-Cell Epitopes) of Hepatitis C Virus (HCV) Glycoproteins: An in silico Approach. | non-relevant subject |
| 253 | Structure and Computation in Immunoreagent Design: From Diagnostics to Vaccines | non-relevant subject |
| 254 | Structure to function analysis with antigenic characterization of a hypothetical protein, HPAG1_0576 from Helicobacter pylori HPAG1 | non-relevant subject |
| 255 | Structure-Based Design of Altered MHC Class II–Restricted Peptide Ligands with Heterogeneous Immunogenicity | non-relevant subject |
| 256 | Structure-guided design of multi-epitopes vaccine against variants of concern (VOCs) of SARS-CoV-2 and validation through In silico cloning and immune simulations | non-relevant subject |

| | | |
|---|---|---|
| 257 | Systemic inflammatory status predict the outcome of k-RAS WT metastatic colorectal cancer patients receiving the thymidylate synthase poly-epitope-peptide anticancer vaccine. | does not specify the use of neoepitope identification algorithms |
| 258 | T-cell epitope-based vaccine prediction against Aspergillus fumigatus: a harmful causative agent of aspergillosis. | non-relevant subject |
| 259 | Targeted Co-delivery of Tumor Antigen and $\alpha$-Galactosylceramide to CD141+ Dendritic Cells Induces a Potent Tumor Antigen-Specific Human CD8+ T Cell Response in Human Immune System Mice | does not specify the use of neoepitope identification algorithms |
| 260 | Targeted reconstruction of T cell receptor sequence from single cell RNA-seq links CDR3 length to T cell differentiation state | non-relevant subject |
| 261 | Targeting mutated plus germline epitopes confers pre-clinical efficacy of an instantly formulated cancer nano-vaccine | non-relevant subject |
| 262 | Targeting Neoepitopes to Treat Solid Malignancies: Immunosurgery | review |
| 263 | TCRmodel: high resolution modeling of T cell receptors from sequence | non-relevant subject |
| 264 | Tecemotide in unresectable stage III non-small-cell lung cancer in the phase III START study: Updated overall survival and biomarker analyses | review |
| 265 | Temporal Dynamics of the Primary Human T Cell Response to Yellow Fever Virus 17D As It Matures from an Effector- to a Memory-Type Response | non-relevant subject |
| 266 | Th17-inducing autologous dendritic cell vaccination promotes antigen-specific cellular and humoral immunity in ovarian cancer patients | does not specify the use of neoepitope identification algorithms |
| 267 | The de and FG loops of the HPV major capsid protein contribute to the epitopes of vaccine-induced cross-neutralising antibodies | viral infection-mediated cancer |

| | | |
|---|---|---|
| 268 | The Frequency of Naive and Early-Activated Hapten-Specific B Cell Subsets Dictates the Efficacy of a Therapeutic Vaccine against Prescription Opioid Abuse | non-relevant subject |
| 269 | The Frequency of Vaccine-Induced T-Cell Responses Does Not Predict the Rate of Acquisition after Repeated Intrarectal SIVmac239 Challenges in Mamu-B*08 + Rhesus Macaques | non-relevant subject |
| 270 | The genetic variability, phylogeny and functional significance of E6, E7 and LCR in human papillomavirus type 52 isolates in Sichuan, China | viral infection-mediated cancer |
| 271 | The Immunogenicity and Anti-tumor Efficacy of a Rationally Designed Neoantigen Vaccine for B16F10 Mouse Melanoma | does not specify the use of neoepitope identification algorithms |
| 272 | The Importance of Being Presented: Target Validation by Immunopeptidomics for Epitope-Specific Immunotherapies | review |
| 273 | The polymorphism analysis and epitope predicted of Alphapapillomavirus 9 E6 in Sichuan, China | viral infection-mediated cancer |
| 274 | The polymorphisms of LCR, E6, and E7 of HPV-58 isolates in Yunnan, Southwest China | viral infection-mediated cancer |
| 275 | The Serpin-like Loop Insertion of Ovalbumin Increases the Stability and Decreases the OVA 323-339 Epitope Processing Efficiency | non-relevant subject |
| 276 | The T Cell Epitope Landscape of SARS-CoV-2 Variants of Concern | viral model |
| 277 | Therapeutic cancer vaccines and translating vaccinomics science to the global health clinic: Emerging applications toward proof of concept | review |
| 278 | Therapeutic Vaccines Targeting Neoantigens to Induce T-Cell Immunity against Cancers. | review |
| 279 | This is not a pipe – But how harmful is electronic cigarette smoke | non-relevant subject |

| | | |
|---|---|---|
| 280 | Title: In vitro proteasome processing of neo-splicetopes does not predict their presentation in vivo | non-relevant subject |
| 281 | Transmitted/Founder HIV-1 Subtype C Viruses Show Distinctive Signature Patterns in Vif, Vpr, and Vpu That Are under Subsequent Immune Pressure during Early Infection | non-relevant subject |
| 282 | Uncovering the Tumor Antigen Landscape: What to Know about the Discovery Process. | review |
| 283 | Unique true predicted neoantigens (TPNAs) correlates with anti-tumor immune control in HCC patients | non-relevant subject |
| 284 | Unraveling the role of preexisting immunity in prostate cancer patients vaccinated with a HER-2/neu hybrid peptide | does not specify the use of neoepitope identification algorithms |
| 285 | Using homology modeling to interrogate binding affinity in neutralization of ricin toxin by a family of single domain antibodies | non-relevant subject |
| 286 | Utilizing Computational Machine Learning Tools to Understand Immunogenic Breadth in the Context of a CD8 T-Cell Mediated HIV Response | non-relevant subject |
| 287 | Vaccination with gag , vif , and nef Gene Fragments Affords Partial Control of Viral Replication after Mucosal Challenge with SIVmac239 | viral model |
| 288 | Vaccination with melanoma helper peptides induces antibody responses associated with improved overall survival | does not specify the use of neoepitope identification algorithms |
| 289 | Vesicular Stomatitis Virus Encoding a Destabilized Tumor Antigen Improves Activation of Anti-tumor T Cell Responses | non-relevant subject |
| 290 | Viral epitope profiling of COVID-19 patients reveals cross-reactivity and correlates of severity | non-relevant subject |
| 291 | Virus-specific T cells for adenovirus infection after stem cell transplantation are highly effective and class II HLA restricted | non-relevant subject |
| 292 | | |
| 293 | **Second exclusion** | |

| 294 | **Article** | **Exclusion reason** |
|---|---|---|
| 295 | A combination of epitope prediction and molecular docking allows for good identification of MHC class I restricted T-cell epitopes | viral model |
| 296 | A high-throughput yeast display approach to profile pathogen proteomes for MHC-II binding | fungal model |
| 297 | A human B cell receptor epitope-based erbB-2 peptide (N: 163-182) with pan-reactivity to the T cells of Japanese breast cancer patients | analysis of specific genes |
| 298 | A large peptidome dataset improves HLA class I epitope prediction across most of the human population | does not present a relevant computation al approach |
| 299 | A modified HLA-A*0201-restricted CTL epitope from human oncoprotein (hPEBP4) induces more efficient antitumor responses | analysis of specific genes |
| 300 | A neoepitope derived from a novel human germline APC gene mutation in familial adenomatous polyposis shows selective immunogenicity | analysis of specific genes |
| 301 | A New Epitope Selection Method: Application to Design a Multi-Valent Epitope Vaccine Targeting HRAS Oncogene in Squamous Cell Carcinoma. | analysis of specific genes |
| 302 | A novel in silico framework to improve MHC-I epitopes and break the tolerance to melanoma | wild-type peptide editing analysis |
| 303 | A pipeline for identification and validation of tumor-specific antigens in a mouse model of metastatic breast cancer | does not use human data |
| 304 | A Platform for Designing Genome-Based Personalized Immunotherapy or Vaccine against Cancer | does not use NGS data |
| 305 | Advanced Pancreatic Cancer Patient Benefit From Personalized Neoantigen Nanovaccine Based Immunotherapy: A Case Report | case study, does not focus on in silico analysis |
| 306 | An efficient T-cell epitope discovery strategy using in silico prediction and the iTopia assay platform | analysis of specific genes |

| | | |
|---|---|---|
| 307 | An immunogenic WT1-derived peptide that induces T cell response in the context of HLA-A*02:01 and HLA-A*24:02 molecules | analysis of specific genes |
| 308 | An in silico chimeric vaccine targeting breast cancer containing inherent adjuvant | analysis of specific genes |
| 309 | An oncofetal antigen, IMP-3-derived long peptides induce immune responses of both helper T cells and CTLs. | analysis of specific genes |
| 310 | Anti-tumor activity of a T-helper 1 multiantigen vaccine in a murine model of prostate cancer | does not present a relevant computation al approach |
| 311 | Application of mass spectrometry-based MHC immunopeptidome profiling in neoantigen identification for tumor | review |
| 312 | Aspartate-β-hydroxylase induces epitope-specific T cell responses in hepatocellular carcinoma | analysis of specific genes |
| 313 | Biochemical and functional characterization of mutant KRAS epitopes validates this oncoprotein for immunological targeting | analysis of specific genes |
| 314 | Case Report: Pathological Complete Response in a Lung Metastasis of Phyllodes Tumor Patient Following Treatment Containing Peptide Neoantigen Nano-Vaccine. | case study, does not focus on in silico analysis |
| 315 | CD171 Multi-epitope peptide design based on immuno-informatics approach as a cancer vaccine candidate for glioblastoma | analysis of specific genes |
| 316 | CD8 T cell function and cross-reactivity explored by stepwise increased peptide-HLA versus TCR affinity | analysis of specific genes |
| 317 | Combined assessment of MHC binding and antigen abundance improves T cell epitope predictions. | viral model |
| 318 | Combining STING-based neoantigen-targeted vaccine with checkpoint modulators enhances antitumor immunity in murine pancreatic cancer | murine model |

| | | |
|---|---|---|
| 319 | Comparative analysis of evolutionarily conserved motifs of epidermal growth factor receptor 2 (HER2) predicts novel potential therapeutic epitopes | analysis of specific genes |
| 320 | Computational Design of the Affinity and Specificity of a Therapeutic T Cell Receptor | does not present a relevant computation al approach |
| 321 | Computational prediction of vaccine potential epitopes and 3-dimensional structure of XAGE-1b for non-small cell lung cancer immunotherapy | analysis of specific genes |
| 322 | Cytotoxic T-lymphocyte elicited therapeutic vaccine candidate targeting cancer against MAGE-A11 carcinogenic protein | analysis of specific genes |
| 323 | Design of a new multi-epitope peptide vaccine for non-small cell Lung cancer via vaccinology methods: an in silico study | analysis of specific genes |
| 324 | Design of a Novel Recombinant Multi-Epitope Vaccine against Triple-Negative Breast Cancer | analysis of specific genes |
| 325 | Designing a Chimeric Vaccine Against Colorectal Cancer | analysis of specific genes |
| 326 | Designing a Novel Multi-epitope T Vaccine for ``Targeting Protein for Xklp-2'' (TPX2) in Hepatocellular Carcinoma Based on Immunoinformatics Approach | analysis of specific genes |
| 327 | Designing a novel SOX9 based multi-epitope vaccine to combat metastatic triple-negative breast cancer using immunoinformatics approach | analysis of specific genes |
| 328 | Development of a peptide-based vaccine targeting TMPRSS2:ERG fusion-positive prostate cancer | analysis of specific genes |
| 329 | DockTope: A Web-based tool for automated pMHC-I modelling | does not present a relevant computation al approach |
| 330 | Empirical Evaluation of the Use of Computational HLA Binding as an Early Filter to the Mass Spectrometry-Based Epitope Discovery Workflow. | does not present a relevant computation al approach |
| 331 | Enhanced anti-colon cancer immune responses with modified eEF2-derived peptides | wild-type peptide editing analysis |

| | | |
|---|---|---|
| 332 | Establishment of HLA-DR4 transgenic mice for the identification of CD4 + T cell epitopes of tumor-associated antigens | does not present a relevant computation al approach |
| 333 | Evaluating performance of existing computational models in predicting CD8+ T cell pathogenic epitopes and cancer neoantigens | does not present a relevant computation al approach |
| 334 | Experimental Study of Potential CD8+Trivalent Synthetic Peptides for Liver Cancer Vaccine Development Using Sprague Dawley Rat Models | does not address personalized therapy |
| 335 | Glypican-3-specific cytotoxic T lymphocytes induced by human leucocyte antigen-A*0201-restricted peptide effectively kill hepatocellular carcinoma cells in vitro. | analysis of specific genes |
| 336 | Graph-theoretical formulation of the generalized epitope-based vaccine design problem | does not present a relevant computation al approach |
| 337 | High sensitivity of cancer exome-based CD8 T cell neo-antigen identification | does not use NGS data |
| 338 | HLA class I restricted epitopes prediction of common tumor antigens in white and East Asian populations: Implication on antigen selection for cancer vaccine design | specific gene analysis |
| 339 | HLA-A2-restricted Cytotoxic T Lymphocyte Epitopes from Human Hepsin as Novel Targets for Prostate Cancer Immunotherapy | analysis of specific genes |
| 340 | HLA-binding properties of tumor neoepitopes in humans | review |
| 341 | Hotspots' of Antigen Presentation Revealed by Human Leukocyte Antigen Ligandomics for Neoantigen Prioritization. | does not address personalized therapy |
| 342 | Identification and translational validation of novel mammaglobin-A CD8 T cell epitopes | analysis of specific genes |
| 343 | Identification of a novel HLA-A*02:01-restricted cytotoxic T lymphocyte epitope derived from the EML4-ALK fusion gene | analysis of specific genes |
| 344 | Identification of a Promiscuous Epitope Peptide Derived from HSP70 | analysis of specific genes |

| | | |
|---|---|---|
| 345 | Identification of an H2-Kb or H2-Db restricted and glypican-3-derived cytotoxic T-lymphocyte epitope peptide | analysis of specific genes |
| 346 | Identification of CD8+ T-cell epitope from multiple myeloma-specific antigen AKAP4 | analysis of specific genes |
| 347 | Identification of CDCA1-derived long peptides bearing both CD4+ and CD8+ T-cell epitopes: CDCA1-specific CD4+ T-cell immunity in cancer patients | analysis of specific genes |
| 348 | Identification of cross-reactive CD8+ T cell receptors with high functional avidity to a SARS-CoV-2 immunodominant epitope and its natural mutant variants. | viral model |
| 349 | Identification of glypican-3-derived long peptides activating both CD8+ and CD4+ T cells; prolonged overall survival in cancer patients with Th cell response. | analysis of specific genes |
| 350 | Identification of HLA-A*1101-restricted cytotoxic T lymphocyte epitopes derived from epidermal growth factor pathway substrate number 8 | analysis of specific genes |
| 351 | Identification of HLA-A24-restricted CD8 + cytotoxic T-cell epitopes derived from mammaglobin-A, a human breast cancer-associated antigen | analysis of specific genes |
| 352 | Identification of human leukemia antigen A*0201-restricted epitopes derived from epidermal growth factor pathway substrate number 8 | analysis of specific genes |
| 353 | Identification of Immunogenic MHC Class II Human HER3 Peptides that Mediate Anti-HER3 CD4 Th1 Responses and Potential Use as a Cancer Vaccine | analysis of specific genes |
| 354 | Identification of Immunogenic MHC Class II Human HER3 Peptides that Mediate Anti-HER3 CD4 Th1 Responses and Potential Use as a Cancer Vaccine | analysis of specific genes |
| 355 | Identification of Neoantigens in Two Murine Gastric Cancer Cell Lines Leading to the Neoantigen-Based Immunotherapy | murine model |

| | | |
|---|---|---|
| 356 | Identification of new HLA-A*0201-restricted cytotoxic T lymphocyte epitopes from neuritin | analysis of specific genes |
| 357 | Identification of Prostate-Specific G-Protein Coupled Receptor as a Tumor Antigen Recognized by CD8+ T Cells for Cancer Immunotherapy | analysis of specific genes |
| 358 | Identification of Special AT-Rich Sequence Binding Protein 1 as a Novel Tumor Antigen Recognized by CD8+ T Cells: Implication for Cancer Immunotherapy | analysis of specific genes |
| 359 | Identifying T Cell Receptors from High-Throughput Sequencing: Dealing with Promiscuity in TCRα and TCRβ Pairing | does not present a relevant computation al approach |
| 360 | Immunogenicity of Del19 EGFR mutations in Chinese patients affected by lung adenocarcinoma | analysis of specific genes |
| 361 | Immunoinformatics Approach to Design T-cell Epitope-Based Vaccine | viral model |
| 362 | Implementation of Vaccinomics and In-Silico Approaches to Construct Multimeric Based Vaccine Against Ovarian Cancer | analysis of specific genes |
| 363 | In silico analysis, molecular docking, molecular dynamic, cloning, expression and purification of chimeric protein in colorectal cancer treatment | analysis of specific genes |
| 364 | In silico and cell-based analyses reveal strong divergence between prediction and observation of T-cell-recognized tumor antigen T-cell epitopes | does not present a relevant computation al approach |
| 365 | In silico approach in designing a novel multi-epitope vaccine candidate against non-small cell lung cancer with overexpressed G protein-coupled receptor 56 | analysis of specific genes |
| 366 | In silico design and evaluation of PRAME+FliC δD2D3 as a new breast cancer vaccine candidate | analysis of specific genes |
| 367 | In silico design of a triple-negative breast cancer vaccine by targeting cancer testis antigens | analysis of specific genes |
| 368 | In silico design of discontinuous peptides representative of b and t-cell epitopes from her2-ecd as potential novel cancer peptide vaccines | analysis of specific genes |

| | | |
|---|---|---|
| 369 | In silico evaluation of PLAC1-flic as a chimeric vaccine against breast cancer | analysis of specific genes |
| 370 | In silico prediction of B cell epitopes of the extracellular domain of insulin-like growth factor-1 receptor. | analysis of specific genes |
| 371 | In silico-guided sequence modifications of K-ras epitopes improve immunological outcome against G12V and G13D mutant KRAS antigens. | wild-type peptide editing analysis |
| 372 | Integrating CD4+ T cell help for therapeutic cancer vaccination in a preclinical head and neck cancer model | analysis of specific genes |
| 373 | Key Parameters of Tumor Epitope Immunogenicity Revealed Through a Consortium Approach Improve Neoantigen Prediction | does not present a relevant computation al approach |
| 374 | Linear and conformational B cell epitope prediction of the HER 2 ECD-subdomain III by in silico methods. | analysis of specific genes |
| 375 | Metadherin peptides containing CD4+ and CD8+ T cell epitopes as a therapeutic vaccine candidate against cancer | analysis of specific genes |
| 376 | MHC class I loaded ligands from breast cancer cell lines: A potential HLA-I-typed antigen collection | does not use NGS data |
| 377 | Mismatch Repair Deficiency Drives Durable Complete Remission by Targeting Programmed Death Receptor 1 in a Metastatic Luminal Breast Cancer Patient. | does not present a relevant computation al approach |
| 378 | MUC1 glycopeptide epitopes predicted by computational glycomics | analysis of specific genes |
| 379 | Mutant MHC class II epitopes drive therapeutic immune responses to cancer | murine model |
| 380 | Novel peptide-based vaccine targeting heat shock protein 90 induces effective antitumor immunity in a HER2+ breast cancer murine model | analysis of specific genes |
| 381 | Novel Predicted B-Cell Epitopes of PSMA for Development of Prostate Cancer Vaccine | analysis of specific genes |
| 382 | Optimized polyepitope neoantigen DNA vaccines elicit neoantigen-specific immune responses in preclinical models and in clinical translation | murine model |

| | | |
|---|---|---|
| 383 | Pan-cancer analysis of neoepitopes | does not present a relevant computational approach |
| 384 | Peptide FLNPDVLDI of heparanase is a novel HLA-A2-restricted CTL epitope and elicits potent immunological antitumor effects in vitro with an 8-branched-design | analysis of specific genes |
| 385 | Population-level distribution and putative immunogenicity of cancer neoepitopes | does not use NGS data |
| 386 | Predicting T cell recognition of MHC class I restricted neoepitopes | does not use NGS data |
| 387 | Presence of antigen-specific somatic allelic mutations and splice variants do not predict for immunological response to genetic vaccination. | does not present a relevant computational approach |
| 388 | Recurrent Frameshift Neoantigen Vaccine Elicits Protective Immunity With Reduced Tumor Burden and Improved Overall Survival in a Lynch Syndrome Mouse Model | murine model |
| 389 | Residue substitution enhances the immunogenicity of neoepitopes from gastric cancers | wild-type peptide editing analysis |
| 390 | Robust prediction of HLA class II epitopes by deep motif deconvolution of immunopeptidomes | closed |
| 391 | Role of in silico structural modeling in predicting immunogenic neoepitopes for cancer vaccine development | murine model |
| 392 | Screening of Human Epidermal Growth Factor Receptor 2 (HER2) Extracellular Domain for Potential Epitopes by Using Immuno-informatics Tools | analysis of specific genes |
| 393 | Shared Immunogenic Poly-Epitope Frameshift Mutations in Microsatellite Unstable Tumors | does not present a relevant computational approach |
| 394 | Structural Analysis and Epitope Prediction of MHC Class-1-Chain Related Protein-A for Cancer Vaccine Development. | analysis of specific genes |

| | | |
|---|---|---|
| 395 | Structural Features of Antibody-Peptide Recognition | does not present a relevant computational approach |
| 396 | Systematically benchmarking peptide-MHC binding predictors: From synthetic to naturally processed epitopes | does not use mutation data |
| 397 | TANTIGEN 2.0: a knowledge base of tumor T cell antigens and epitopes | database construction |
| 398 | Targeting Tumor Markers with Antisense Peptides: An Example of Human Prostate Specific Antigen | analysis of specific genes |
| 399 | TCR contact residue hydrophobicity is a hallmark of immunogenic CD8(+) T cell epitopes | viral model |
| 400 | The Cancer Epitope Database and Analysis Resource: A Blueprint for the Establishment of a New Bioinformatics Resource for Use by the Cancer Immunology Community | does not present a relevant computational approach |
| 401 | Tumor neoantigens: Building a framework for personalized cancer immunotherapy | review |
| 402 | Two novel squamous cell carcinoma antigen-derived HLA-A <sup>*</sup>0201-binding peptides induce in vitro and in vivo CD8 <sup>+</sup> cytotoxic T lymphocyte responses | analysis of specific genes |
| 403 | USMPep: universal sequence models for major histocompatibility complex | viral model |
| 404 | Vaccine candidate designed against carcinoembryonic antigen-related cell adhesion molecules using immunoinformatics tools | analysis of specific genes |
