# Supplementary material for: Tumor Neoepitope-Based Vaccines: A Scoping Review on Current Predictive Computational Strategies
Source: Vaccines (Basel). 2024 Jul 24;12(8):836. doi: 10.3390/vaccines12080836 (PMC11360805; doi:10.3390/vaccines12080836)
Supplement: Supplementary file 1 [file vaccines-12-00836-s001.zip › Table S3_ HLA-I alleles groups.pdf]

HLA-I allele	Reference	Group
A*02:01	18, 19, 21, 22, 24, 25, 31, 32, 33, 34, 35, 38, 39, 40	A
A*01:01	18, 19, 24, 25, 31, 32, 33, 35, 39	B
A*03:01	18, 21, 31, 32, 33, 35, 37, 38, 39	B
A*11:01	18, 21, 31, 32, 33, 34, 38	C
B*07:02	18, 21, 25, 31, 34, 35, 39	C
C*07:02	18, 21, 25, 31, 32, 33, 34	C
A*24:02	18, 31, 33, 34, 35, 38	D
B*08:01	18, 24, 31, 32, 35, 39	D
B*27:05	18, 21, 31, 32, 35, 40	D
B*35:01	18, 32, 33, 35, 37, 39	D
C*15:02	18, 21, 31, 32, 34	E
A*68:01	18, 31, 32, 35, 39	E
B*40:01	18, 31, 32, 33, 34	E
B*44:02	18, 24, 31, 32, 35	E
A*26:01	18, 31, 32, 34	F
B*15:01	18, 31, 39, 40	F
B*18:01	18, 19, 31, 35	F
B*44:03	18, 21, 31, 35	F
B*51:01, C*02:02	18, 21, 31, 32	F
C*01:02	18, 31, 32, 33	F
C*04:01	18, 31, 32, 37	F
A*02:06	18, 34, 38	G
A*30:01	18, 33, 38	G
A*32:01	18, 35, 40	G
A*33:03	18, 21, 38	G
B*35:02	18, 32, 35	G
B*35:03	18, 31, 35	G
B*40:06, C*08:01	18, 33, 34	G
C*03:02	18, 25, 33	G
C*03:03	18, 31, 33	G
C*03:04	18, 31, 32	G
C*05:01	18, 19, 33	G
C*06:02	18, 31, 33	G
C*07:04	18, 31, 32	G
C*08:02	18, 31, 33	G
A*02:02, A*25:01, A*30:02	18, 35	H
A*02:05, B*38:01, C*12:03	18, 32	H
A*23:01, B*14:01, A*29:02, B*37:01, B*55:01, C*07:01, C*16:01	18, 31	H
A*29:01	18, 37	H
A*31:01	18, 34	H
A*33:01	18, 39	H
A*022	20, 26	H
B*07:05	18, 37	H
B*13:02	18, 33	H

B*15:18	33, 34	H
B*39:01	18, 34	H
B*45:01	18, 19	H
B*52:01	18, 33	H
B*54:01	33, 34	H
B*55:02	18, 33	H
B*58:01	18, 25	H
C*12:02	18, 34	H
C*14:02	18, 21	H
C*15:05	18, 37	H
A*02:07, A*02:11, A*02:17, A*03:19, A*11:02, A*23:05, A*24:01, A*24:11, A*26:02, A*26:03, A*26:13, A*30:04, A*34:02, A*36:04, A*66:01, A*66:02, A*68:02, A*68:03, A*68:12, A*74:01, A*24, B*14:02, B*14:06, B*15:02, B*15:03, B*15:05, B*15:07, B*15:10, B*15:16, B*15:17, B*15:80, B*27:02, B*27:03, B*27:04, B*27:12, B*27:34, B*35:08, B*35:14, B*35:86, B*37:04, B*37:19, B*39:06, B*40:02, B*40:05, B*41:01, B*41:02, B*42:01, B*44:05, B*44:07, B*46:01, B*47:01, B*48:01, B*49:01, B*50:01, B*51:08, B*51:12, B*53:01, B*56:01, B*56:29, B*57:01, B*57:03, B*58:05, B*78:01, B*81:03, C*04:04, C*04:21, C*07:06, C*08:04, C*08:05, C*08:22, C*14:03, C*16:02, C*17:01, C*18:02	18	I
